# A Review of the Tribology of 3D-Printed Polymers and Polymer Composites

**DOI:** 10.3390/polym18212747

**Published:** 2026-11-04

**Authors:** Andrei Călin, Georgiana Ionela Păduraru, Delia Alexandra Prisecaru, Lucian Cucu, Marilena Stoica

**Affiliations:** Department of Machine Elements and Tribology, National University of Science and Technology Politehnica Bucharest, 060042 Bucharest, Romania; gipaduraru@upb.ro (G.I.P.); delia.prisecaru@upb.ro (D.A.P.); lucian.cucu@upb.ro (L.C.); marilena.stoica@upb.ro (M.S.)

**Keywords:** additive manufacturing, 3D printing, tribology, friction, wear, polymer composites, fused filament fabrication, surface topography, anisotropy, specific wear rate

## Abstract

3D-printed polymers and polymer composites are increasingly used in sliding and abrasive contacts, but their friction and wear cannot be transferred directly from data for moulded polymers, because printing creates layered interfaces, process-specific surface textures and position-dependent crystallinity. This systematised critical review analyses 289 experimental studies and grades the evidence for each step linking the printing process to internal structure, surface, contact conditions, wear mechanisms and component life. Four findings stand out. First, the orientation of layers and rasters relative to sliding is associated with a switch between wear controlled by shear of interlayer bonds and wear controlled by crossing of surface ridges; coding orientation as {contact face, layer normal, raster/fibre direction} explains several apparently contradictory results. Second, test conditions dominate absolute wear rates, which for printed neat PEEK span 2.6 decades, with the largest contrast between contact configurations, so effects must be compared within studies. Third, only 3 of 245 full-text studies report all core contact and statistical information; a minimum reporting set is proposed. Fourth, only three printed-versus-conventional comparisons isolate the manufacturing route. Established findings include the failure of hardness as a general wear predictor and friction reduction by oil and grease; lubricant storage in designed porosity remains probable.

## 1. Introduction

### 1.1. Printed Polymers in Functional Contacts

Polymer additive manufacturing (AM) produces end-use parts from thermoplastics, photopolymers and composites [1,2,3], many in sliding, rolling or abrasive contact: gears [4,5,6], PEEK journal bearings [7,8], dental restorations and occlusal devices [9,10,11] and orthopaedic bearing materials [12,13]. Printing also makes the contact surface designable (morphing surfaces, damping lattices, graded multi-material contacts) [14,15,16,17]. Tribological losses are costly [18], and the durability of printed restorations is already a clinical concern (Section 6.2) [19]. The literature is growing quickly (215 of the 289 experimental studies in the present evidence base date from 2023 or later, derived) but is fragmented by process, material and test method.

### 1.2. Why Bulk Polymer Tribology Does Not Transfer

Polymer tribology [20,21,22,23,24,25] was built on moulded or extruded material with a near-homogeneous bulk and an independently prepared surface; a printed part has neither. It is a stack of welds, formed by neck growth and chain interdiffusion in a short thermal window and generally weaker than the bulk [26,27,28,29,30,31], so sliding can shear interlayer interfaces [32,33], and its infill-set porosity governs wear where infill is exposed (Section 8.3) [34]. Its contact face carries a process-generated topography (raster ridges, stair-steps, sintered particles or an extrusion skin) that controls friction at low load and during running-in (Section 9 and Section 10.3) [35,36,37,38,39]. Its crystallinity or degree of conversion follows thermal history and can be graded within one part [40]; annealing lowered the wear of printed PEEK but raised that of PLA in the same study [41]. Even nominally identical filaments differed in COF (Section 8.4) [42].

### 1.3. Prior Reviews and the Scope of This Review

Reviews of AM tribology across all materials are dominated by metals: only 19 of the 105 tribology papers synthesised by Orgeldinger et al. [43] concerned polymers, and other reviews treat mainly or only alloys [44,45,46]. Recent polymer-specific reviews treat FDM and SLA parts narratively [47] or condense FDM filler effects into general rules [48], and reviews of the mechanical properties of printed polymers [30,31] do not address contact. Three gaps follow. Among the prior reviews identified, none extracts test conditions systematically, so contradictory results remain unexplained by load, counterface or orientation; none grades the strength of evidence, so replicated findings and single-study observations carry equal weight; and none audits whether the published data are reported completely enough to be compared. This review addresses these gaps for all polymer AM routes and composites, using full-text extraction of test conditions, study-level appraisal and explicit evidence grades (Section 2).

### 1.4. Aim, Framework and Research Questions

The aim of this paper is to establish how printing route, parameters and post-processing set the friction and wear of printed polymers and composites, and why published results disagree; metal and ceramic AM are covered in a companion review. The analysis follows the chain process → architecture → surface integrity → contact conditions → friction and wear mechanisms → durability, grading the support for each link (Figure 1).

The review makes four principal contributions. C1 codes orientation as the triplet {contact face, layer normal, raster/fibre direction} relative to sliding (Section 8). C2 replaces pooled absolute wear rates, which for one printed polymer span more than two orders of magnitude across test conditions [34,41,49], with within-study effect sizes (Section 16). C3 audits reporting practice and proposes a minimum reporting set (Section 11). C4 classifies printed-versus-conventional comparisons as material-matched, product-matched or not material-matched and draws conclusions about the route only from a route-isolated subset (Section 15) [50,51,52,53]. Secondary contributions are friction–wear decoupling and the limits of hardness as a wear predictor (S1; Section 14) [11,50], porosity as lubricant reservoir or fluid-ingress path (S2; Section 13) [54,55,56,57,58,59] and a wear-mechanism map by material class and contact severity (S3; Section 12).

Research questions (answering sections in parentheses): RQ1, printed versus conventionally processed polymers (15); RQ2, the most influential process parameters and their pathways (8); RQ3, topography versus bulk control (9, 10, 14); RQ4, layer, raster and fibre anisotropy (7.8, 8, 9); RQ5, mechanisms unique to or intensified by layered architectures (12); RQ6, whether post-processing acts on roughness or bulk (10); RQ7, why nominally similar studies disagree (8, 11, 16, 18); RQ8, what prevents reliable design prediction (14, 16, 18, 19).

**Figure 1:**
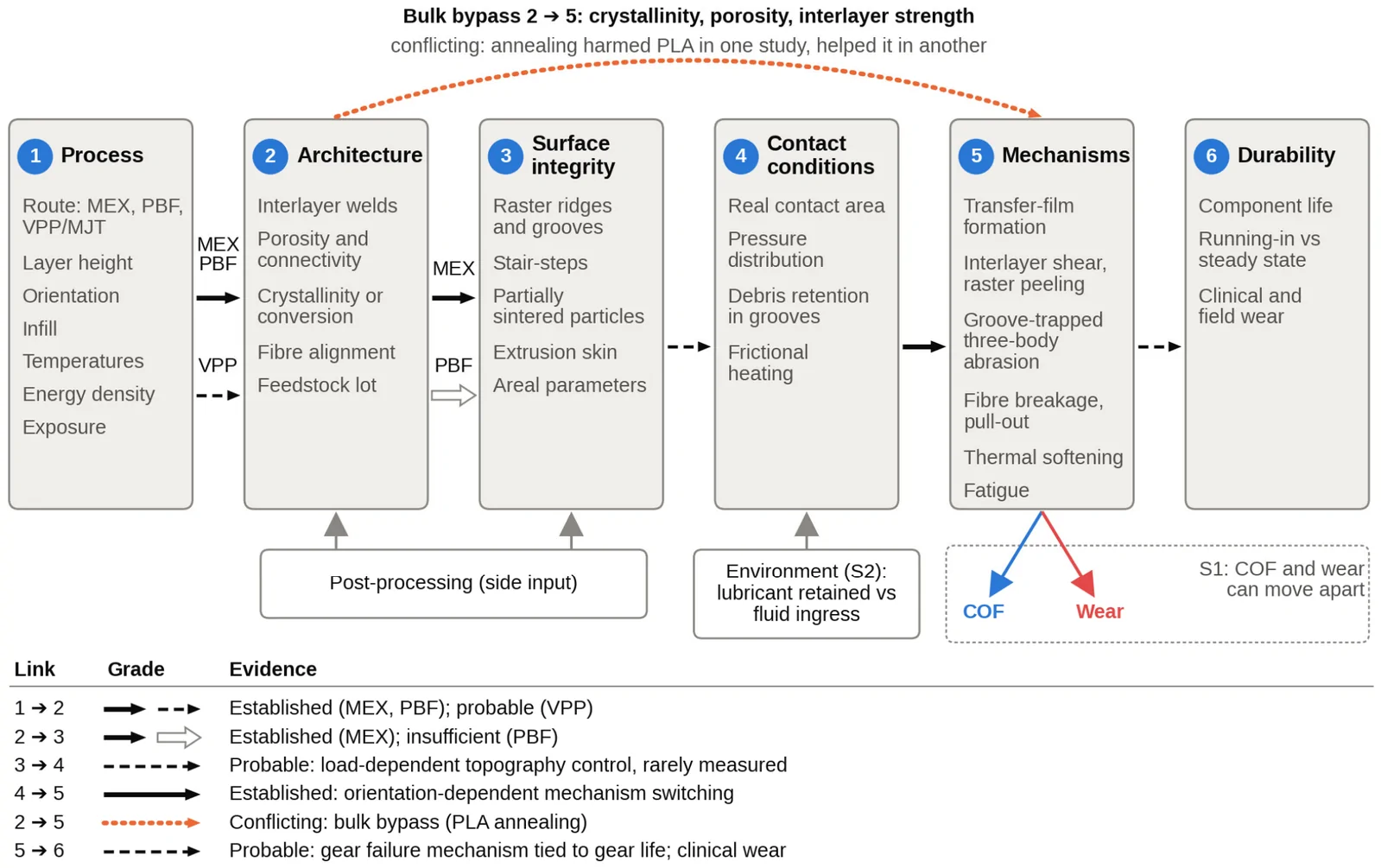
Framework linking printing process to tribological durability of printed polymers and composites, with the strength of evidence for each link. Arrow style encodes the evidence grade defined in Section 2.5 (solid black, established; dashed black, probable; dotted orange, conflicting; open grey, insufficient); where a link differs by process, both grades are shown. The upper arc is the bulk bypass from architecture to mechanisms; side inputs are post-processing and environment, and the S1 inset marks that friction and wear can move in opposite directions. Representative studies for each link: 1 → 2 [26,27,29,34,40,60]; 2 → 3 [39]; 3 → 4 [35,61]; 4 → 5 [32,33,36,62]; 2 → 5 [41]; 5 → 6 [4,19]; the full evidence for each link is given in Section 18.1.

## 2. Review Methodology

### 2.1. Review Type and Search Strategy

This work is a systematised critical review and evidence synthesis: it uses documented searches, explicit eligibility criteria, full-text extraction and study-level appraisal, but is neither a systematic review, since selection was not exhaustive, nor a meta-analysis: within-study ratios are summarised descriptively as unweighted medians and ranges (Section 16), but no variance-weighted pooled effect is estimated. Web of Science (All Databases; title and author keywords) and Europe PMC (including full-text matching) were searched in 2026 (last search on 29 September 2026), combining additive manufacturing, 3D printing and process names with tribology, friction, wear, abrasion and erosion. A supplementary OpenAlex search (last search on 30 September 2026) was restricted to Elsevier and MDPI articles because their full texts were accessible. The review was conducted and is reported in accordance with the PRISMA 2020 guidelines, as far as they apply to a systematised review, and the completed PRISMA 2020 checklist is provided in the Supplementary Materials. Departures from a full systematic review (prioritised, non-exhaustive selection; appraisal and grading by one reviewer; no registered protocol; no meta-analysis) are stated in Section 2.1, Section 2.2, Section 2.3, Section 2.4 and Section 2.5.

### 2.2. Eligibility, Screening and Selection

Eligible records were peer-reviewed journal articles measuring friction, wear, abrasion or erosion of an additively manufactured specimen; friction-based AM processes, AM producing only a coating or repair layer, and non-tribological uses of “wear” or “friction” were excluded. After removal of 695 Web of Science and 275 Europe PMC records before screening, 2869 and 2096 records were screened on title and abstract, and 1605 and 23 were eligible (Figure 2).

From this pool, a core set prioritised by journal tier, citations per year, quantitative reporting and section coverage, an open-access extension of openly readable eligible records and the Europe PMC additions formed an all-material base of 441 studies; moving 269 metal, metal-matrix and ceramic studies to a companion review left 172 polymer studies (72, 95 and 5, respectively), and 89 more were added from the eligible pool for topic coverage. OpenAlex added 34 studies after title, abstract and full-text assessment of 1028 new records, and one study identified through expert knowledge after database screening passed the same full-text eligibility and appraisal criteria (screening log). Journal tier and citation rate served only to prioritise records for extraction and entered no appraisal or grade. The 296 included studies (172 + 89 + 34 + 1) comprise 289 experimental studies and 7 reviews cited only for positioning; with 59 foundational and context references, the evidence base holds 355 records, each verified against Web of Science, Europe PMC, Crossref or the publisher’s archive, of which 342 are in the reference list (331 cited in the main article, including its figures, and 11 only in the Supplementary Materials).

Selection was therefore prioritised rather than exhaustive and partly driven by full-text access, so counts and percentages describe the 245 full-text experimental studies analysed, not the whole literature, and statements of absence mean “not identified in the present evidence base”. Representativeness was checked against the Web of Science eligible pool on which the prioritisation acted (Table S5). Process names were searched alongside the generic terms, so retrieval did not depend on the route. Of 427 eligible polymer records (route assigned from title, abstract and keywords), the 264 included and 163 not included did not differ in publication period (χ^2^(4) = 2.0, *p* = 0.73), and their route mix differed modestly, with a borderline test result (χ^2^ = 10.1, permutation *p* = 0.05): material extrusion made up 51% and 59%, powder bed fusion 10% and 4%, and vat photopolymerisation 8% and 5%, so the coverage criterion, if anything, over-represented the minority routes. Representativeness beyond this Web of Science pool cannot be assumed. Included records were cited more often (median 3.4 versus 1.0 citations per year, *p* < 0.001), as prioritisation intends; Section 2.5 and Section 16.3 test whether this biased the findings. Across the 289 experimental studies, material extrusion accounts for 60%, vat photopolymerisation for 19% (mostly dental), powder bed fusion for 11%, material jetting for 2% and direct ink writing for 1% (7% unstated); findings for the minority routes rest on fewer studies, and their grades reflect this.

### 2.3. Data Extraction

All abstracts were extracted, and the 250 full-text records (245 experimental studies) were re-extracted for print parameters, orientation triplet, contact conditions, environment, roughness of both surfaces, specific wear rate, replication and bulk state. Numbers are quoted only from full texts; implausible, predicted or unit-ambiguous values indicate direction only, and calculated values are labelled “derived”.

### 2.4. Study Appraisal: Reporting and Evidential Completeness

Each full-text study was scored 0–5 on five criteria: complete test conditions (load, speed, distance or duration, counterface material), surface characterisation (specimen or counterface roughness), replication with dispersion, outcome quantified in physical units and mechanism evidence beyond SEM (chemical or subsurface analysis), met by 68.6%, 47.3%, 42.0%, 85.3% and 20.4% of the 245 studies, respectively. Scores of 4–5, 2–3 and 0–1 define high (48 studies), moderate (138) and low (59) appraisal classes; implausible values cap a study at low. The score measures reporting and evidential completeness rather than methodological rigour as such, because the fifth criterion rewards mechanistic depth; scoring only the first four criteria moves 2 of the 186 studies with moderate or high appraisal to low and changes no evidence grade. For grading, research-group independence was approximated operationally: studies sharing at least two authors were assigned to one group (176 groups), a proxy that can both merge and split laboratories. Per-study scores are in Table S4.

### 2.5. Evidence Grading

Findings and links are graded as established (consistent direction in ≥3 studies from ≥2 independent research groups, at least two with moderate or high appraisal, and no moderate/high-appraisal study in the opposite direction under comparable conditions), probable (one or two studies with moderate or high appraisal, or ≥3 studies from a single research group, with a plausible mechanism), conflicting (moderate/high-appraisal studies disagree in direction under comparable conditions) or insufficient (no study identified, or only abstract-level and/or low-appraisal evidence). Low-appraisal and abstract-level studies may indicate direction but cannot by themselves raise a grade. The grading thresholds are author-defined operational criteria intended to make the synthesis transparent and consistent, not a validated risk-of-bias instrument. Grades were assigned by one reviewer applying these criteria; inter-rater agreement was not measured. Their internal consistency and sensitivity were therefore tested on the 81 claims of a grade audit (80 with a single grade, plus the cells of the mechanism map), using the supporting and opposing studies listed for each claim (Table S6). Applied mechanically, the rule reproduced all 24 established grades. All 24 remained established when three moderate- or high-appraisal studies from three groups were required, or when appraisal used only the first four criteria; 21 remained when at least one high-appraisal study was required, 22 when research groups were merged at a single shared author (121 instead of 176 groups), and 17 under all stricter variants combined. Relaxing the rule to two studies from two groups would raise 14 of the 27 probable grades, mostly for mechanistic explanations that no study has tested directly. Wear rates from different test conditions are not pooled; comparisons use within-study ratios (Section 16).

### 2.6. Use of Generative Artificial Intelligence

Generative AI (Claude, Anthropic; model Claude Opus 5.5, used through the Claude application in July–October 2026) was used to assist with literature screening, data extraction, figure preparation and language editing during the preparation of this manuscript. The authors checked all eligibility decisions, extracted values, evidence grades, figures, references and interpretations against the original sources and revised the output as needed.

## 3. Polymer AM Processes from a Tribological Perspective

Each process family leaves a characteristic contact-scale architecture (Table 1, Figure 3) [1,2,3]. The process–architecture link is established for material extrusion and powder bed fusion and probable for vat photopolymerisation; the architecture–surface link is established only for material extrusion (Section 18.1).

### 3.1. Material Extrusion

In fused filament fabrication (FFF), bead–bead interfaces form by non-isothermal neck growth and interdiffusion, so weld strength and crystallinity follow each interface’s thermal history [26,27,28,63]. The surface is a periodic ridge–valley texture set by layer height and road width (Table 1; Section 9.1) [64,65,66]. Interbead voids initiate subsurface cracking, bead separation and layer peeling [64,67,68], and weld planes cause tensile and compressive anisotropy [29,30,31,69]. An extrusion skin on the outer bead probably lowers friction (Section 10.3) [39].

Short fibres align with the deposition path, so raster and fibre directions coincide in the orientation triplet [70,71,72], and they raise porosity [53]. In continuous-fibre co-extrusion, fibres normal to the contact face wore fastest [73,74], reflecting the end-on penalty established for FFF in dry, moderate-to-severe contact (Section 7.8 and Section 8.1). Pellet extrusion coarsens the texture further (Table 1) [75,76].

### 3.2. Powder Bed Fusion

In selective laser sintering (SLS) and multi jet fusion (MJF), consolidation within a narrow sintering window leaves residual porosity and unmelted particle cores [77,78], and partially sintered particles roughen the surface [79,80,81,82]. Without rasters, anisotropy is interface-controlled and face-dependent: polished vertical MJF PA12 faces showed directional COFs, while horizontal faces did not [36], so directional friction in powder bed fusion is probable. Evidence on build orientation is conflicting (Section 8.1) [83,84]: SLS PA12 wear was largely orientation-independent [85], yet in SLS TPU, abrasion detached one-layer-thick plates by interlayer fatigue cracking [86].

In laser PBF UHMWPE, wear fell about tenfold as relative density rose (Section 10.6) [60]. In PBF PEEK composites, higher crystallinity from repeated scanning lowered wear but raised the COF, with porosity co-varying (probable, one research group; Section 8.6) [40,87]. In PBF CF/PEEK, the face exposing fibre ends wore least [6], reversing the FFF ranking at far milder contact; a severity crossover is probable (Section 7.8).

### 3.3. Vat Photopolymerisation and Material Jetting

Vat photopolymerisation (SLA, DLP, LCD/MSLA) and material jetting give the smoothest as-built surfaces, but only on layer-parallel faces: inclined DLP faces were an order of magnitude rougher [88,89], and among printed dental guides, the masked-SLA guide had the most uniform surface [90]. The process-specific variable is the degree of conversion, which post-cure raises [91]; nitrogen post-curing reduced nano-wear depth (Section 10.2) [92], but evidence for conversion as a wear descriptor is insufficient (Section 6.2) [93]. Multi-material jetting adds rigid–soft laminates and load-adaptive textures that moulding cannot produce [94,95].

**Figure 3:**
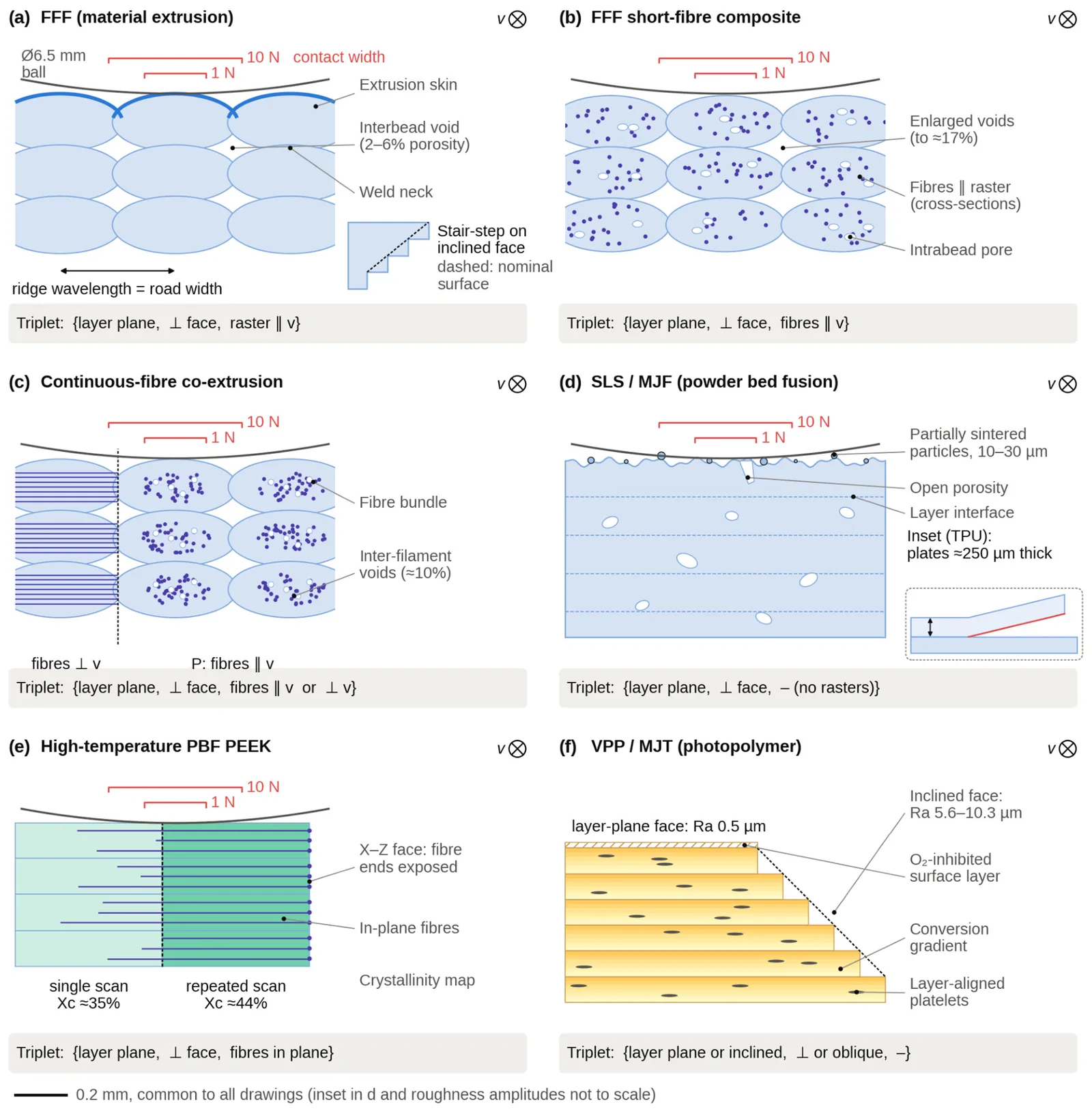
Process-specific architectures that reach the contact, shown as schematic cross-sections normal to the sliding direction for the principal polymer AM routes. Sources: (**a**) extrusion skin [39] and interbead voids [64]; (**b**) enlarged voids [53]; (**c**) inter-filament voids [73]; (**d**) partially sintered particles [79] and TPU plates [86]; (**e**) exposed fibre ends [6] and crystallinity map [40,87]; (**f**) inclined-face roughness [88], oxygen-inhibited surface layer and conversion gradient [92] and layer-aligned platelets [89]. Contact widths at 1 and 10 N were derived for the test conditions of [35].

### 3.4. Direct Ink Writing

Direct ink writing (DIW) allows texture to be designed rather than tolerated. Printed PDMS textures and helicoidal CF/epoxy outperformed cast comparators (Section 15.2) [96,97], and a MoS_2_-rich gradient top layer reduced polyimide wear by 68% [98]. A benefit of DIW-designed architecture is therefore probable (two moderate-appraisal studies).

**Table 1:** Polymer AM processes and their tribological signatures.

Process	Typical Polymers	Architecture Features	As-Built Surface Signature	Expected Tribological Consequence	Representative Studies
FFF (filament)	PLA, ABS, PETG, PA, PEEK, PC, TPU	Interbead voids (2.1–6.4% [64]); weld planes; extrusion skin; crystallinity set by envelope temperature	Periodic ridges; Ra 2.7 µm (top/infill face) to 13–19 µm (shell, inclined, thick layers) [66,82]; Sa 8–19 µm [64,65]	Directional COF at low load (established); ridge flattening in running-in; void-initiated bead separation and layer peeling; skin probably lowers the COF	[27,29,39,64,66,67]
FFF, short-fibre composites	CF/GF-PA, CF-PEEK, CF-PLA	Fibres aligned with raster; porosity up to 16.5–17.9% [53]	Sq 8.4–12.4 µm vs. 5.6 µm neat PEEK [53]	Fibre direction enters the orientation triplet; fibre ends abrade the transfer film; void pits	[53,70,99]
Continuous-fibre co-extrusion	CF/PA6, Onyx	Inter-filament voids ≈ 10% [73]; fibre-rich/matrix-rich banding	Not reported (specimens polished)	End-on fibres wear most (2.7–7.8× in SWR, derived)	[73,74]
Pellet/screw extrusion	PA6/CF, PETG composites	Wide roads; porosity set by fibre–matrix adhesion (1.90–4.85% [76])	Ra 16.6–27.8 µm (direct-pellet composites) [75]	Coarser texture; interfacial debonding in weakly bonded systems	[75,76,100]
SLS	PA12, glass-filled PA12, TPU	Residual porosity; partially sintered particles 10–30 µm [79]; layer interfaces	Ra ≈ 10–13 µm [80,81,82]; Sa 17–21 µm on gear flanks [101]	Particle-dominated running-in; build-orientation effect conflicting, weaker on wear than on the COF; interlayer delamination in elastomers	[81,84,85,86]
MJF	PA12, PA12 glass-bead	As SLS, with fusing-agent-defined layers	Ra 9.7 µm (PA12GB) [102]	COF anisotropy (up to 60%) on faces exposing layer interfaces (probable)	[36,83,102]
High-temperature laser PBF	PEEK and PEEK composites, UHMWPE	Crystallinity (≈35–44%) and porosity (2.5–13.3%) set by scan strategy [40,87]; relative density ≈ 61–90% [60]	Sa 27–29 µm (PEEK/MoS_2_) [87]	Crystallinity trades the COF against wear (probable, one group); density controls wear (≈10×)	[6,40,60,87,103]
SLA/DLP/LCD	Acrylate and methacrylate resins, dental composites, PU	Degree of conversion and post-cure; oxygen-inhibited surface layer; layer-aligned fillers	Ra 0.5 µm on layer-plane faces vs. 5.6–10.3 µm on inclined faces [88]; Sa 3.2–4.8 µm [104]	Stair-step-controlled running-in on inclined faces; conversion as a wear descriptor insufficiently evidenced	[88,89,91,92,93]
Material jetting (PolyJet)	Rigid and elastomeric photopolymers	Multi-material voxels; support-contact surfaces	Ra 0.03–0.08 µm along vs. 0.44–0.58 µm across raster [35]; Ra 2.7 µm [82]	Raster-direction friction at low load (probable); designed rigid–soft and textured surfaces	[35,82,94,95]
DIW	PDMS, epoxy/CF, polyimide	Cure-fixed filaments; designed filament paths and gradients	Designed filament texture (not quantified)	Designed textures and gradients; tribological benefit probable	[96,97,98]

Roughness values are as-built (or as-cleaned/sandblasted for [80,102]) and are given only where full-text data report them; parameters differ between studies (Ra, Sa, Sq, RMS) and are not interchangeable. Ratios marked “derived” were calculated from the cited data.

## 4. Polymer Tribology Fundamentals Meet Layered Architecture

### 4.1. Adhesion and Deformation Components of Friction

Polymer friction comprises an adhesive term (interfacial shear strength times real contact area) and a deformation term (ploughing and viscoelastic hysteresis), both rate- and temperature-dependent, also on printed substrates [20,23,86,105,106]. Printing adds ridge ploughing and infill compliance: the COF of FFF TPU varied non-monotonically with infill (Section 8.3) [107], and against compliant counterparts, texture geometry set friction [96,108]. The deformation term therefore depends on subsurface architecture, not only on loss modulus.

### 4.2. Transfer Films

Steady-state dry wear of thermoplastics against metals is governed by the formation, adhesion and renewal of a transfer film, established for mild wear of printed thermoplastics (Section 12.1) [21,109,110,111]. On printed parts, the film is often discontinuous and abraded by carbon fibres [53,64], prevented by fibre ends facing the counterface [73] and regulated by crystallinity (Section 14.2) [40,87]. Debris trapped in raster and layer valleys acts as a third body (established; Section 12.3) [35,88], but no study reviewed here resolved whether it lubricates or starves film supply. Film theory assumes uniform supply, whereas printed surfaces supply material periodically.

### 4.3. Real and Apparent Contact on Printed Topography

Greenwood–Williamson and Persson models assume random asperities [112,113,114], whereas printed surfaces are periodic, anisotropic and skewed, with texture wavelengths comparable to the contact width (Section 9.1) [64]. Directional friction on as-printed faces is established for FFF and probable for material jetting, and its loss with rising load is established [35,66,115,116], but evidence for a transferable threshold is insufficient (Section 9.2). Running-in smoothing of FFF texture is established [61,64,65,66,117], although SLS bearing surfaces hardly changed [81]; that topography governs running-in more than steady state is probable.

### 4.4. Wear Laws, pv Limits and Frictional Heating

Archard’s law scales wear inversely with hardness [113], the Lancaster–Ratner correlation inversely with the strength–elongation product [22,118], and delamination theory attributes wear to subsurface cracking [119]. Printed hardness varies with face and density [36,60] and alone does not predict wear across printed polymer systems and regimes (established; Section 14.1) [65,104,120]. The relevant strength is probably that of the weakest weld: a nozzle temperature rise that raised tensile strength cut the specific wear rate (Section 8.4) [39], and delamination depth matched layer thickness [86]. Running-in and pressure-dependent wear coefficients make wear non-linear [64,73,121]. pv limits from moulded stock do not transfer: contact temperatures approached Tg in SLS PA12 bearings [81,85] and caused thermal failure of FFF gears [101], whereas a DIW CF/epoxy architecture conducted heat better than its moulded control [97].

## 5. Tribology of Printed Thermoplastics

Within one test programme, the polymer usually outweighs print settings (material explained 59.76% of wear-volume variance, layer thickness 21.32%) [122], so each subsection identifies the controlling state (interlayer weld, porosity, crystallinity, skin or thermal limit; studies in Table S1). Parameters, post-processing, environment and conventional comparisons are treated in Section 8, Section 10, Section 13 and Section 15.

### 5.1. Poly(Lactic Acid) (PLA)

PLA softens near 60 °C, so sustained dry sliding is thermally limited: pins failed by plastic flow and bearings deformed thermally [123,124], and at 14 N, PLA lost 23–25 times (derived) more mass than ABS or PETG [65]. Below this limit, the interlayer weld is the weakest shear path and pores initiate cracks (Section 8.1) [66,116,125,126]. Annealing effects conflict (Section 10.1) [41,127].

### 5.2. ABS and ASA

Rubber-toughened ABS deforms rather than fractures and wore at least 10 times (derived) less than PLA [122]. Its weak point is porosity: subsurface cracks nucleated at voids and separated beads [64]. Hardness and modulus did not rank wear (Section 14) [128], and ABS lost the most mass of six polymers in two-body abrasion [129]. Sliding across layer lines raised the COF [32,130] but minimised wear [32] (Section 8.1), and in ASA, thicker layers raised the COF by about 65% [131].

### 5.3. PETG

In PETG, the print atmosphere alters surface chemistry: printing in air rather than N_2_ oxidised the polymer and thickened the transfer film, and N_2_-printed pins wore 58% more, whereas supplier and layer orientation had no significant effect [51]. As these pins were polished and other studies report only the COF, grooving or scratch friction [68,132], evidence on as-printed PETG topography is insufficient.

### 5.4. Polyamides

In FFF polyamide, the melt-cooled skin controls friction (Section 10.3), whereas pores and welds control wear: a higher nozzle temperature cut the specific wear rate by 75.5% [39]. Weak interlayer cohesion raised abrasive mass loss by up to 52% with alternating rasters [133] and caused interlayer peeling in all but the most crystalline printed nylon gears [4]. Powder-bed PA12 is more isotropic: neat SLS PA12 wore at 3.20–4.78 × 10^−4^ mm^3^/(N·m) by adhesion and layer-shaped fatigue cracking [134], as in two studies with unverifiable values [135,136], and build-orientation effects were small or condition-specific (Section 3.2 and Section 8.1) [36,83,84,85,86]. In SLS PA11, low energy density left void-initiated microcracks [137].

### 5.5. PEEK and PEI

In FFF PEEK, crystallinity and interlayer densification control wear: annealing lowers dry wear (established, conditional on load and filament direction) while the COF sometimes rises (Section 10.1 and Section 14.2) [41,138,139,140]. Lower infill and layers ≥ 0.25 mm act oppositely, the latter trapping debris that converts two-body into three-body abrasion (Section 8) [34,141]. These within-study effects, 0.15–0.71 decades (derived), are small beside the 2.6-decade spread of absolute specific wear rates across studies, the largest contrast being between contact configurations (Section 16.2) [6,49,142]. Unlike PLA and ABS, PEEK wore least, with filaments aligned with sliding (Section 8.1) [140,143]. No printed PEKK study was identified. For PEI, path-strategy effects on abrasion reversed between X and Z builds [144,145], and self-mated gears ran at ≈60 °C, but gears against steel ran at 32–33 °C [5,121], so the mate’s heat removal probably sets the regime.

### 5.6. TPU and Other Elastomers

Printed elastomers wear by viscous deformation and fatigue rather than cutting [146], and friction follows effective stiffness, which infill sets non-monotonically and grade-specifically (probable; non-standard rigs, few replicates; Section 8.3) [107,147]. Hardness did not rank wear [147,148], SLS TPU shed layer-thick plates by interlayer fatigue (Section 3.2) [86], and polymer choice outweighed lattice density in abrasion [149].

### 5.7. UHMWPE, PE and PP

In L-PBF UHMWPE, wear fell as density rose (Section 10.6) [60]; textured FFF UHMWPE reached a COF of 0.075 in serum (Section 9) [150], and FFF LDPE wear barely depended on path direction [151]. PP has only abrasion data [50]; no dry-sliding study of neat printed PP or HDPE was identified.

### 5.8. PC, HIPS and Others

Self-mated PC smoothed rapidly along filaments under boundary or early mixed lubrication, yet 45° surfaces 5–10 times rougher gave a similar COF [61]; PC gears [152] and sliding HIPS [122] each wore most among three printed materials. PPS shows the PLA-type thermal limit, its specific wear rate rising 37-fold (derived) from 100 to 196 N [153], and PMMA has abrasion data only [50].

*Key findings.* The state that controls wear differs by polymer: the thermal limit and the interlayer weld for PLA, porosity for ABS, print-induced surface chemistry for PETG, the skin and interlayer cohesion for FFF polyamides, crystallinity and densification for PEEK, and effective stiffness for elastomers. Within single test programmes, polymer choice outweighs print settings. Only the annealing benefit for FFF PEEK is established for an individual polymer; most other polymer-specific statements rest on one or two studies and should be read as system-specific.

## 6. Tribology of Printed Photopolymers

Vat photopolymerisation and material jetting produce cross-linked networks whose state is set by layer and by interface conversion rather than melt welding [3]. Engineering and dental studies (Table S2) differ on whether formulation and cure or build angle control wear.

### 6.1. Engineering Resins

Unfilled resins sliding dry on steel reach a steady-state COF of 0.43–0.76 (established; six studies), with ploughing, tearing and cracking perpendicular to sliding [35,88,89,94,154,155]. Resin chemistry outweighs processing: resin type explained 73.57% of mass-loss variation in masked-SLA pins, washing and post-cure explained ≤2.29% each [120], and a PolyJet resin doubled the COF of FDM and SLS polymers [82]. On as-printed surfaces, build angle acts mainly through topography (Section 9): inclined DLP builds raised Ra 11–20-fold (derived) at an unchanged COF [88], a 0° build minimised SLA resin wear [156], and PolyJet orientation effects vanished above 1 N as bulk response took over [35].

Lubricant supply, not stiffness, gives the largest gains (Section 13.5): PFPE-loaded silica cut the COF from ≈0.7 to ≈0.1 and the reported wear rate by 97.7% [154], with large COF reductions also from PAO droplets and oil-impregnated lattices [56,157]. Nanoparticles show narrow optima (Section 7) [155,158,159] and can interact with build angle: 0.5 wt% graphene reduced wear depth in horizontal builds but raised it in 45° and vertical builds, attributed from worn-surface SEM to edge-on platelets [89]. Multi-material jetting enables directional or switchable friction [14,94,95,160]; application studies [161,162,163] and irreconcilable SiC-filled resin data [164] add little mechanism.

### 6.2. Dental Resins

Dental studies supply most photopolymer evidence and three established findings. First, formulation dominates process settings: filled DLP denture-tooth resin wore over 20 times less than unfilled SLA resin (derived) [165], whereas layer thickness (50–100 µm) had no significant effect (probable) [166,167]. Filler content matters [168] but does not rank resins alone [169]: the most highly filled and converted crown resin wore most [93], and hardness tracked wear in opposite directions (Section 14) [104,170]. Filler–matrix interface quality and network toughness are plausible controls, but neither has been measured alongside wear (insufficient).

Second, post-cure lowers wear (established; Section 10.2) [92,167,171,172]; that it does so through the degree of conversion is insufficiently evidenced [120,173].

Third, printed-versus-conventional rankings depend on resin class, wear mode and duration (Section 15). Rigid printed resins matched conventional comparators [10,11,174,175,176,177,178], but flexible splint resins wore 6.1–7.0 times more than milled PMMA (derived) [11]. Rankings also shifted with cycle number [179] and test method [180,181,182], and between two- and three-body wear, consistent with the lower filler fraction of printed crown resins [173]. Comparisons with more highly filled milled materials contrast formulations, not processes [183,184].

Printed resins caused less antagonist wear than lithium disilicate or zirconia [185,186] and wore less than primary enamel [187]. Under cyclic contact, they failed by subsurface fatigue cracking, delamination and interlayer separation; this rests on SEM or optical microscopy of worn surfaces in four studies [174,185,188,189] and on direct subsurface imaging by optical coherence tomography in one [93] (Section 12.6). Clinically, 35% of printed definitive restorations exceeded 0.5 mm height loss by 24 months [19], and toothbrushing causes milligram-level loss [190,191].

Build-angle evidence is conflicting, with significant [9,167,181,192] versus marginal effects [165,193]. On flat polished blocks, the effects, −21% [167] and −27% [193] (both derived), are close to what n = 8–10 designs resolve, because polishing removes the stair-steps that drive engineering results [88]. Larger effects arise where the angle probably also alters geometry, supports or exposed interior: 90° crowns lost 43% more volume in vivo than 0° crowns (derived) [9], and splint rings differed up to 5.2-fold (derived) where wear penetrated the polished skin into a porous interior [181]. This explanation is probable; resins built at 0°, 45° and 90°, polished to equal Sa and slid parallel and perpendicular to the layers, would test it.

### 6.3. What Dental Testing Can Teach Engineering Tribology

Dental studies use standardised simulators, 10^5^–10^6^ cycles, n = 8–10, 3D-scan volumetry and antagonist wear [10,169,193], all rare in engineering studies (Section 11). Their weaknesses also instruct: polished specimens exclude the printed surface [10,179], wear metrics and clinical-time equivalences are inconsistent [176,194,195,196], and coefficients of variation reached 85–104% (derived) [175]. Engineering studies should adopt dental replication and antagonist reporting; dental studies should add the COF, as-printed surfaces, specific wear rate and the orientation triplet.

*Key findings.* In photopolymers, formulation (resin chemistry, filler fraction) and post-cure outweigh print settings; build angle acts mainly through stair-step topography on as-printed faces and only weakly after polishing. The fatigue-type failure of printed dental resins is well documented on worn surfaces but has been imaged below the surface in only one study, and the network properties proposed to control it have not been measured alongside wear.

## 7. Tribology of Printed Polymer Composites

Fillers either carry load and resist ploughing (fibres, hard particles) or supply a low-shear transfer film (PTFE, lamellar solids, oils), as in moulded composites [25,109,111,197]. Printing adds filler orientation set by deposition or spreading [70], porosity around the filler and exposed fibre ends, which decide whether reinforcement helps or harms. Table 2 compares representative fillers and their within-study outcomes (further studies in Table S3), and Figure 4 summarises when fillers help or harm.

**Table 2:** Structured comparison of fillers in printed polymers: content, size, matrix and contact conditions, with within-study changes relative to the unfilled printed reference (ratio filled/neat; <1 = reduction).

Filler (Size)	Content	Matrix/ Process	Contact	COF Ratio	Wear Ratio	Interface, Dispersion and Mechanism	Refs
Short CF (Ø7 µm × 150 µm)	15 wt%	PEEK/PBF	Ball-on-plate, 5 N, 5 mm/s, dry	0.73	0.05 (SWR)	Fibres carry load at low severity; end-on face wore least	[6]
Short CF (Ø7 µm × 100–150 µm)	5 wt%	PEEK/FFF	Ball-on-block, 30 N, water	0.60–0.79	≈0.1	Load support persists in water	[99]
Short CF (Ø7 µm × 100 µm), ±nano-SiO_2_ (10–20 nm)	10 wt% (+2 wt%)	PEEK/FFF	Flat-on-flat, 5–10 MPa, 0.02 m/s, dry	0.53 (with SiO_2_)	≈1 (SCF, 10 MPa); 0.39 (with SiO_2_)	SCF alone gave no wear gain; nano-SiO_2_ stabilised the transfer film	[142]
Short CF + Si_3_N_4_ + MoS_2_	10 + 2 + 3 wt%	PEEK/FFF	Ball-on-flat, 50–100 N, 0.3 m/s, dry	0.50–0.80	1.21 (depth, 50–75 N); 0.79 (100 N)	Interfacial fatigue and fibre debonding; SCF-PEEK wore most at all loads	[198]
Glass fibre (Ø13 µm × 200 µm)	10 and 15 wt%	PLA/FFF	Ball-on-flat, 10–20 N, dry	1.18–1.25 (10 N)	2.06–10.5 (10 N); 0.22 (15 wt%, 20 N)	Third bodies in mild wear; load support once neat PLA wears severely	[58]
β-Si_3_N_4_ (30 µm)	10 wt%	PEEK/FFF	Flat pin-on-disc, 96 N, 0.12 m/s, PBS	≈1.4	≈0.5	Counterface roughened to Sz 5–10 µm	[13]
Hollow ceramic spheres (20–75 µm)	20 wt%	PA11/SLS	Ball-on-disc, 3 N, 0.07 m/s, dry	–	0.54 (SWR)	Same energy density as neat	[137]
PTFE (≤5 µm)	7.5 wt%	PEEK/FFF	Ball-on-flat, 30 N, dry	0.39	lower up to 10 wt%	Agglomerates and pores beyond 10 wt%	[199]
PTFE (3 µm)/graphite (5–10 µm)/MoS_2_ (4.5 µm)	1 wt%	PA12/SLS	Ball-on-disc, 10 N, 0.35 m/s, dry	0.63/0.95/1.00	1.12/0.58/0.22	PTFE film broke down thermally at 30 N; lamellar solids kept load support	[134]
h-BN (10 µm)	10 wt%	PEEK/PBF	Ball-on-flat, 50 N, 20–200 °C	–	0.58 (depth, 20 °C)	Dispersion limit 15 wt%	[103]
Graphene; GNP (D50 25 µm)	0.3 wt%; 10 wt%	PLA/FFF; PA12/SLS	Reciprocating, 10 N; ball-on-flat, 2 N	0.39; 0.73	0.39 (mass); 0.19 (volume)	Carbon-rich transfer layer; ≤0.1 wt% graphene lowered friction only	[80,200,201]
CNT (Ø60–100 nm × 5–15 µm)	0.7 and 0.9 wt%	PPS/FFF	Block-on-ring, 100–196 N, 0.42 m/s	–	0.27 (0.7 wt%, 100 N); 0.02 (0.9 wt%, 196 N)	Suppressed the transition to severe wear	[153]
Nano-SiO_2_ with SiC	1 + 6.5 wt%	PA12/SLS (recycled powder)	Ball-on-disc, 20 N, seawater	≈1.3	highest of the composites	No dry reference; loose third bodies probable	[79]
Oil-loaded silica (50 nm, PFPE); talc + PAO	20 wt%; 10 + 7 wt%	Acrylate/VPP; cyanate ester/DIW	Ball-on-flat, 5–10 N, dry	0.14; 0.17	0.02 (rate); 0.14 (volume)	Lubricant release; talc without oil raised wear 4.4-fold	[154,157]

Ratios are within-study values from Supplementary Dataset S1 or were derived here from the reported values; “lower” where only the direction is reported; – not reported. Supplementary Dataset S1 holds one prespecified comparison per study and condition, so some ratios shown here (e.g., CNT/PPS at 196 N, oil-loaded silica) are not part of it and the ranges summarised in Section 16.3 differ. Interfacial bonding was characterised only indirectly (surface treatment, porosity, SEM of pull-out) in these studies.

### 7.1. Short Carbon, Glass and Aramid Fibres

In engineering matrices, short carbon fibre (SCF) lowers the COF (established) [6,53,59,99,202,203], but its wear effect is conflicting (Table 2): the SWR fell by an order of magnitude in powder-bed PEEK and water-lubricated FFF PEEK [6,99], yet in dry FFF PEEK, it matched neat PEEK [142] or wore most of four materials [198]. Three explanations compete: interface quality, contact severity and fibre orientation relative to sliding (Section 7.8). Interface quality matters, as grafted fibres lowered the COF and porosity [76] and stronger interlayer bonding lowered wear [100], but no study varied interfacial strength under the high-load conditions where SCF failed.

Commodity matrices also conflict: under mild contact, filled PA6.6, PETG and ASA often wore at least as much as unfilled references and scratched the steel counterface [204,205,206,207]; glass fibre raised PLA wear until neat PLA wore severely [58].

### 7.2. Continuous Fibres

No continuous-fibre study reviewed here reports a dry SWR against an unfilled printed control; the CF/PA6 evidence concerns fibre orientation [73,74] (Section 7.8 and Section 8.1), and there is insufficient evidence for better water tolerance than that of short fibres [208,209].

### 7.3. Solid Lubricants and Embedded Liquid Lubricants

PTFE and lamellar solids lower the COF through counterface transfer films (established) [91,103,134,136,199,210], but wear falls only if the matrix supports the film: in SLS PA12, PTFE raised the SWR as its film broke down thermally, whereas MoS_2_ and graphite lowered it [134]. In load-bearing PEEK, PA12 and DLP matrices, the COF fell by 52–73% and wear by 42–92% (derived ranges) up to a dispersion limit set by agglomerates or micropores (Table 2) [91,103,136,199,210]; crystallinity probably adds a trade-off (lower SWR, higher COF) in LPBF PEEK composites [40,87] (Section 14.2). Graphite helped [211,212] except where flake–matrix cracking dominated at 8.4 m/s [67]. Embedded liquids give the largest reductions [154,157,213] (Section 6.1), but depletion rates are unreported.

### 7.4. Carbon Nanofillers

Graphene, GNPs and CNTs lower the COF in most printed matrices [80,200,214], but wear falls only with sufficient loading or load support (Table 2): 0.3–0.5 wt% graphene in PLA and 10 wt% GNPs in SLS PA12 reduced material loss [80,200], ≤0.1 wt% graphene lowered only friction [201,215], and CNTs suppressed the severe-wear transition in PPS [153].

### 7.5. Ceramic and Metal Particles

Hard particles reduced printed-body wear by ≈46–93% (partly derived) [137,151,216], or by 20–54% in micro-SiC/PA12 depending on the metric [135], but counterface and friction data reveal penalties that pin-only metrics hide [13,217,218]: β-Si_3_N_4_ roughened the CoCr counterface and raised the COF (Table 2), and metal or Al_2_O_3_ fillers scratched steel or raised the COF at low load (Section 12). Bronze-PLA data were irreconcilable [16], and nylon–metal composites were referenced to ABS [219,220,221]. Silver filler also made the layer thickness of printed PLA more variable [222].

Particle size and environment raise a second contradiction: nano-SiO_2_ lowered the COF and wear of SCF-PEEK and an SLA resin in dry sliding [142,159,223], but in seawater raised the COF and had the highest wear of the SLS PA12 composites (micro-SiC the lowest) [79], though without a dry reference and with recycled powder. Water probably suppresses film formation and flushes debris [13,53], leaving nanoparticles as loose third bodies, as released SiO_2_ behaved dry at 5 MPa [223]; dry and immersed tests with a neat reference and debris sizing would resolve this.

### 7.6. Bio-Based Fillers

Biocarbon and corn cob reduced PLA wear with little change in the COF [224,225], but other bio-fillers lack neat controls or report implausible wear values [226,227,228,229,230] (insufficient evidence).

### 7.7. Hybrid Fillers

Combining load-bearing and film-forming or interface-stabilising phases gave the best PEEK results, nano-SiO_2_ in SCF-PEEK cutting the COF and SWR by up to 50% and 60% versus neat PEEK [142], but synergy is load-dependent: SCF/Si_3_N_4_/MoS_2_-PEEK lowered the COF yet wore 21% deeper than neat PEEK at 50 and 75 N [198], and PLA and ABS hybrids traded one property for another [218,231]. With only two constituent-matched studies [91,198], synergy remains probable.

### 7.8. Competing Mechanisms and Fibre Orientation

Of the three explanations for the conflicting SCF results, fibre orientation (the triplet; Section 8) is best supported: end-on fibres, aligned with the contact normal, wear most in dry FFF composites under moderate-to-severe contact (established), doubling the COF and raising the SWR ≈ 4.1× (derived) relative to parallel fibres in PEEK/SCF/graphite on plate-on-ring (50–100 N, 1–4 m/s) [62,232], as in continuous CF/PA6 and transverse-fibre abrasion [73,74]. Orientation alone cannot explain the reversal in PBF PEEK/SCF, where at 5 N and 5 mm/s, end-on fibres acted as micro-pillars and wore least (−28% SWR, derived) [6]: the triplets match, so labelling is not the cause. Contact severity is the probable second factor: end-on fibres carry load until tip bending reaches a fracture threshold half that of in-plane fibres (25 vs. 50 mN in micro-scratching) [233], and F·v in the PBF study is over 10^3^ times lower (derived). Interface quality is the third and least tested factor: SCF-PEEK wore most where fibres debonded [198] and grafting reduced porosity and friction [76], but interfacial strength was never measured alongside wear. One composite tested in all three triplets over an F·v sweep spanning both studies, at two levels of fibre–matrix adhesion, would separate the factors.

Fillers thus help when the neat matrix wears severely and the filler carries load [6,58,153] or feeds a coherent film [103,136,199]; they harm in mild wear [58,198,204,206], through counterface damage [13,207,217], beyond the dispersion limit [80,103,199,216] or when water or ageing degrades the interface [57] (Section 13.4; Figure 4). Ageing can also destabilise friction with little change in wear [58]; harm is under-reported because counterface wear was rarely quantified (Section 11.3).

**Figure 4:**
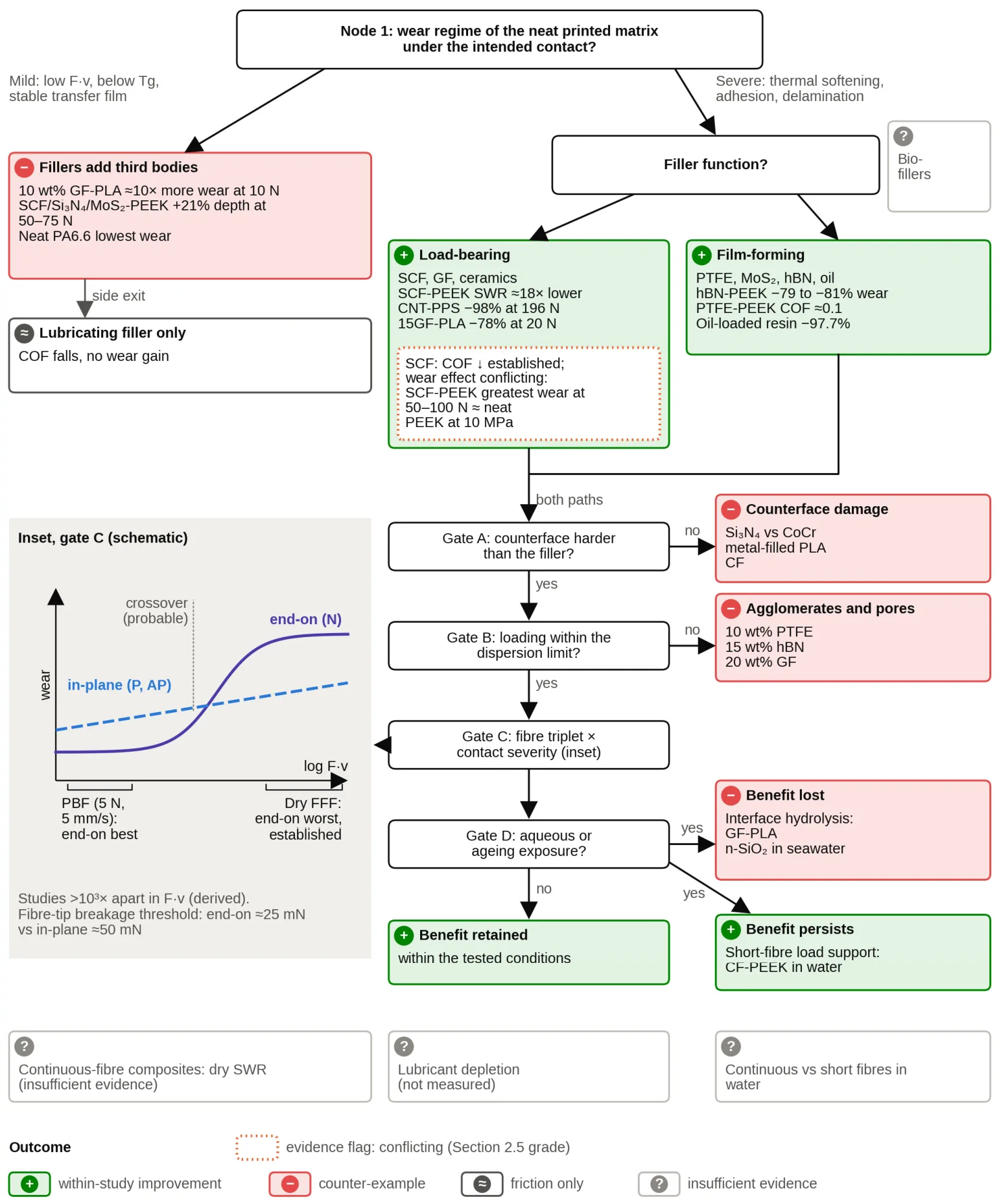
Decision diagram for when fillers improve or impair the tribological performance of printed polymer composites. Green paths end in within-study improvements and red paths in counter-examples; a neutral box marks friction-only gains and grey boxes insufficient evidence; each terminal box states the effect sizes behind it; and an inset sketches the probable fibre-orientation crossover with contact severity. Supporting studies: fillers adding third bodies [58,198,204]; lubricating filler only [134,201]; load-bearing fillers [6,58,153]; conflicting SCF wear [142,198]; film-forming fillers [103,154,199]; counterface damage [13,207,217]; agglomerates and pores [103,199,216]; benefit lost [57,58,79]; benefit persists [53]; continuous versus short fibres in water [208]; inset: PBF [6], dry FFF [62,73,232] and fibre-tip breakage thresholds [233].

## 8. Influence of Process Parameters: A Cross-Material Synthesis

Process parameters are interpreted here as acting through intermediate states (weld strength, porosity, crystallinity or cure, fibre orientation, topography) that one nominal change can move in opposite directions; Table 3 grades each parameter without pooling absolute wear rates (Section 16). Expert-elicited rankings that place infill among the top factors for FFF print quality [234] therefore do not transfer to friction and wear, where infill beneath a solid skin had little effect (Section 16.3).

### 8.1. The Orientation Triplet

The first principal contribution of this review (C1) is to code orientation as the triplet {contact face, layer normal, raster/fibre direction} relative to the sliding direction, separating configurations reported under one label (Figure 5) that conflates two process parameters (build orientation and raster or fibre direction) with a test parameter (sliding direction). Of the 245 full-text experimental studies analysed, 43 state the triplet fully and 114 leave it indeterminable (Section 11).

**Figure 5:**
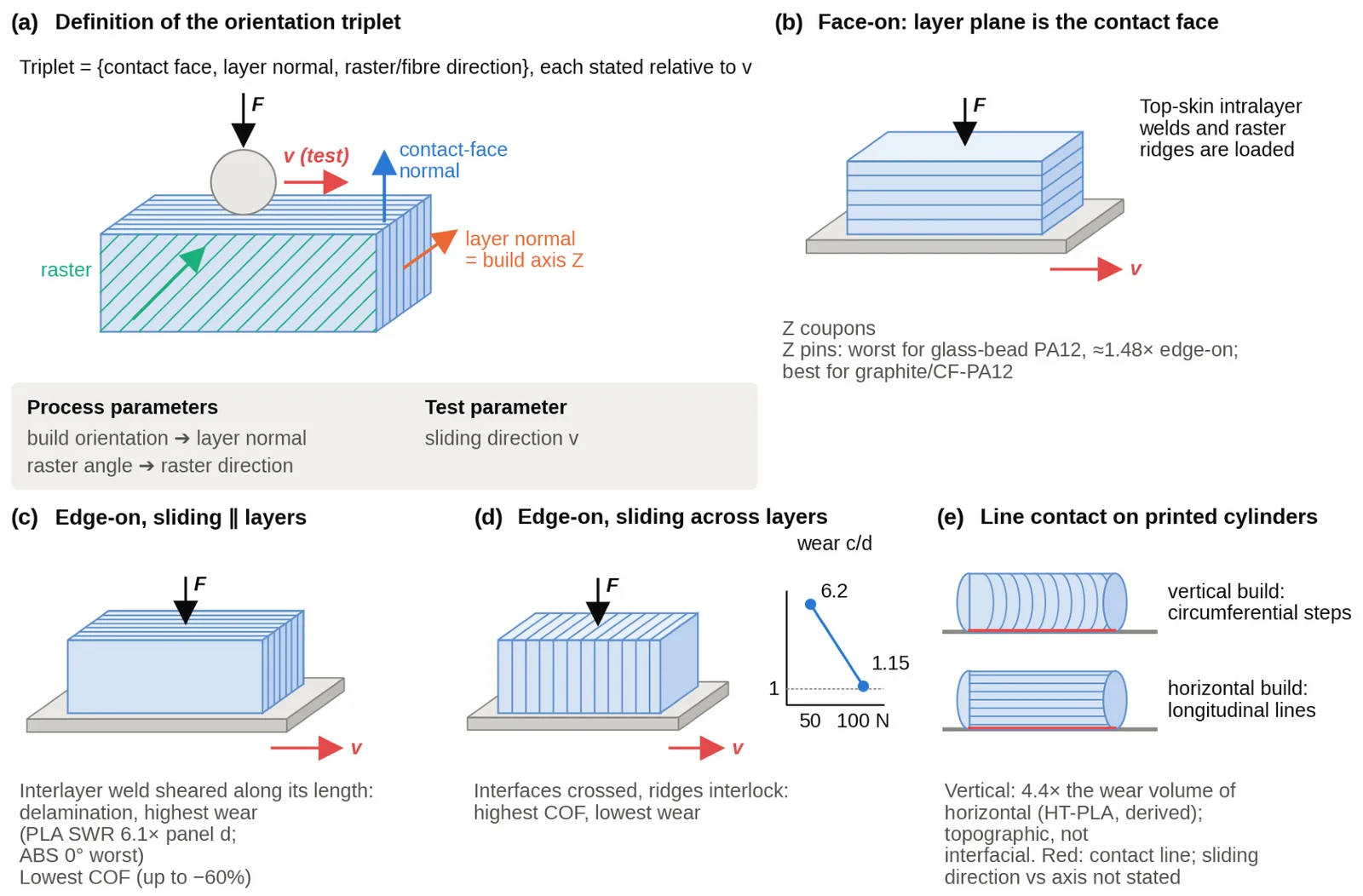

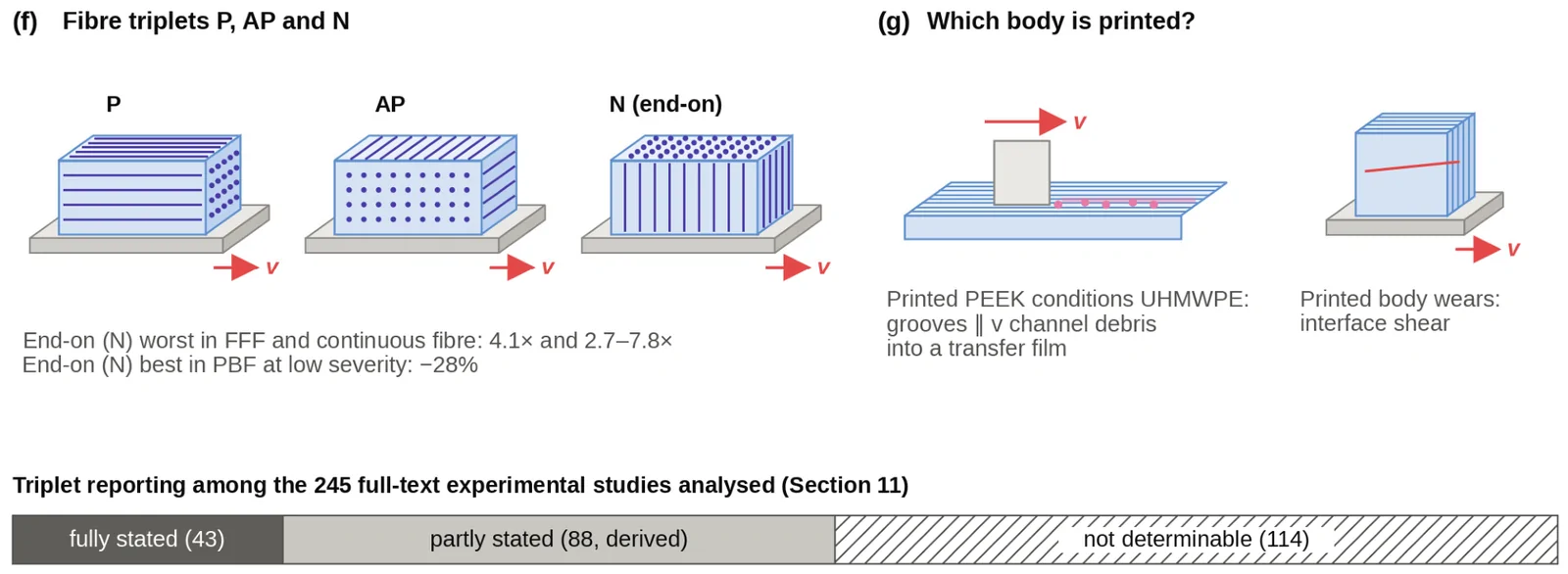
The orientation triplet: each printed specimen is described by {contact face, layer normal, raster/fibre direction} relative to the sliding direction. The interface that is sheared, crossed or used as a debris channel differs between configurations that the literature labels with the same word (“vertical”, “parallel”). Sources: (**b**) [33,125,235]; (**c**) [32,36,125]; (**d**) [32,116,125], with the wear ratio from [116]; (**e**) [126]; (**f**) FFF [62], continuous fibre [73] and PBF [6]; (**g**) printed PEEK against UHMWPE [143] and wear of the printed body [32].

Processes without extruded rasters need an adapted third element: in powder bed fusion, the scan (hatch) direction of the last layers, which orients melt tracks and partially fused particles on the contact face, or, for fibre-filled powders, the spreading direction that aligns the fibres [6,202]; if the scan direction rotates between layers, the triplet reduces to {contact face, layer normal}. Vat-photopolymerised layers have no in-plane direction, so the triplet is {contact face, layer normal}, the build angle setting both the layer normal and the stair-steps on inclined faces [88]; in material jetting, the print-head direction, which leaves a directional sub-micrometre texture, is the third element [35]. No powder-bed study analysed here stated the scan direction relative to sliding, so for this route, the triplet is usually incomplete (Section 11).

Orientation is reproducibly associated with a switch between interlayer-shear- and ridge-crossing-dominated wear (established as an association; the mechanism switch itself is inferred from wear morphology and orientation response; Section 18.1). Edge-on parallel sliding shears the interlayer weld along its length, giving the highest dry wear for ABS and PLA [32,125]; sliding across the edges gave the lowest wear but the highest friction, as ridges interlock and each crossing adds fracture work [32,36,116]. Friction and wear decouple: a steady-state COF varied much less with orientation than did wear [33,125,235] (Table 3). Face-on contact ranks by filler in PA12 [33,202,235]. The sign is thus established only for PLA and ABS in dry sliding and conflicting across polymers: filaments parallel to sliding gave the lowest wear for dry PEEK [140], as did parallel sliding for a PolyJet resin [35].

Re-coding also reconciles “vertical is best” [116,236] with “horizontal is best” [126,237]: Mahmood et al.’s vertical coupon [116] slides across layer edges, whereas Hanon et al.’s vertical cylinder [126] presents circumferential layer steps to a line contact, a topographic rather than interfacial effect. Signs also reverse with the wearing body: parallel sliding is worst when the printed body wears dry [32] but best when printed PEEK conditions a UHMWPE counterface in serum, its grooves channelling debris into a protective transfer film [143]. This interface-shear-versus-debris-channelling explanation is probable; a three-face × two-direction factorial on faces machined to equal Sa, measuring wear of both bodies, would test it.

Contact severity modulates these effects: the edge-on parallel/across wear ratio fell from 6.2 at 50 N to 1.15 at 100 N (derived) [116], and friction anisotropy vanished at higher load [35,115] (Section 9.2). In PBF, the orientation effect is conflicting, from counterface-dependent to negligible [84,85,86]. For fibres, end-on orientation gives the highest wear under dry, moderate-to-severe FFF contact (established), with a probable low-severity reversal in PBF (Section 7.8) [6,62,74,233]; multiscale fillers nearly halved this anisotropy (derived) [232], and it was found in one study that fibre direction outweighs layer direction (probable) [73].

### 8.2. Layer Height

Thicker layers raise the COF when sliding crosses the rasters (established; Table 3) [39,82,108,131,238,239], through rougher surfaces and groove-trapped third-body debris [141,240] (Section 9.3). The wear effect is conflicting: wear rose [122,241] or fell [242,243], and the COF fell where concentric paths ran along the track [38,132], probably because aligned ridges are not crossed and thicker layers leave fewer, hotter welds beneath deep scars. In VPP, layer thickness was not significant [166,167].

### 8.3. Infill Density, Pattern and Shell

Beneath a solid skin, infill acts non-monotonically through compliance and heat conduction (conflicting) [54,107,147,206,238,244,245]. With exposed infill or immersion, porosity governs and denser infill monotonically lowers wear (established; Table 3) [34,149,246,247,248,249]. Because infill sets the compressive response [250], sparse (15%) infill shows through the skin under a 100 N diamond cone [251]. Thicker shells lowered wear only in low-appraisal studies (insufficient) [252,253]. Pattern rankings do not transfer (conflicting) [123,128,241,254,255], except in erosion [256] and for auxetic lattices, which lowered the COF by damping friction-induced vibration [15].

### 8.4. Nozzle, Bed and Chamber Temperature, Atmosphere and Feedstock

Nozzle temperature acts through interbead healing [26,27]; a processing window is probable, its magnitude conflicting: raising it cut the SWR of PA by 75.5% [39] and it was the only significant wear factor for carbon-PLA [257], but within manufacturers’ windows, it was non-significant or secondary [38,132,241,243], and the window is material-specific [148,207]. Bed and chamber temperature evidence is insufficient (abstract-level only) [63]. The atmosphere effect is probable: printing PETG in nitrogen raised the SWR by suppressing the print-induced oxidation that thickens the transfer film [51]. Feedstock effects conflict: five nominally identical black PLA filaments differed in COF, one being an undeclared mineral-filled compound [42], whereas three PETG filaments did not [51].

### 8.5. Print Speed

Speed effects conflict even among high-appraisal designed experiments: faster printing prolonged running-in and raised the SWR of ABS without changing the steady-state COF [64], and raised the early COF [238] and PEEK friction force [258]; speed was the only significant COF factor for carbon-PLA [257], but was marginal or secondary for PLA and PEEK [139,239].

### 8.6. Powder-Bed Energy Density, Scan Strategy and Powder Refresh

In under-dense parts, higher energy density reduces porosity-driven wear (Table 3) [60]: the SWR of PA11 fell as energy density rose from 0.030 to 0.050 J/mm^2^ [137], and lack of fusion and overheating bracketed an optimum for PA1212 composites [216]. Yet the lowest energy density was best for PA12/Cu [259], at 0.42–0.47 J/mm^3^ (≈0.042–0.047 J/mm^2^ at 0.10 mm layers, derived), inside the PA11 range; this probably reflects the Cu filler (over-melting, agglomeration, voids), which measuring density, crystallinity and dispersion over a common energy range would test. Repeated scanning raised PEEK crystallinity, lowering the SWR but raising the COF, and graded parts combined both benefits [40,87] (probable, one group; confounded with porosity; Section 14.2). Powder-refresh effects are probable and material-specific [79].

### 8.7. Vat Photopolymerisation: Exposure, Post-Cure and Build Angle

Resin formulation outweighs process settings [165,173] (Section 6.2). Post-cure lowers wear (established) [92,167,171,172], but evidence that it acts through degree of conversion is insufficient (Section 10.2). Build-angle effects were mostly small and directionally inconsistent [9,167,193], and occasionally large but material-specific [181] (conflicting; Table 3), probably through stair-step topography and interlayer crack paths [88,89,156,174]. No study reviewed here isolated exposure from layer thickness or filler content.

### 8.8. Ranking of Process Parameters by Effect Size

The largest within-study effects (all derived) concerned fibre orientation (up to 7.8×), powder-bed crystallinity or density (about 10×) and photopolymer post-cure (median ≈ 2.8 × lower wear; Section 16.3), then contact-face orientation (1.15–6.2×); nozzle temperature (up to 4.1×, often not significant), layer height (about 1.5–2×), infill beneath a skin (≤1.4× in wear) and print speed (6–19% in the early COF) mattered less. As these maxima come from different studies, materials and tests, the ordering is indicative and ranks pathways (Table 3), not machine settings. Replicate scatter rivals the factor effects (up to 4.2-fold in wear [260]; 2.5–3.1-fold in the COF and linear wear [261]; derived), and statistical designs that do not measure the intermediate state [227,257,262,263,264] describe one machine–feedstock–tribometer combination, not a transferable law.

**Table 3:** Effects of AM process parameters on friction and wear (within-study comparisons; arrows give the direction for an increase in the parameter or the stated change; grades are as defined in Section 2.5). An extended version with material classes and all supporting studies is given in Table S9.

Parameter (Route)	Direction of Effect (COF; Wear)	Within-Study Magnitude	Evidence Grade	Main Pathway [Key Refs]
Contact face and layer direction vs. sliding (MEX)	COF highest edge-on across layers; wear highest edge-on parallel for PLA/ABS dry, lowest with filaments parallel for PEEK	Wear 1.15–6.2×	Mechanism switch established; sign established for PLA/ABS, conflicting across polymers	Interlayer weld shear vs. ridge interlocking [32,116,125,140]
Build orientation (PBF)	COF mostly insensitive (up to 60% lower parallel to layers); wear filler-dependent	1.48×	Conflicting	Particle-boundary cohesion, filler exposure [33,36,84]
Fibre direction vs. sliding	End-on fibres: COF and wear ↑ (FFF, moderate–severe contact); wear ↓ (PBF, low severity)	Wear 2.7–7.8×	Established (FFF, dry); reversal probable	Fibre load support vs. fibre-end fracture [6,62,73]
Layer height (MEX)	COF ↑ when sliding crosses rasters; wear ↑ or ↓	COF +40–70%; wear −38% to >2×	COF established; wear conflicting	Topography, interlayer bonding [141,238,241,242]
Layer thickness (VPP, 50–100 µm)	Wear not significant	–	Probable (null)	– [166,167]
Infill density beneath a solid skin	Non-monotonic	Wear ≤ 1.4×	Conflicting	Compliance, contact area [147,238,244]
Infill density, exposed or immersed	Wear ↓	−56 to −68% (20 → 80%)	Established	Porosity, fluid uptake [34,149,246]
Infill pattern; shell thickness	Study-specific; thicker shell wear ↓	Shell −41%	Pattern conflicting; shell insufficient	Subsurface stiffness [15,241,252]
Nozzle temperature (MEX)	Wear ↓ within a window, often not significant	Up to −75.5% SWR	Window probable; magnitude conflicting	Interbead healing [39,241,257]
Bed/chamber temperature; print atmosphere	Wear ↑ when printed in N_2_	+58%	Bed/chamber insufficient; atmosphere probable	Print-induced oxidation, transfer film [51,63]
Print speed	Running-in COF ↑; steady state ≈	6–19% (early COF)	Conflicting	Topography, thermal history [64,257]
Energy density (PBF)	Wear ↓ while under-dense	−24%; ≈10× with density	Conflicting (material-specific window)	Porosity, filler dispersion [60,137,259]
Repeated scanning (PBF)	COF ↑; wear ↓	Wear 9.6–11.8× lower; COF 2.8–3.0× higher	Probable (one group)	Crystallinity [40,87]
Powder refresh (PBF)	Wear mixed	n.r.	Probable (material-specific)	Molecular weight, crystallinity [79]
Post-cure (VPP)	Wear ↓	1.9× (none vs. 5 min)	Established	Cure state [92,167,172]
Build angle (VPP)	Inconsistent	1.10–1.43×; up to 5.2×	Conflicting	Stair steps, crack paths [9,181,193]
Feedstock brand, lot, colour	COF up to 1.50×	–	Conflicting	Additives [42,51]

MEX, material extrusion; PBF, powder bed fusion; VPP, vat photopolymerisation; n.r., not reported. Edge-on parallel/across: layer edges in the contact face, sliding parallel or perpendicular to the layer lines. Magnitudes not quoted verbatim are derived within-study comparisons and are not comparable across rows as absolute effects.

## 9. Surface Topography, Texturing and Surface Integrity

Printed surfaces differ from moulded ones less in roughness amplitude than in structure: their topography is deterministic, directional and continuous with the subsurface; the question is when it, rather than the bulk, controls friction and wear (RQ3, RQ4).

### 9.1. As-Printed Topography and Directional Friction

Each process leaves a characteristic signature (Section 3; Table 1; Figure 3): ridged FFF faces [64,65,66,116], stair-stepped vat-photopolymerised parts [88,89,156] and partially fused particles on powder-bed PA12 [80,81,82,102], modulated by build angle [83,84], as on laser-melted metals [265,266].

The lay makes friction directional. This is established for FFF [115,116,130,267,268,269]: sliding across the deposited lines raised the COF of PLA and ABS against CoCr by 2–19% [130]. It is probable for material jetting and powder-bed fusion (one study each) and reported for direct-ink writing [96,160]. Polished MJF PA12 retained COF anisotropy, an interface rather than topographic effect (Section 8.1) [36].

In Greenwood–Williamson terms [112], a ridged surface at low nominal pressure carries load on few crests, and its grooves trap third-body debris (established) [131,139,141,257]. Whether this debris instead seeds a transfer film [35,143,151] probably depends on its hardness, which no reviewed study varied.

### 9.2. Running-In, Steady State and the Load-Dependent Switch to Bulk Control

Running-in smoothing is established: Ra fell by 75–85% on PLA [66] and similarly on other FFF pins [61,64,65]. That as-printed topography governs running-in more than steady state is probable but rests on two printed systems (Section 8.5) [64,117]. Running-in is not only asperity removal: SLS PA12 bearings ran in with almost unchanged bore Ra [81], and film-forming fillers shortened it [80,203,223].

Loss of topographic control with increasing load is established. The direction effect on PolyJet plates present at 1 N vanished at 5–10 N [35], friction anisotropy on patterned PLA fell from above 60% at 27 MPa to Δµ = 0.01–0.1 at 59 MPa [115], and orientation effects on PLA friction and wear narrowed between 50 and 100 N (Section 8.1) [116,130]; low loads also favour stick–slip [66,236]. Rising pressure probably flattens the ridges until the real contact area approaches the apparent contact area, passing control to interfacial shear, interlayer bonding and the transfer film, and at higher pv to thermal softening [39,123] (Figure 6). Threshold pressures do not transfer between studies (evidence insufficient).

**Figure 6:**
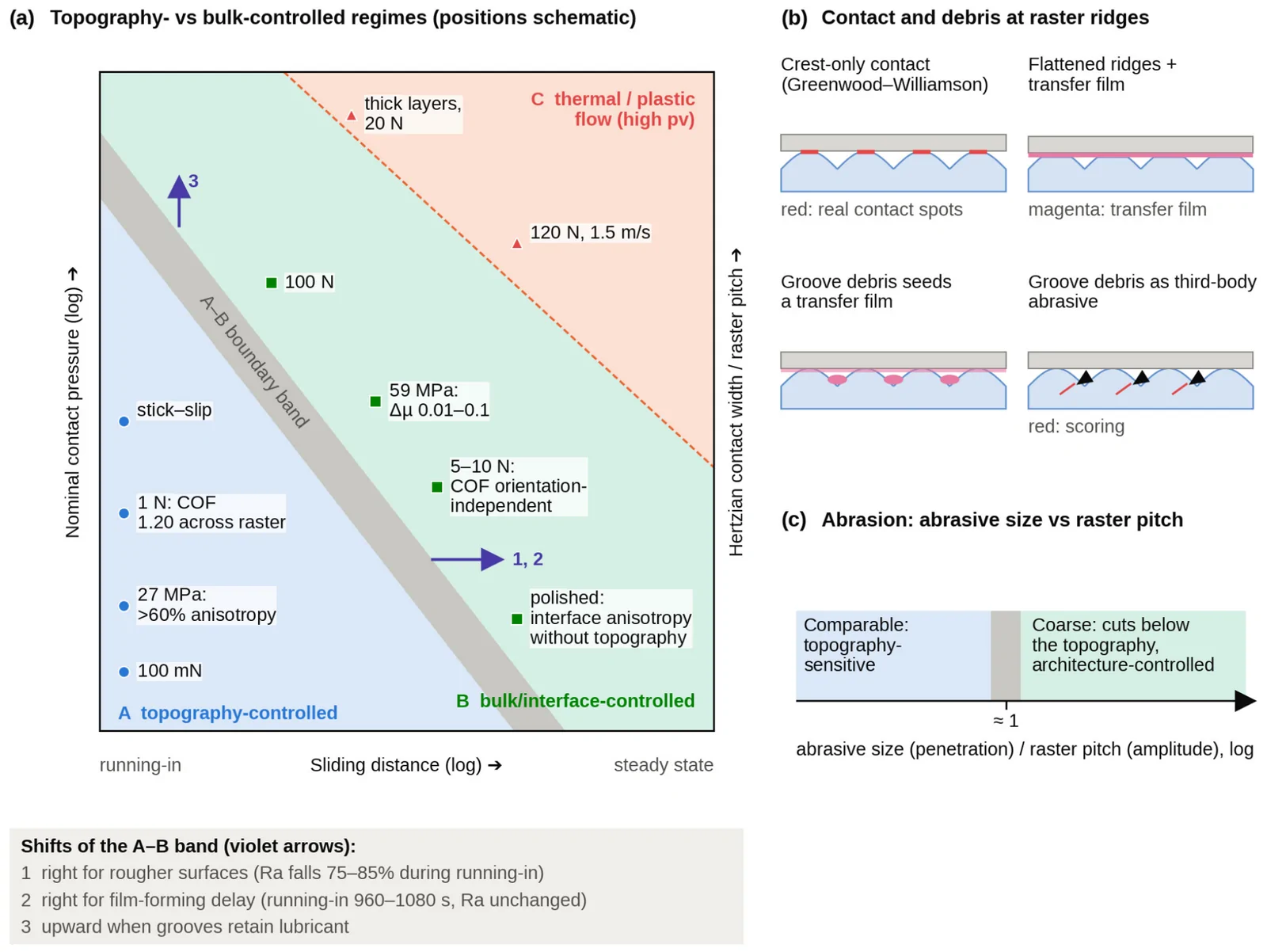
Regime diagram for topography-controlled, bulk-controlled and thermally limited friction and wear of printed polymers (**a**), with schematics of contact and debris at raster ridges (**b**) and of the abrasive-size-to-raster-pitch criterion in abrasion (**c**). Sources: (**a**) thick layers at 20 N [39]; 120 N and 1.5 m/s [123]; 100 N [116]; 59 and 27 MPa [115]; stick–slip [66]; 1 N and 5–10 N [35]; 100 mN [88]; polished specimens [36]; band shifts 1 [66], 2 [81] and 3 [54]; (**b**) [141,143]; (**c**) [50,141].

### 9.3. Beyond Ra: Spacing, Skewness, Material Ratio and Areal Metrology

Ra alone is inadequate. The lay makes it direction-dependent by up to an order of magnitude [35], and it neither separates ploughing peaks from debris-storing valleys nor carries spacing information. Ssk and Sv correlated with the COF (r = 0.91–0.99), whereas Sa did not [116], and material-ratio and spacing parameters tracked wear and running-in better than Ra [37,81,244]. Amplitude and response often decouple: vapour smoothing raised Ra without changing the COF [102] and minimum wear coincided with maximum Ra [132,242]. Areal ISO 25178 parameters, standard in metal AM [266,270], are rare in the polymer studies [64,85,116,131,217,271] (Section 11).

Roughness probably predicts abrasive wear only when abrasive penetration is comparable to raster amplitude and pitch. In two-body abrasion of PEEK, thicker layers raised Sz and, from 0.25 mm, sharply raised wear, but roughness co-varied with hardness and interlayer weakness [141,240]; in coarse three-body abrasion, volume loss correlated less with Sa for printed than for extruded plates (Section 14.1) [50]. Abrading re-faced specimens of fixed layer thickness with grits spanning the raster pitch would resolve this.

### 9.4. Designed Textures and Multi-Material Patterned Surfaces

An intermediate texture optimum recurs [124,272,273]: UHMWPE wedge textures in serum improved up to 200 µm depth but spalled by fatigue at 250 µm (Section 13) [150], and TPU textures cut the water-lubricated COF by 63–64% only at intermediate area density [274]. Concave, debris-storing features cut MultiJet photopolymer wear volume by 94–97% (derived), whereas convex columns crumbled [275]. Printed pits, bumps and stream-facing prisms likewise reduced soil abrasion and erosion (Section 17) [276,277,278].

Benefits are regime-dependent. Textures reduced dry but increased wet wear of short-carbon-fibre nylon [59], raised the COF in short dry PLA tests [239], and destabilised friction on circular but not linear tracks [279,280]. They probably help where they manage debris (dry) or generate lift (lubricated) and hurt where they expose more area to water or misalign with sliding; Stribeck mapping at fixed texture orientation would test this. Multi-material grading is probably beneficial: rigid-over-soft laminates lowered the COF and SWR by 25.7% and 62.6% [94], although bronze-filled PLA strips gave irreconcilable wear rates [16] and bi-colour patterns changed only surface plastic flow [123].

### 9.5. Soft Contacts: Skin, Rubber, Elastomers and Ice

Against compliant counterbodies, friction is governed by viscoelastic hysteresis and multi-scale conformity [114], and AM mainly tailors macro-texture, from substrates that validated Persson’s theory [106] and finger-pad textures [281] to bio-inspired, gripping, adaptive and elastomer surfaces [14,68,95,96,107,108,160,163,282] and fibres raising TPU friction on ice [17] (Section 17). Such contacts need power spectra or deterministic feature descriptors, not hard-contact amplitude parameters.

*Practical implications and key findings.* Three design rules follow, each with a limited domain. First, for as-printed FFF faces sliding against metal at low contact pressure and during running-in, the lay matters: friction-critical faces should be built with the lay parallel to sliding for low friction and across it for grip [115,130], or refinished; at higher pressure, the bulk and interlayer interfaces take over and refinishing gains little (Section 9.2), and the preferred direction can reverse for other polymers or counterbodies (Section 8.1). Second, grooves trap debris, acting as third-body sources in dry sliding unless the debris is soft enough to form a film; under lubrication, concave textures of intermediate depth and area density reduced friction and wear in the systems tested, whereas excessive depth invited fatigue spalling [150,274,275]. Third, Ra alone neither predicts nor specifies tribological behaviour: skewness, valley depth and feature spacing relative to contact width should be specified and reported (Section 11.4).

## 10. Post-Processing and Surface Engineering

Post-processing usually changes the surface (roughness, extrusion skin, chemistry) and bulk (crystallinity, degree of conversion, porosity, interlayer bonding) together. Of the 23 entries in Table 4 (Figure 7), 12 act mainly through the bulk, 4 through roughness or texture and 2 through both, and 5 remain unclear (derived).

### 10.1. Annealing and Heat Treatment

Annealing reduces dry-sliding wear of FFF PEEK against steel (established, but conditional). The SWR fell by 34–72% (derived) as crystallinity rose (Table 4) [41,138,139], with similar trends for carbon-fibre PEEK, PA6 composites and PETG [63,283,284,285]. The benefit vanished at 50–70 N [139], held only for filaments parallel to sliding [140] and, in water, depended on deposition strategy [99]; the COF often rose [51,138].

What these studies measured supports a bulk pathway: crystallinity in all the PEEK studies, density and interlayer (90°) tensile strength in one [138], and a common finish of annealed and unannealed pins in two PEEK studies [99,140] and one PETG study [51]; the role of a more continuous, groove-filling transfer film is inferred from SEM of worn pins and counterfaces [41,140]. The bulk pathway is therefore probable rather than established: post-finish roughness was not reported, and crystallinity and interlayer strength rose together, so their contributions are inseparable.

For PLA, the results conflict, and the two key studies differ in what they measured as much as in outcome. Annealing at 95 °C raised the SWR by 83% (solid specimens) in long, mild sliding against smooth steel [41]; tensile and compressive strength rose, crystallinity was not reported, and embrittlement was inferred from powder-like debris and an unstable transfer film. Annealing at 110 °C cut wear by 84.8% (derived) [127] while crystallinity rose from 19% to 41–46%, porosity fell from 4.6% to 2.4–3.4% and Ra fell from 2.14 to 0.75 µm, all measured but co-varying, under unreported test conditions; longer anneals raised wear again, with micro-cracking on worn surfaces. Annealing also helped in short abrasion and in date-pit/PLA [246,286,287], where crystallinity and roughness were not measured. No annealing study measured thermal or hydrolytic degradation (molecular weight), so its contribution remains untested. The likeliest explanation (probable) is that densification and smoothing reduce ploughing in short or abrasive tests, whereas lost ductility destabilises transfer films in long, mild sliding; replicate scatter can mask both [260]. A 2 × 2 design crossing annealing with refinishing to a common Sa, measuring crystallinity, interlayer strength and molecular weight (Figure 7), would separate roughness, bulk and degradation contributions; no such study was identified.

### 10.2. Post-Curing of Photopolymers

Post-cure lowers wear (established; Table 4) [92,167,172], and, beyond a minimum, cure time matters little [120,167,171]. Evidence that it acts through degree of conversion is insufficient: none of these studies measured conversion [92,120,167,172], and measured conversion did not rank the wear of printed crown resins [173].

### 10.3. Polishing, Vapour Smoothing and Laser Polishing

Polishing lowered the wear and COF of photopolymers (Table 4) [88,166], as did glazing [191], probably through roughness alone. Against smooth steel, polishing thermoplastics probably raises friction: the COF of FFF PA rose about tenfold between different specimen series (derived; Table 4) [39], and SLS PA12 behaved likewise [135]. A larger real contact area probably helps where ploughing dominates and hurts where friction is adhesive, and polishing can expose pores [99]. Vapour smoothing was probably neutral [102]; evidence for laser polishing is insufficient [288].

### 10.4. Coatings and In Situ Surface Layers

Hard ceramic-filled coats on MJF PA12 raised the COF by 3.2–3.7× (derived; wear unmeasured) [102]. Low-shear layers act through transfer films [23] but only in narrow windows of load, speed or environment [248,289,290]. Coating adhesion to layered substrates remains unexamined.

### 10.5. Laser Texturing

Femtosecond-laser grooves on carbon-fibre PEEK in water lowered the SWR with fibres parallel to sliding but raised it with fibres perpendicular to sliding (probable, one study; Table 4) [291].

### 10.6. Impregnation, Infiltration and Pressure Consolidation

Pressure consolidation acts on porosity: cold and hot isostatic pressing lowered L-PBF UHMWPE wear about tenfold, to that of extruded material [60], and compression moulding lowered continuous-fibre PA6 wear with fibres normal to the surface [73] (Table 4). Filled pores can act as reservoirs (Section 13.5) [56]: stored oil lowered the high-temperature COF of porous CNT/PEEK (wear not measured) [55], and PEEK infiltration reduced the mass loss of Ti6Al4V lattices relative to cast/forged alloy [12].

**Table 4:** Effect of post-processing on the tribological performance of printed polymers.

Treatment	Polymer/Process	Change in Roughness	Change in Bulk State	Effect on COF	Effect on Wear	Acts Mainly via	Ref.
Annealing 150–250 °C	PEEK/FFF, dry vs. steel	n.r. (as-printed Ra ≤ 3.91 µm)	Xc 11.98 → 26.31% (150 → 190 °C); density 1.250 → 1.270 g/cm^3^; UTS ↑	0.37 → 0.24 at 90 N; rises with annealing T at 30 N	SWR −34% (30 N); ≈−59% (90 N); none at 50–70 N	bulk	[138,139]
Annealing 160–300 °C	PEEK/FFF, block-on-ring	all pins pre-worn to ring curvature (P400) after treatment; Sa n.r.	Xc 15.84 → 27.89% (200 °C), 33.99% (250 °C)	↑ (parallel), ↓ (vertical, 45°) at 160 °C	min 1.37 × 10^−6^ mm^3^/(N·m) (200 °C, parallel); slight ↑ vertical/45°	bulk	[140]
Multi-step annealing to 220 °C	PEEK/FFF	n.r.	Xc 21.8 → 24.0%; ductility ↑	running-in ≈200 → ≈50 min	SWR −72% (solid), −81% (re-entrant)	bulk	[41]
Annealing 200–300 °C	PEEK, CF-PEEK/FFF, water	all polished after treatment to Ra < 0.5 µm	Xc ≈ 24 → ≈28%	CF-PEEK below PEEK	optimum T depends on deposition strategy; neat PEEK mostly ↑	bulk	[99]
Annealing 100 °C, 220 kPa	PETG/FFF	all pins polished (600 grit) after treatment; finish n.m.	n.m. (XPS O 18 → 17 at.%)	↑	SWR 3.4 → 1.5 × 10^−5^ mm^3^/(N·m) (−56%)	bulk	[51]
Annealing (qualitative)	CF-PEEK, PA6/GF/CF/FFF	n.r.	Xc, hardness, interlayer bonding ↑ (claimed)	n.r.	generally ↓	bulk	[63,283,284,285]
Annealing 95 °C, 1 h	PLA/FFF, 5 N vs. steel, 600–900 min	n.r.	strength ≈ +10%; ductility ↓	earlier, stronger fluctuations	SWR +83% (solid); ≈19× (re-entrant)	bulk (embrittlement)	[41]
Annealing 90–130 °C, 60–180 min	PLA/FFF, test conditions n.r.	Ra 2.14 → 0.75 µm (110 °C, 60 min)	Xc 19 → 41%; porosity 4.6 → 2.8%	0.47 → 0.16	7.52 → 1.14 × 10^−3^ mm^3^/(N·m) (−85%); longer anneals ↑	both	[127]
Annealing 70–90 °C	PLA, PLA-wood/FFF, SiC paper	n.m.	n.m.	n.r.	volume −20% (PLA), −10% (PLA-wood); PLA gain lost after immersion	unclear	[246]
UV post-cure (presence, device)	methacrylate C&B/DLP, chewing	n.m.	DC n.m.	n.m.	none vs. 5 min LED: +93%; time n.s.	bulk	[167]
N_2_-atmosphere post-cure	splint resins/DLP, LCD	polished	HV slightly ↑ (one printer)	n.m.	nano-wear depth ↓ (all); micro-wear ↓ (one printer)	bulk	[92]
Post-cure time	MSLA resins; DLP resin	n.r.	hardness ↑	n.s.	n.s. (1.22% contribution); −22% (20 vs. 7 min)	unclear	[120,171]
Steam 121 °C, 2 bar, 90 min	denture-tooth resin/DLP	polished	HV +31%	n.m.	depth −51%; median volume −80%	bulk	[172]
Mechanical polishing	splint resin/DLP, chewing	reduced (n.m.)	unchanged	n.m.	volume −38% (*p* = 0.003)	roughness	[166]
Mechanical polishing	PU-acrylate/DLP	Ra 5.57–10.26 → 0.10–0.18 µm (15–75°)	unchanged	↓ at all angles; 0.433 (75°, 200 mN)	lowest at 75° polished	roughness	[88]
Polishing (skin removal)	PA/FFF; PA12/SLS	skin removed (n.m.)	unchanged	0.05–0.06 → 0.50–0.60 (FFF; different specimen series); ↑ (SLS)	n.m. on matched specimens	unclear	[39,135]
Laser polishing	ASA/FFF	↓	hardness ↑	n.r.	↓	both	[288]
Vapour smoothing	PA12-GB/MJF	Ra 9.70 → 13.07 µm	n.m.	0.131 → 0.125	n.m.	roughness (no effect)	[102]
Hard coats (SiO_2_-, ceramic-filled epoxy)	PA12-GB/MJF	Ra 1.18 and 35.57 µm	Shore D 75 → 84	0.131 → 0.413 and 0.479	n.m.	unclear (new surface)	[102]
In situ ZIF-8 after sulfonation	PEEK/FFF, polished	n.r.	surface chemistry	n.r. numerically	SWR 1.091 × 10^−5^ → 4.68 × 10^−6^ mm^3^/(N·m)	unclear (surface layer)	[248]
Femtosecond-laser texturing	CF-PEEK/FFF, water	grooves 9–27 µm deep	—	−7.8 to −16.9% (fibres ∥)	up to −55% (∥); +45 to +120% (⟂)	roughness (texture)	[291]
Heat + CIP + HIP	UHMWPE/L-PBF	n.m. before test	relative density ≈ 61 → ≈90%	n.m.	≈10× lower; equal to extruded (*p* = 0.60)	bulk	[60]
Compression moulding	continuous CF/PA6/FFF	n.r.	≈10% voids removed	↑ (fibres in-plane)	−46% (fibres normal, 2 MPa)	bulk	[73]
Oil impregnation	TPMS resin lattices/VPP	sanded	oil content 55–88%	0.02–0.06	wear depth −50 to −70% vs. dry	bulk (pore reservoir)	[56]

Xc, crystallinity; DC, degree of conversion; HV, Vickers hardness; n.r., not reported; n.m., not measured; n.s., not significant; ∥/⟂, fibres parallel/perpendicular to sliding. Percentage and fold changes are derived from the cited full-text values unless reported directly. In the three studies that gave all specimens one finish after annealing, differences are attributed to the bulk, but post-finish roughness was not reported or was given only as a bound, and no as-printed-face control was included. Pathway counts in the text exclude the qualitative row.

**Figure 7:**
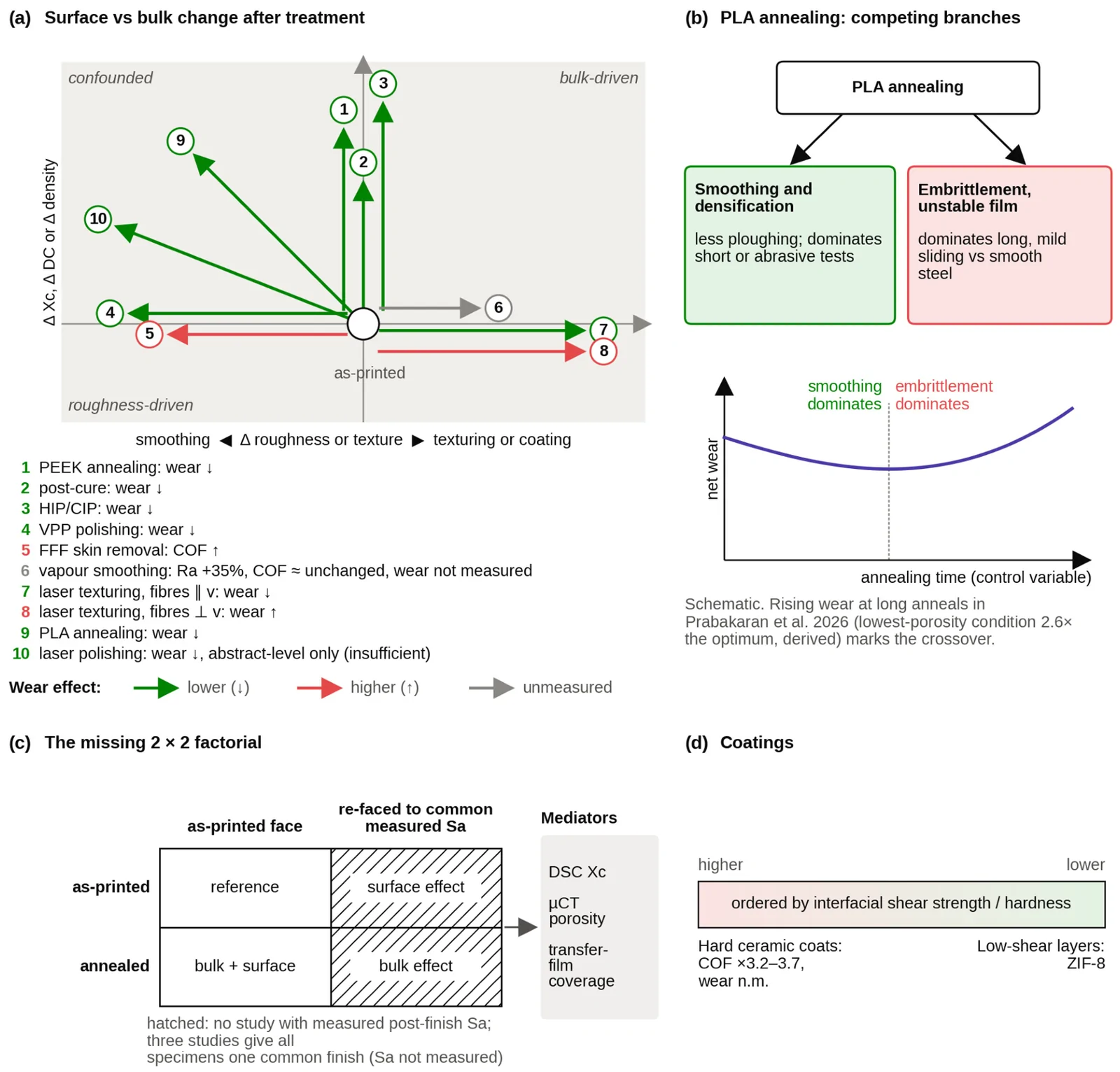
Post-processing pathways for printed polymers: roughness-driven, bulk-driven and confounded effects on friction and wear. Sources: (**a**) 1 [41,138,139,140], 2 [167,172], 3 [60], 4 [88,166], 5 [39], 6 [102], 7 and 8 [291], 9 [127] and 10 [288]; (**b**) smoothing and densification [127,246], embrittlement [41] and the crossover [127]; (**c**) studies applying one common finish [51,99,140]; (**d**) hard ceramic coatings [102] and ZIF-8 layers [248].

## 11. Tribological Test Methods and Reporting Practice

This section audits reporting in the 245 full-text experimental studies analysed and derives a minimum reporting set (Table 5) that would make tribological data on printed polymers transferable. The percentages describe the present evidence base only (appraisal classes: Section 2.4) and explain why absolute results rarely transfer (Section 16).

### 11.1. Configurations Used

Pin-on-disc (29.0%), reciprocating sliding (24.1%), ball-on-disc (12.7%) and dental chewing simulators (12.2%) dominate; ring, bearing, thrust, abrasion, gear and erosion rigs together account for 17.6% (derived), the abrasion and erosion rigs often being purpose-built devices whose requirements are still being defined [292]. A third of studies cite a test standard (Table 5), 57 of them ASTM G99, and 14 run sliding rigs against abrasive paper (two-body abrasion, not sliding wear). Reciprocation reverses the shear relative to the rasters at every stroke and alters polymer wear [110]; for the same printed dental resins, ball-on-disc, block-on-ring and reciprocating results correlated weakly or negatively [182]. Which body is printed also matters (a printed pin presents one face to a continuous track, a printed disc its rasters to repeated asperity passes), and printed–printed pairs [4,101,293] are not interchangeable with polymer-on-steel data.

### 11.2. Reporting Completeness of Published Studies

Counterface material, normal load, sliding speed or frequency and sliding distance are each reported in 82–91% of studies, and all four in 68.6%. Contact-state quantities are rarer: contact pressure, counterface hardness and roughness, specimen roughness and relative humidity (although PLA, polyamides and photopolymers absorb water) each appear in under a third (Table 5). Only 18.4% discuss running-in or steady state, although raster ridges are levelled during running-in [66,117,121]. The orientation triplet (Section 8) is fully stated in 17.6% and cannot be reconstructed in 46.5%. Fewer than half report dispersion, and a third a statistical test. Only three studies (1.2%) report all eight core contact and statistics items [53,89,142]; full nine-item compliance was not screened. Crystallinity or degree of conversion was measured in 18.0%, yet nominally identical black PLA filaments printed identically gave a mean COF of 0.246–0.368 [42].

### 11.3. Wear Metrics

Thirty-three studies report mass alone, 20 no wear quantity and under a quarter an author-reported specific wear rate in mm^3^/(N·m) (Table 5). Mass is misleading for porous, fluid-absorbing polymers exchanging material with the counterface: printed PLA and eroded Nylon/CF gained mass by transfer or embedded alumina [293,294], and for SiC/PA12, mass loss fell by 53.8% but the specific wear rate by only 20.2% (derived) [135]. Depth-based dental metrics conflate creep with material removal [10,196]. Fourteen studies use eight non-standard units, among them mm^3^/(N·mm), 10^3^ times mm^3^/(N·m) [88,136,236,249,295], g/(N·m) [296,297], mg/s [124,298], µm^3^/(N·m) [228], m^2^/N [54] and µm/cycle values implying per 1000 cycles [168]. Twenty-four contain implausible values and a twenty-fifth contains irreconcilable ones [16]: losses exceeding the specimen or ≈23% of it in a 50 N chewing test (derived) [195,249,254,296,298,299], losses below balance resolution [124,229] and standard deviations exceeding means [165,189]. Only seven studies quantified counterface wear (enamel or ceramic [10,178,185,186]; steel or CoCr [13,66,89]).

### 11.4. Minimum Reporting Set

The nine items of Table 5 allow test severity (nominal pressure and pv) and specific wear rate to be reconstructed and a test to be placed on the mechanism map (Figure 8); Hertzian pressure also needs both bodies’ elastic modulus and Poisson’s ratio, and an Archard coefficient needs the hardness of the wearing body (item 9). Closing the largest gaps needs only a profilometer, a sketch of the triplet, a stated steady-state criterion and DSC for semicrystalline thermoplastics or a conversion measurement (FTIR or Raman) for photopolymers.

## 12. Wear Mechanisms in Layered Polymer Architectures

Adhesion with transfer, abrasion, fatigue and delamination all operate in printed polymers [20,22,119], but layering changes where damage starts. Mechanism evidence is thin: 74 of the 245 studies analysed use SEM alone, 61 name no worn-surface microscopy, 11 image the subsurface, and 28 of 66 invoking a tribofilm analysed it chemically. Figure 8 maps and grades the dominant mechanisms.

### 12.1. Adhesion and Transfer Films Modified by Printing

Transfer-film control of mild wear [23,109] is established for printed thermoplastics, and printing modifies it: print-induced oxidation of PETG thickened the film (0.57 vs. 0.22 µm) and lowered wear (Section 5) [51]. Crystallinity modifies the film in PEEK composites (probable; Section 14.2) [40,87], annealing can stabilise or destabilise it (Section 10) [41,138,139,140], and raster grooves seed it [143,272]. Oxidative, tribochemical wear is established, with oxidation products identified spectroscopically in five studies from three groups [53,136,142,223,300].

### 12.2. Interlayer Shear, Raster Peeling and Delamination

Interlayer weld failure, the mechanism most specific to material extrusion, is established: ABS and SLS PA12 wore most with layers parallel to sliding (Section 8) [32,33], and pore-initiated separation, raster pull-out and strip-like debris corroborate it [4,50,99,116,122,127,128], although only four of the 11 studies with subsurface imaging sectioned worn printed layers [32,85,127,141]. Photopolymers show analogous interlayer cracking under chewing loads [165,174,185,188,189].

### 12.3. Groove-Trapped Three-Body Abrasion

Debris trapped in raster valleys acts as a third body (established): coarser PEEK layers trapped debris and sharply raised abrasive wear (Section 9.3) [141,240], and debris raised the COF or formed wedges in PEEK, CF-PLA and ASA [131,139,257]. Whether debris protects or abrades probably depends on its hardness, soft debris compacting into films [143,151]; no study reviewed here varied debris hardness at fixed groove geometry.

### 12.4. Thermal Softening, Stick–Slip and Friction-Induced Vibration

Poor heat dissipation causes softening: PLA bushes deformed (up to 81.0 °C) [124] and PA6 and nylon gears ran above Tg or failed thermally [4,101,121], whereas substrate conductivity and nano-SiO_2_ delayed softening in PEEK [223,301]. Stick–slip, mostly inferred from COF traces, varies with raster direction, layer thickness, load and speed [32,33,66,236,239,267] and with test-system stiffness [302], which should therefore be reported. Re-entrant lattices damped vibration [15].

### 12.5. Fibre Breakage, Pull-Out and Debonding; Particle Debonding

In dry, moderate-to-severe contact, end-on fibres in FFF composites fragment into third bodies and wear more than parallel fibres (established; Section 7.8) [62,73,233], whereas PBF CF/PEEK at low severity shows the reverse [6]. Debonding, pull-out, film disruption and counterface abrasion [53,99,198,202,206,207,303] are consistent with unaged 10 wt% GF-PLA wearing ≈10× more than neat PLA (derived) [58]. Particles act similarly through depletion and pull-out (Section 7) [13,135]; Al_2_O_3_ fragments raised the COF by 249% at 10 N but lowered it by 10% at 100 N once re-embedded [218].

### 12.6. Fatigue and Subsurface Cracking in Cyclic Contacts

Subsurface damage under cyclic contact is established: printed resins showed fatigue cracks and delamination, dental composites cracking more than a milled block (optical coherence tomography) [93,168,173,185], gear roots fractured at poorly sintered layers [4,101] and unannealed PEEK cracked by fatigue at high load [138,199,300]; only one cyclic-contact study reviewed here imaged the subsurface.

### 12.7. Regime Transitions with Load, Speed and Temperature

The COF commonly falls with load [81,84,128,223], consistent with sub-linear growth of the real contact area [112]. Wear rose with load in TPU (+125%, derived) [146] and PEEK [73,138] but fell in composites forming compact films [142,223], and filler rankings crossed over [198,199,218]. Speed effects conflict: the COF rose with speed in SLS polyamides and CNT/HDPE [84,304] but TPU wear and ABS kinetic friction fell [146,267], and in gears, PA6 wear rose above Tg while PEI wear fell [121]. The sign is probably set by contact temperature relative to Tg and the loss peak [114], testable by speed sweeps at constant pv with measured flash temperature; heating alone from 20 to 200 °C raised the COF of hBN/PEEK [103]. No study reviewed here constructs a wear-mechanism map [305].

**Figure 8:**
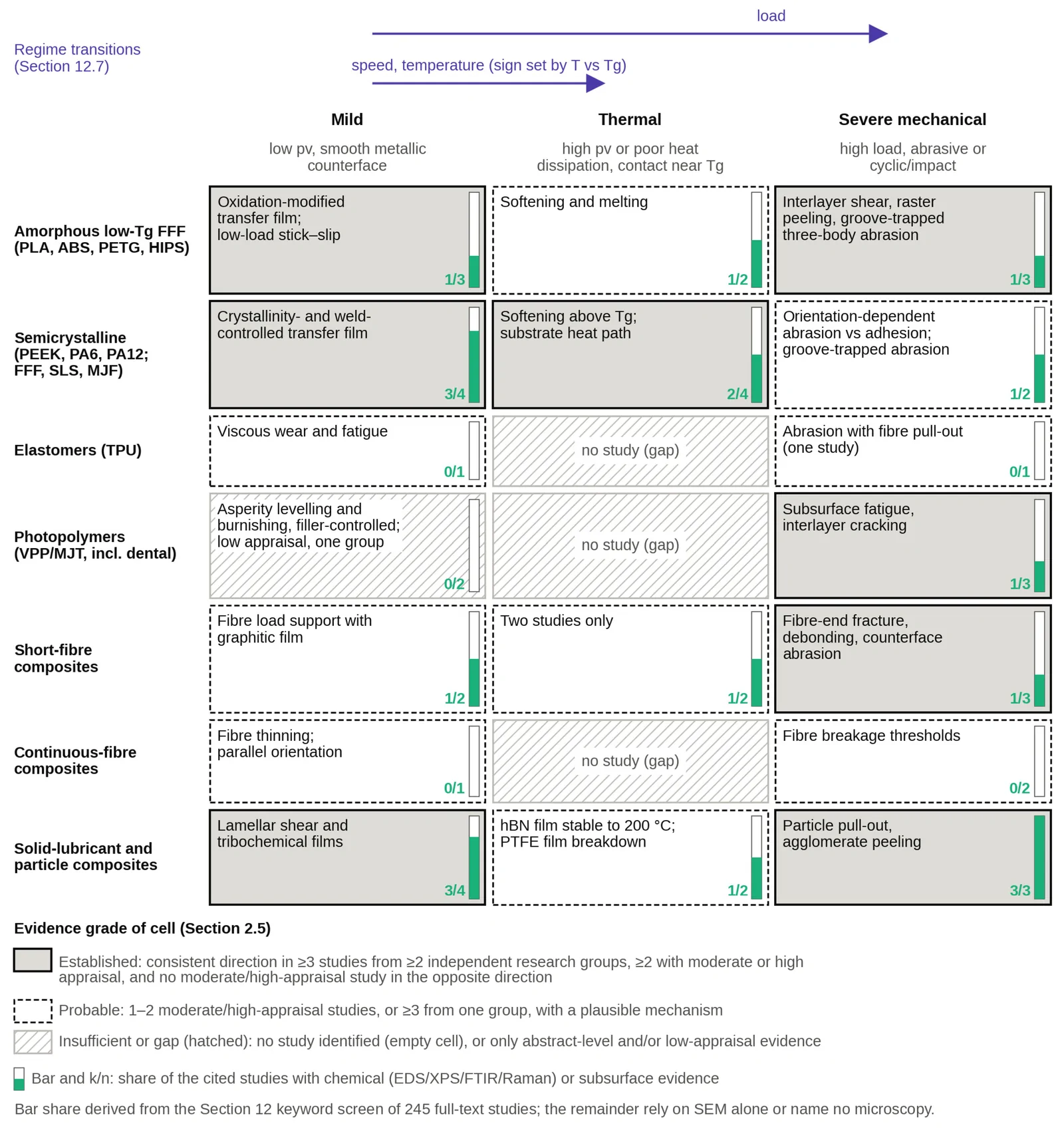
Mechanism map for printed polymers and composites: dominant friction and wear mechanisms by material class and contact severity, with the evidence grade of each cell (established: consistent direction in ≥3 studies from ≥2 independent research groups, at least two with moderate or high appraisal, no moderate/high-appraisal study opposed; probable: 1–2 moderate/high-appraisal studies, or ≥3 from one group, with a plausible mechanism; insufficient/gap: no study identified, or only abstract-level and/or low-appraisal evidence, hatched). Studies supporting each cell (mild; thermal; severe mechanical): amorphous low-Tg FFF [15,32,50,51,66,122,124,267]; semicrystalline [4,33,34,40,101,121,138,140,141,301]; elastomers [146]; no study; [17]; photopolymers [171,189]; no study; [93,174,185]; short-fibre composites [4,53,58,206,232,235,301]; continuous-fibre composites [73]; no study; [74,233]; solid-lubricant and particle composites [13,103,134,135,136,199,218,300].

### 12.8. Synthesis: Load, Speed, Temperature and Debris

Section 12.1, Section 12.2, Section 12.3, Section 12.4, Section 12.5, Section 12.6 and Section 12.7 describe one sequence for printed thermoplastics and composites (Figure 8). At low load and speed, the printed lay governs friction and grooves collect debris; wear is mild and controlled by transfer films, which print-induced chemistry, crystallinity and soft fillers modify. As load rises, ridges flatten and the interlayer weld becomes the weakest shear path: wear of parts with weak welds or exposed fibre ends rises, and debris changes from fine, film-forming particles to third-body strips, fibre fragments and pulled-out particles, a transition that load-bearing fillers and compact films delay. As speed or pv rises, the contact approaches Tg (≈60 °C for PLA) and softening, film breakdown and thermal failure take over; the sign of the speed effect depends on contact temperature relative to Tg. Cyclic contact moves damage below the surface as fatigue cracks along layers. The mild-wear and fatigue end-members are established; the load- and temperature-dependent transitions between them are probable, as no study has mapped them for a printed polymer.

## 13. Lubricated and Environmental Conditions

Fluids and ageing can reorder dry-sliding rankings (Section 5, Section 6 and Section 7), acting on grooves, pores and fibre–matrix interfaces differently from the bulk.

### 13.1. Oil and Grease

Oil and grease lower the friction of printed polymers (established) [8,54,61,272]: for ABS, PC and PLA, the COF fell from 0.11–0.16 dry to ≈0.1 in grease and ≈0.05 in oil [54], palm oil reduced SLA wear in all build orientations [156], and CF/PEEK grades converged to a COF of 0.06–0.07 in oil [8]. One protic ionic liquid, however, thickened in the contact, raising the PA12-composite COF to 0.25 and wear 4.9–9.0-fold, whereas another lowered the COF by 68% (derived) [306]. Where identified, the regime was boundary or early mixed (λ ≤ 0.06) [8,49,61]; full-film and printed-versus-conventional data are lacking (insufficient; Section 15.2).

### 13.2. Water and Seawater

Where the matrix carries the load, water decouples friction from wear: it lowered the printed neat PEEK COF to 0.19–0.21 but raised its the SWR about tenfold [53], and acted likewise on nylon and short-CF nylon at 20 N, although SCF-nylon wore less wet than dry at 40 N [59]. CF/PEEK tolerated or benefited from water [53,142], yet printed PEEK grades wore more than moulded ones (probable; one group; Section 15.2); better water tolerance of continuous than short fibres is insufficiently evidenced [208,209]. Water effects are thus conflicting, plausibly via matrix uptake and fibre architecture; varying fibre length at fixed matrix and moisture would separate the two. Seawater data are scarce [79,247]; PLA-on-PLA sliding in water caused little hydrolysis [293].

### 13.3. Biological Fluids

Artificial saliva lowered the COF and SWR of printed and extruded PEEK alike [307] (dental resins: Section 6.2). In serum, printed grooves trapped UHMWPE debris rather than supporting a fluid film [143], oriented short CF lowered the COF of PEEK [203], and wedge textures lowered UHMWPE wear by 25.66% at 200 µm depth, rebounding at 250 µm [150]. The benefit is load- and filler-limited: the SWR of printed PEEK rose ≈15-fold from 14 to 70 N [49], and Si_3_N_4_ fillers raised the COF and damaged CoCr in saline [13].

### 13.4. Hygrothermal Ageing, Thermal Cycling and UV

Ageing effects are conflicting. In pressurised water, GF-PLA absorbed 1.9–3.5 times more water than neat PLA and lost its unaged 23–32% wear-depth advantage within 28 d (derived) [57]. Thermal–hygrometric cycling effects were non-monotonic and load-dependent, reversing sign between 200 and 400 cycles in neat and 10 wt% GF-PLA [58]. Saliva raised the SWR of four of five printed dental resins up to ≈14-fold (derived) [308], and immersion, UV and atomic-oxygen effects were material-specific [246,289,309]. Outside dentistry, no study reviewed here combined ageing with fatigue loading.

### 13.5. Porosity: Lubricant Reservoir or Ingress Path?

Two kinds of porosity must be distinguished: designed, interconnected porosity deliberately impregnated with lubricant, and process-induced porosity (inter-bead voids, unfused powder) that is not. Oil-impregnated TPMS lattices cut wear depth by 50–70% versus dry sliding (COF 0.02–0.06; optimum design porosity 25–35%) [56], and oil-carrying fillers and leached porous PEEK rely on the same release (Section 7.3) [55,154,157]. Storage in as-printed voids, pores and grooves was reported only qualitatively or, in abstracts, its wear contribution unisolated [7,8,52,54,295]. Outside AM, frequency-dependent fluid reabsorption in imbibed foams [310], squeeze-flow expulsion [311] and Kozeny–Carman permeability, valid only over limited porosity ranges [312], have been described but not validated on printed lattices.

In water, the same features become ingress paths: printed PEEK absorbed 0.78% water versus 0.3% for moulded [53], GF-PLA lost its wear advantage with uptake (Section 13.4) [57], textures enlarging the wetted area raised the wet SWR [59] and culture medium degraded 15–infill PLA far more than 90–infill PLA [249]. In dry contact, open porosity lowered load support wherever it was varied (Section 8.3) [34,60]. PLA cycling data [58] do not test ingress, porosity being constant.

We therefore propose (Figure 9) that porosity acts as a reservoir only when a lubricant, supplied once and retained by viscosity and capillarity, neither plasticises, hydrolyses nor debonds the matrix and its interfaces, and fills surface-open pores isolated from load-bearing welds at a porosity that preserves load support. This benefit is probable for designed or impregnated porosity but insufficiently evidenced for as-printed porosity, whose lubricant retention and Stribeck response no study reviewed here reported; crossing CT-measured porosity with water, PAO and grease on PEEK, PA and PLA, with Stribeck curves, uptake and post-test sections, would resolve it.

## 14. Mechanical Properties and Tribological Performance

### 14.1. Hardness and Modulus as Predictors

Hardness alone does not transferably predict wear across printed polymer systems and wear regimes (established). In three-body abrasion, microhardness correlated with volume loss for extruded sheets (r = 0.62) but not for printed specimens (r = 0.08), which lost whole deposited paths [50]; softer printed materials wore up to ≈5 times less than harder ones (derived) [65,104]; and neither hardness nor H^2^/E ranked resin, ABS, PA6.6-composite or TPU grades [10,120,128,148,204,308].

The apparent counter-example, r = −0.93 across ten occlusal-device materials [11], spans two mechanism clusters (flexible printed resins with fatigue or adhesive scars; abraded rigid materials), with r = +0.59 within the rigid cluster (n = 5, derived); printed-versus-milled and density-series correlations are similarly confounded [60,171]. Anisotropy compounds this, friction varying with sliding direction at near-constant H/E [36], and H/E or H^3^/E^2^ was related to wear only once [308]. Polymer indentation hardness is also rate- and environment-dependent [313], so a single value omits the viscoelastic losses that govern deformation friction.

The conclusion rests on moderate- or high-appraisal studies in three polymer classes and two wear modes, sliding and chewing simulation: in unfilled thermoplastics, softer printed polymers wore less in dry sliding [65] and hardness did not rank ABS pins [128]; nor did it rank filled PA6.6 composites in sliding [204] or occlusal photopolymer resins in chewing simulation [10,308]. For elastomers and three-body abrasion, the evidence is low-appraisal only [50,148]. The common reason is that the harder material changed the wear mode (from deformation and transfer to fracture, fragment release or removal of whole paths), so hardness ranks wear only within one mechanism, as Archard’s law assumes (Section 14.4).

### 14.2. Crystallinity and Degree of Conversion

Higher crystallinity probably lowers wear but raises friction (one group): in solid-lubricant-filled LPBF PEEK, raising crystallinity from ≈35% to ≈44% lowered the SWR 9.6–11.8-fold and raised COF 2.8–3.0-fold (derived), as the compliant low-crystallinity matrix formed a more continuous transfer film [40,87,109]. Porosity (2.5–13.3%) co-varied, as did density in annealed PEEK [138], and annealing lowered wear even in amorphous PETG (Section 10.1) [51]. Degree of conversion is an insufficiently evidenced wear descriptor: photopolymer wear followed filler content, not wash and cure [120,173], and no study reviewed here measured conversion with post-cure and wear.

### 14.3. Interlayer Strength, Toughness and Debris Morphology

Interlayer strength as the controlling descriptor remains probable because stronger interfaces were confounded: CF grafting raised the Z-direction strength of fused-granulate PA6 by 66.8% but cut porosity from 4.85% to 1.90% [76,100], and thermal treatments changed bonding, crystallinity and density together [138,285]. Toughness acts through debris morphology (embrittled PLA shed powder-like debris, toughened PEEK wore less; PLA annealing: conflicting; Section 10.1) [41,127] but is unnecessary where films form: graphite cut PLA impact strength by 47% yet lowered wear ≈3-fold, and solid-lubricant resins wore 92% less despite lower hardness and strength [91,211].

### 14.4. Archard and Lancaster–Ratner Relations for Printed Parts

Archard’s coefficient K [113] is not constant for printed parts: it fell as porosity closed in PBF UHMWPE (wear ≈10-fold lower for ≈3-fold higher hardness) [60], shifted with regime in serum-lubricated PEEK and neat PPS [49,153] and drifted as water softened nylon [59].

The COF is not a reliable surrogate for wear: at least 15 studies, including orientation and qualitative reports, show friction and wear moving oppositely for the same factor [13,32,34,40,51,53,59,87,134,138,170,198,236,246,291], as did 4 of 28 quantitative pairs (Section 16.3), and in at least four more, only one changed strongly [39,58,64,142].

No study reviewed here tested the Ratner–Lancaster correlation, abrasive wear ∝ 1/(σ_u·ε_u) [118], on printed parts. *Hypothesis (S1), untested:* printed-polymer wear scales with 1/(σ_i·ε_i), the failure strength and strain of the interface the orientation triplet loads in sliding (Section 8)–the interlayer weld under layer shear, the inter-road boundary under raster-parallel sliding or the fibre–matrix interface. Weld anisotropy, hardness-independent path delamination, peak wear under interlayer shear, and weld- or ductility-controlled effects of layer thickness and annealing are consistent with it [27,29,32,41,50,122]. A test needs one polymer in four orientation triplets and three thermal histories, with interface strength and strain, hardness and Sa measured after abrasion and sliding; the hypothesis fails if 1/(σ_i·ε_i) ranks wear no better than 1/H, and should not hold where a transfer or solid-lubricant film controls wear (Section 12).

## 15. Printed Versus Conventionally Processed Polymers

This section classifies printed-versus-conventional comparisons in three tiers: material-matched (the same nominal polymer by both routes), product-matched and not material-matched. As material-matched pairs can still differ in grade, feedstock lot or surface state, conclusions about the route itself rest only on a route-isolated subset (Section 15.1). Of the 60 comparisons in the present evidence base (six added on full-text reading [53,64,92,130,161,251], two without a conventional comparator removed [73,187]), 17 are material-matched (printed better 6, comparable 2, condition-dependent 3, conventional better 6), 20 are product-matched dental comparisons and 23 are not material-matched (Table 6). The last group (different material, literature data or metal-lattice comparators) cannot answer RQ1 [4,8,12,93,101,130,141,173,183,184,189,251].

### 15.1. Material-Matched and Product-Matched Comparisons

Of the 17 material-matched comparisons, four are abstract-only (one of them, in artificial saliva, dental-oriented [307]) and eight used the same feedstock; only three are route-isolated, with printing better in two and similar in one [51,97,314]. A comparison is route-isolated when the same feedstock or formulation was processed by both routes, both specimens were brought to the same surface state (machined, polished or identically coated) and the test conditions were identical; the three pairs are PETG, short-carbon-fibre epoxy and a UHMWPE compound (Table 6). A fourth candidate, a printed coating compared with a blade-coated layer, was removed at revision because printed coatings are outside the eligibility criteria (Section 2.2). This subset limits the conclusion in three ways: it covers three material systems, each tested in a single study; all were tested in dry sliding against steel; and it contains no as-printed surface, so it says nothing about the route as delivered. It indicates that selected, refinished printed materials can perform similarly to or better than their conventional counterparts under the tested conditions; it does not demonstrate general equivalence or an absence of route effects. Moderate- and high-appraisal studies disagree in direction under comparable dry sliding, so the overall comparison for nominally material-matched pairs is conflicting (C4).

The 20 product-matched dental comparisons (different methacrylate or PMMA formulations for one indication) favoured each route in four and were comparable or condition-dependent in six each [10,11,169,174,175,178,179,180,181,188,193,196]. Their dependence on resin class, wear mode and duration is established (Section 6.2); as all specimens were polished, they rank formulations, not routes.

### 15.2. Abrasion, Sliding and Lubricated Contact

The wear mode sets the sign. In abrasion, the conventional material was better in all three material-matched studies, and for five of seven polymers in the largest (Table 6) [50,75,309]. With only one of the three of moderate or high appraisal, this disadvantage is probable; because grits load individual interbead welds, weld strength rather than bulk hardness probably controls removal.

In dry sliding against smooth steel, the evidence is conflicting, printing being better in four studies, similar in one, condition-dependent in three and worse in wear in two (Table 6) [34,51,53,60,64,85,96,97,142,314]. Gains came from surface chemistry (transfer films from print-oxidised PETG [51]) or fibre architecture [97].

In water, as in dry sliding, printed short-fibre and neat PEEK wore more than moulded PEEK (Section 13.2) [53,142]; with two high-appraisal studies from one group, this penalty is probable. For oil and grease, the evidence is insufficient: one low-appraisal study found a higher COF for printed ABS in paraffin oil [295], and two abstracts propose lubricant storage in voids [7,52].

### 15.3. Surface Versus Bulk Contributions

Surface state separates the outcomes. Where both routes were machined or polished (three studies, three groups), the printed material was better or similar (SWR −93%, −68% and +17%; derived; established for these three systems) [51,97,314], within the limits stated in Section 15.1. With as-printed faces, the conventional material won five of nine studies (printed-to-conventional roughness ratio 1.2–6.6 where reported; derived) [50,53,75,142,295]. Porosity also contributes: printed PEEK composites were more porous and softer than moulded ones [142], and density alone changed UHMWPE wear about tenfold (Section 10.6) [60]. Attributing most deficits to as-printed topography and porosity is therefore probable, as no study separates the two; a 2 × 2 design (one feedstock, both routes, as produced and machined to equal Sa, with density and crystallinity measured) would.

Polyamides split the same way: SLS polyamide matched or outperformed moulded polyamide in dry sliding [52,85], whereas extruded PA6 lost 74.6% less volume than printed PA12 in abrasion [50] and moulded nylon gears outlasted printed ones [101] (against literature PA66 data, only at 15 N·m [4]). Grade, crystallinity (56% versus 48% [4]) and wear mode, rather than route, probably explain this; sintering and moulding one PA12 powder and testing both in sliding, abrasion and gear loading would settle it.

## 16. Quantitative Synthesis of Published Results

This section uses within-study effect sizes (treated/reference ratios at one condition in one study) instead of pooled absolute values, because such ratios cancel most between-study differences in rig, counterface and units.

### 16.1. Usable Data

Of the 245 experimental full-text studies analysed, 135 state an absolute COF and 77 give a specific wear rate in mm^3^/(N·m), reported or derivable from reported inputs (Section 11). After exclusion of eighteen implausible, order-of-magnitude-only, secondary or duplicated data sets [16,189,229,230,249,258,262,296,315], 179 usable SWR values of printed specimens remain from 59 studies, 42 of which also report the COF.

### 16.2. Observed Ranges by Material Class

The SWR of printed specimens spans 5.1 decades, from 2.4 × 10^−7^ to 3.0 × 10^−2^ mm^3^/(N·m) (Figure 10): 4.3 decades for unfilled thermoplastics, 4.8 for filled thermoplastics and 4.2 for photopolymers and thermosets (all derived). Class medians differ by about one decade (7.4 × 10^−5^ unfilled, 8.1 × 10^−6^ filled), yet the unfilled range lies entirely within the filled range. Printed neat PEEK alone spans 2.6 decades across 11 studies (derived; Supplementary Dataset S2) [6,13,34,41,49,138,139,140,142,248,300]. Contact configuration, with its associated contact severity, produced the largest contrast observed between groups, although its contribution cannot be separated from differences in pressure, material state and rig: dry, solid (non-lattice) specimens in conformal contacts (flat pin or block on disc, ring or plate, 1–10 MPa nominal) gave a median of 3.9 × 10^−6^ (seven values, five studies), and those under a steel ball (mean Hertzian pressure ≈ 90–260 MPa where reported) 6.1 × 10^−5^ mm^3^/(N·m) (six values, three studies), 1.2 decades higher (all derived; Table S8); within the conformal group, values still span 1.4 decades. Within studies, material condition (annealing, architecture, infill, parameter set) shifted the SWR by 0.15–0.71 decades [34,41,139] and load (14 to 50 N in serum) by up to 1.2 decades [49]; serum and phosphate-buffered saline did not lower the SWR relative to dry contacts of similar geometry [13,49], and exposed infill gave the highest conformal values [34]. As the two biological-fluid studies also differ in counterface and load, lubricant and contact effects cannot be separated. Across rigs, the median SWR rose from 4.7 × 10^−6^ (ring-type) through 2.8 × 10^−5^ (reciprocating) to 4.4 × 10^−5^ (pin- or ball-on-disc) (derived), a shift as large as that between classes, and within single studies, the SWR varied by a median of 0.71 and up to 2.9 decades [41]. Pooling the absolute SWR across heterogeneous test conditions therefore cannot rank material classes reliably (C2).

### 16.3. Within-Study Effect Sizes by Factor

Table 7 and Figure 11 summarise 161 ratios from 78 studies. Fillers lowered the COF in 31 of 35 comparisons and wear in 18 of 22, with mechanistically explicable exceptions (talc without oil, 10 wt% glass fibre in PLA, PTFE in PA12) [58,134,157]. Orientation produced larger anisotropy in wear (median 1.5, up to 7.8) than in the COF (1.2) [73,125]. Thermoplastic annealing lowered wear in 7 of 9 comparisons but raised it in PLA (conflicting; Section 10.1) [41]; photopolymer post-cure, kept separate as it acts through cross-linking, lowered wear in both comparisons [167,172]. Thicker layers raised the COF in every comparison, with a conflicting wear effect (Section 8.2); infill gave medians near unity; and environmental exposure raised the COF in all and wear in most comparisons [58,308]. Outside orientation, median absolute changes were 0.29 decades (≈1.9-fold) for wear and 0.11 decades (≈1.3-fold) for the COF (derived), and in 4 of 28 paired comparisons, the COF and wear moved in opposite directions (orientation excluded; Section 14.4) (S1).

These summaries are robust to non-independence and selection (Table S7). One value per study or per research group shifted medians by at most 0.13 (filler wear, 0.52 to 0.39 per group) without reversing direction; studies whose median ratio indicated a reduction numbered 25 of 27 for the filler COF, 15 of 16 for filler wear and 5 of 5 for annealing, although one annealing study lowered wear for PEEK and raised it for PLA [41]. Restriction to high- and moderate-appraisal studies shifted medians by at most 0.12, and 95% bootstrap intervals over research groups excluded unity for fillers (COF 0.63–0.92; wear 0.27–0.72), annealing (0.24–0.80) and environmental exposure (COF 1.15–1.98; wear 1.18–1.70), but included it for layer height, infill and lubrication (few studies, or conflicting or near-unity effects) (all derived). No association was detected between effect magnitude and citations per year (Spearman ρ = 0.05, *p* = 0.74; 54 studies), so the citation-based prioritisation of records (Section 2.2) did not detectably favour larger effects, although residual selection bias cannot be excluded.

### 16.4. Limits to Statistical Pooling

Five obstacles preclude formal pooling. First, wear was reported as the SWR, volume, mass, depth or linear rate, 14 studies used eight non-standard units, and 26 of the 179 SWR values were derived or converted. Second, only 117 of the 245 studies report any dispersion and 103 report both replicates and dispersion, precluding inverse-variance weighting for most ratios. Third, between-study heterogeneity (class ranges of 4.2–4.8 decades, rig medians one decade apart) dwarfs the median within-study effect of 0.29 decades (derived). Fourth, studies contributing several ratios (formulations or metrics) give non-independent observations, which the per-study and per-group summaries of Section 16.3 address only descriptively. Fifth, ratios use the treatment levels that authors highlight, and publication bias could not be assessed; the absence of a citation–effect association (Section 16.3) argues only against a bias introduced by the present selection. Table 7 therefore indicates only direction and order of magnitude; pooling would require the minimum reporting set (C3), in particular the SWR with n and standard deviation.

## 17. Applications

### 17.1. Gears

Printed gears failed thermally [5,101,152] or at interlayer welds before conventional wear limits; at 15 N·m, moulded PA66 outlasted printed nylon about 6.7-fold (derived) as teeth fractured through incompletely sintered interlayers (Section 15.3) [4]. Printed PEI ran cooler than machined PA6 because of its higher glass transition, not porosity [121]; short carbon fibre [6] and thicker walls (one low-appraisal study) [252] reduced gear wear. No lubricated printed-gear test was identified.

### 17.2. Bearings, Bushings and Seals

For plain bearings, the most promising advantage is lubricant stored in interconnected porosity: probable for designed and insufficient for as-printed porosity (Section 13.5) [7,52,55,56]; even designed oil-filled pores left the COF of PTFE/PEEK only 14% below the solid composite (derived) [199]. Printed CF-PEEK linings ran at a COF of 0.10–0.14, against 0.31–0.39 for Babbitt [8,316], and textured and glass-bead-filled polyamide bearings show pv-dependent running-in [81,124,273]. No printed seal test was identified.

### 17.3. Friction-Controlled Surfaces: Grippers, Conveyors and Footwear

Friction was tuned by Shore grade and infill in TPU conveyor pads [147] and by architected (spiked, morphing, fuzzy-skin, auxetic) surfaces and lattice grippers, without wear data [14,15,95,108,160,317]. Against C45 steel, ABS gripper jaws gave the lowest and TPU 82A the highest COF (0.06–0.12 versus 0.21–0.33) [318]. In footwear, PEBA gyroid lattices lost 3.7–5.8 times less mass than TPU (derived) [149], and fibre content and sole material changed ice friction and wear [17,163]; printed soles and insoles [319,320] lack friction and wear data.

### 17.4. Biomedical and Dental Contacts

Dental evidence is treated in Section 6.2 [11,19,169,185]. Against UHMWPE, oriented short carbon fibre lowered the COF of PEEK, whereas non-oriented fibre abraded the UHMWPE [203], and Si_3_N_4_ reduced PEEK wear but roughened the CoCr counterface [13], so pin-only metrics can mislead. No hip, knee or cross-shear test of a printed bearing polymer was identified [110], although twist tests exist for conventional UHMWPE [321]. Orthoses and prosthetic interfaces also rub against skin, where friction is time-dependent and prone to stick–slip [322,323]; in the only scratch comparison, printed CF-PETG and ABS orthosis materials matched conventional PP up to 50 N [251].

### 17.5. Tooling and Erosion-Exposed Parts

Tooling studies covered sintered polyamide lapping plates, stereolithography mould inserts and Alumide fixtures, the last matching machined aluminium at very low load [161,271,324]. In slurry erosion, infill architecture dominated [256,278,325], macro-prisms eroded 71% less across the jet but 28% more along the channels [277], and photopolymer impellers mapped wear for a metal pump [162].

### 17.6. Where Printing Gives a Genuine Tribological Advantage

Four advantages go beyond design freedom: designed porosity or embedded lubricant [7,56,154,199]; graded or multi-material faces [16,40]; printed textures [150,274,277]; and compliant lattices that damp friction-induced vibration [15,41]. Each is at most probable, resting on one to three laboratory studies without component-life data.

## 18. Current Limitations and Research Gaps

### 18.1. Evidence Grade of Each Link in the Framework

Grades follow Section 2.5 (Figure 1, Table 8); “not identified” means not identified in the prioritised, access-shaped evidence base (Section 2). Evidence is strongest near the printer and for mechanism switching, and weakest for contact mechanics and durability, on which design prediction depends.

Scope differs among the established findings (Table S6). Some hold across at least two routes or material classes: the orientation-associated switch of wear mechanism, loss of topographic friction control with rising load, transfer-film control of mild wear, friction reduction by PTFE and lamellar solids, oxidative wear, subsurface fatigue under cyclic contact (dental resins and gears) and the failure of hardness as a general wear predictor. Others are confined to one route, polymer family or condition and should not be transferred without new data: the annealing benefit for dry FFF PEEK, friction reduction by oil and grease (shown for extruded thermoplastics), end-on fibres as the worst case in dry FFF composites, the layer-height effect on the COF and the exposed-infill effect in material extrusion, and the post-cure and formulation findings for dental photopolymers. Seven established grades would not survive all stricter variants of the grading rule combined (Section 2.5) and are flagged in Table S6.

*Process → architecture: established for material extrusion and powder bed; probable for vat photopolymerisation.* Extrusion settings and annealing change interlayer bonding, porosity and crystallinity as neck-growth models predict [4,26,27,127], energy input and scan strategy set powder-bed density and crystallinity [40,60,137], and one vat study links post-treatment to hardness [172]. With crystallinity or conversion measured in only 44 of the 245 studies (Section 11.2), most process effects on wear are inferred, not traced.

*Architecture → surface: established for material extrusion; insufficient for powder bed.* Layer height and raster set areal roughness (Sa 5.5–19.1 µm [64,116]) and stair-stepping [127,131]; no parametric powder-bed surface study was identified.

*Surface → contact: probable.* Loss of topographic friction control with rising load is established [35,64,115,116], its threshold pressure is insufficient (not transferable), and spacing, shape or bearing-ratio parameters explain friction better than Ra [37,244]. No as-printed surface had its real contact area measured under load; only a morphing surface [14] and printed rubber-friction counter-surfaces [106] link topography to contact quantitatively.

*Contact → mechanism: established for mechanism switching.* Orientation is associated with a reproducible switch between interlayer-shear- and ridge-crossing-dominated wear, inferred from wear morphology and orientation response, not observed operando. The best orientation is conflicting except for dry PLA and ABS on steel, where edge-on contact with layers parallel to sliding is consistently worst (established) [32,116,125]; filaments parallel to sliding minimised PEEK wear dry [140] and in serum [143]. The shear-versus-channelling explanation (Section 8.1) is probable, and “vertical”/”horizontal” labels remain conflicting, as only 43 of the 245 studies fully state the triplet.

*Bulk bypass, architecture → mechanism: conflicting.* Annealing lowers the dry wear of printed PEEK (established, conditional on load and filament direction) but conflicts for PLA [41,127]; a bulk origin of the PEEK gain is probable, as is higher crystallinity lowering wear while raising the COF (one group, confounded with porosity) [40]. Hardness alone is not a transferable wear predictor (established): r = −0.93 across ten occlusal-device materials reflects two mechanism clusters [11], and abrasion, chewing and sliding show no consistent relation [10,50,65].

*Mechanism → durability: probable.* Two moderate-appraisal gear studies tie component life to interlayer root fracture [4] or thermal failure [101], with low-appraisal rig and clinical support [19,152]; no study linking a measured architectural state to component life was identified.

### 18.2. Methodological and Coverage Gaps

No AM-specific test standard was identified; only dentistry shares a simulator protocol [169], and rankings change with the test method [173,182]. Only 3 of the 245 studies report all eight core contact and statistics elements (Table 5; Section 11.2 and Section 11.3), and implausible values recur [164,228,254,296].

Replication is weak: one [4,33] or two [42,60] specimens per condition are common, centre-point replicates varied up to 4.2-fold (derived) [260,261], and five nominally identical PLA filaments gave a mean COF of 0.246–0.368 [42]. Only 7 of the 245 studies quantified counterface wear (Section 11.3), although fillers can shift damage to the counterface [13,207].

Tests lasting from seconds to 300 s [54,124,238,239,318] capture only running-in or breakaway, and rankings can change after 105 min [293]. Lubricated tests reach only boundary or early mixed conditions (λ = 0.013–0.06 [8,49]), lubricant uptake was never measured, and ageing ends at 28 days or 400 thermal cycles [57,58]. Rolling contact [253] and fretting [326] are insufficient, whereas subsurface damage under cyclic contact is established from dental tests [93,185]. Post-test tomography was rare [40,116], no operando observation of interlayer cracking was identified, and models are evaluated on the series they were fitted to [32,62,232]. Material and application coverage gaps are listed in Table 8 [50,60,137,151,314].

## 19. Future Research Directions and Roadmap

### 19.1. A Standard Protocol and Shared Data

A two-tier protocol would remove most of the incomparability documented in Section 11. Tier 1 (screening) would use reciprocating ball-on-flat or pin-on-disc contact against bearing steel of stated hardness and Sa, two pv levels (well below and near the thermal limit), three orientation triplets, a steady-state distance criterion, n ≥ 3 and profilometric wear volume of both bodies. Tier 2 would use application-specific component rigs (gear, journal bearing); dental wear testing should follow or adapt ISO/TS 14569-2 [169]. Both tiers would report the minimum set (Section 11.4) and the feedstock lot [42]. A round robin on one lot would quantify between-laboratory variance, the baseline for judging any model, and raw COF traces and ISO 25178 files [266,270] would form an open benchmark dataset for effect-size synthesis (Section 16).

### 19.2. Physics-Based and Data-Driven Models

Beyond anisotropic finite-element and orientation-vector models evaluated only in-sample (Section 18.2) [32,62,232] and Persson theory applied to printed rough surfaces [106,114], we propose, as a testable hypothesis, an interlayer-aware Archard–Lancaster–Ratner form [113,118] with an added term proportional to the sheared interlayer area fraction and inversely proportional to interlayer fracture energy. It predicts that the along-layer to across-layer wear ratio rises as weld strength falls (lower nozzle temperature) and approaches unity after interface-healing annealing.

Against replicate scatter of up to 4.2-fold (Section 18.2) [260], fits with R^2^ ≥ 0.98 or errors below 2% [228,243,286,287,328] and identical regression statistics in two papers [262,263] suggest in-sample evaluation or overfitting; an R^2^ of 0.80–0.84 [239,241] and MAPE of 12.43–23.02% for 48-month dental wear [196] are more credible. Models should use intermediate-state inputs (crystallinity, interlayer strength, Ssk and Sv [116]) rather than process settings alone. The interlayer-aware model should be validated in three steps: calibration on one printer and laboratory; prediction, without refitting, of builds from other printers and feedstock lots tested under the minimum reporting set (Section 11.4); and leave-one-laboratory-out prediction on a shared round-robin dataset. As replicate scatter is of the same order as factor effects, uncertainty should be explicit: replicate distributions rather than means as inputs, parameters estimated with hierarchical (printer-, lot- and laboratory-level) random effects, and prediction intervals checked for coverage on held-out data.

### 19.3. In Situ Monitoring

Tribo-acoustic noise [239], friction-induced vibration [15,41,254], gear vibration [101] and infrared thermography [4,8,97,152] tracked running-in, heating and failure without being linked to mechanisms; acoustic emission and operando microscopy of printed contacts were not reported. Printed texture changes the tribocharging of polymer slabs in conformal contact [329], making tribo-electric signals a candidate, still untested, wear indicator. Pairing acoustic emission with interrupted micro-CT would test whether interlayer crack growth has a detectable signature before wear accelerates.

### 19.4. Designed Architectures and Sustainable Feedstocks

Software for lattice and functionally graded design is mature [330] but rarely targets tribological objectives. Designed contacts (Section 17.6) [16,40,56,154,199,301], placed self-lubricating fillers [91,134] and aligned fibres [6,62] should be judged against both homogeneous end-members at equal conditions over steady-state distances, and accepted if they give a Pareto improvement in the COF and SWR or a stated application-specific advantage, with retained lubricant reported against distance. Recycled and bio-based feedstocks changed wear in material-specific directions [17,79,246,327]; life-cycle assessments need wear against reprocessing cycles and debris release, and material savings from generative design [331] count only if redesigned contacts reach the same wear life.

### 19.5. A 5–10-Year Roadmap

Years 0–3 need the Tier 1 protocol, a round robin and the benchmark dataset; years 3–6, interlayer-aware models, machine learning validated on held-out printers and laboratories, in situ sensing and replicated component tests including the first printed seals; and years 6–10, process–structure–tribology maps per material class, qualified graded and multi-material contacts, recycled-feedstock qualification and digital twins validated against field data (Figure 12).

## 20. Conclusions

(1) Established findings, each supported by at least three studies from at least two research groups: transfer films control mild wear; interlayer weld failure is the wear mechanism most specific to material extrusion; orientation is associated with a switch between interlayer-shear- and ridge-crossing-dominated wear; topographic control of friction is lost as load rises; short carbon fibre in engineering matrices, PTFE and lamellar solids, and oil or grease lower friction; annealing lowers the dry wear of FFF PEEK (conditional on load and filament direction); post-cure lowers the wear of photopolymers; and hardness alone is not a transferable wear predictor. Conflicting are water and hygrothermal effects, the best orientation across polymers, the wear effect of short carbon fibre and the direction of printed-versus-conventional differences; the three comparisons that isolate the manufacturing route indicate that dense, refinished printed material can perform similarly to or better than its conventional counterpart under the tested conditions, without demonstrating general equivalence.

(2) The largest within-study effects (derived) were associated with fibre orientation (up to 7.8-fold in wear) and powder-bed crystallinity or density (about tenfold), followed by photopolymer post-cure (median ≈2.8-fold lower wear) and contact-face orientation (1.15–6.2-fold, shrinking with load); nozzle temperature, layer height, infill beneath a skin and print speed had smaller effects. This ordering is indicative, and replicate scatter of up to 4.2-fold rivals many factor effects.

(3) Friction and wear are often decoupled: the COF and wear moved in opposite directions in 4 of 28 paired within-study ratios and in at least 15 studies on a broader screen. Three mechanisms account for this: sliding across layer edges raises the COF through ridge interlocking yet lowers wear because the welds are loaded across rather than along their length; higher crystallinity raises the COF while lowering wear by strengthening the surface and stabilising the transfer film (probable); and lubricating fillers such as PTFE lower the COF but raise wear where the matrix cannot support the film. The COF is therefore not a wear surrogate.

(4) Only 3 of the 245 full-text studies analysed (1.2%) report the eight core contact and statistics elements, and 17.6% fully state the orientation triplet. For printed neat PEEK, median specific wear rates differ by about 1.2 decades between conformal and concentrated contacts, so absolute values do not transfer between studies, and a formal meta-analysis is not defensible.

(5) Several explanations remain hypotheses (probable): interface shear versus debris channelling as the cause of orientation reversals, a contact-severity crossover for fibre orientation, embrittlement versus densification for annealed PLA, and lubricant storage in designed, impregnated porosity. Design prediction rests on links that are only probable (surface → contact, mechanism → durability). Priorities are orientation-triplet factorials, interlayer fracture toughness, treat-then-refinish designs, wear of both bodies, a round robin under the minimum reporting set, replicated steady-state component tests and interlayer-aware wear models validated across printers and laboratories.

## Figures and Tables

**Figure 2:**
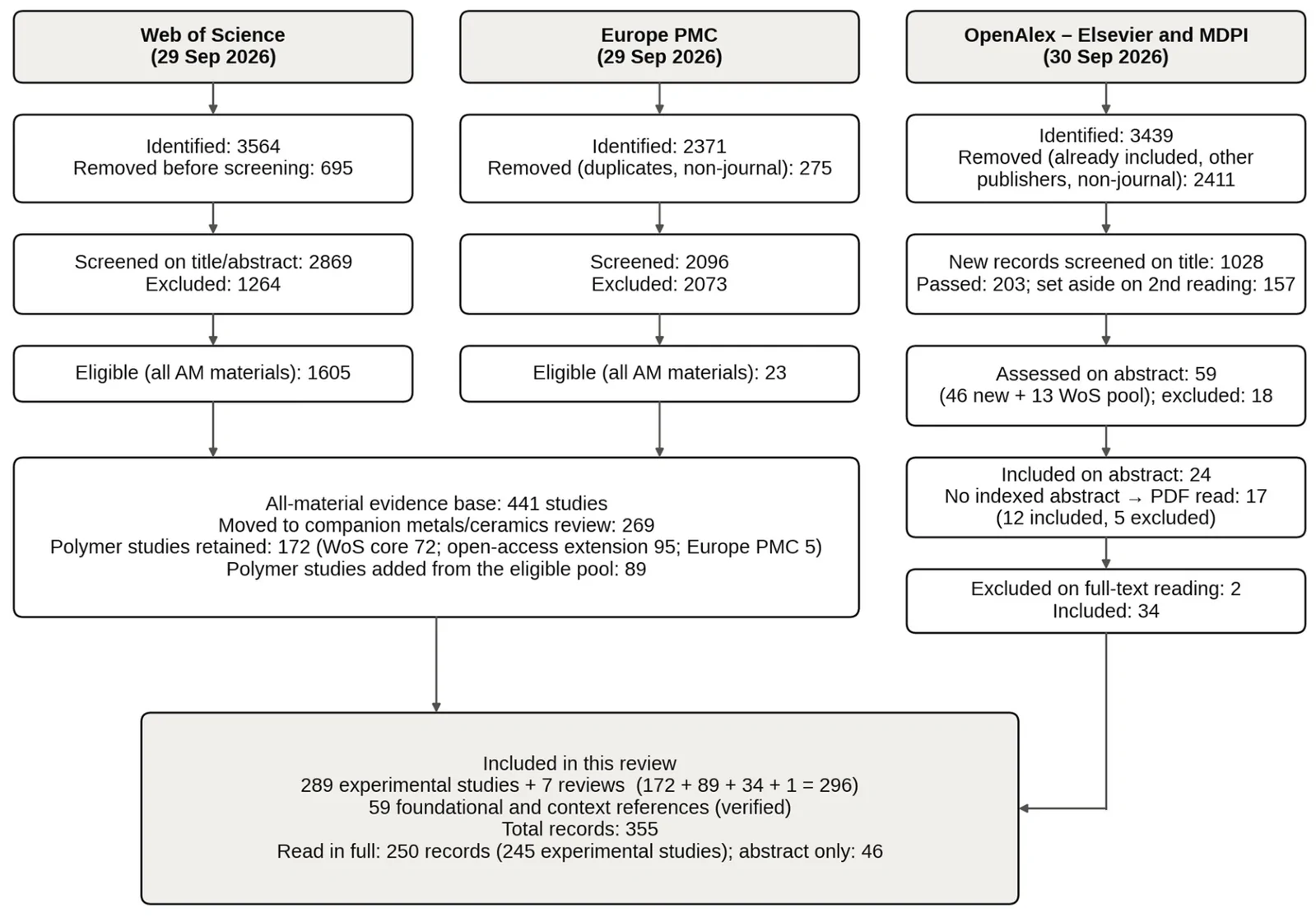
PRISMA-style flow of record identification, screening, eligibility, scope restriction and inclusion.

**Figure 9:**
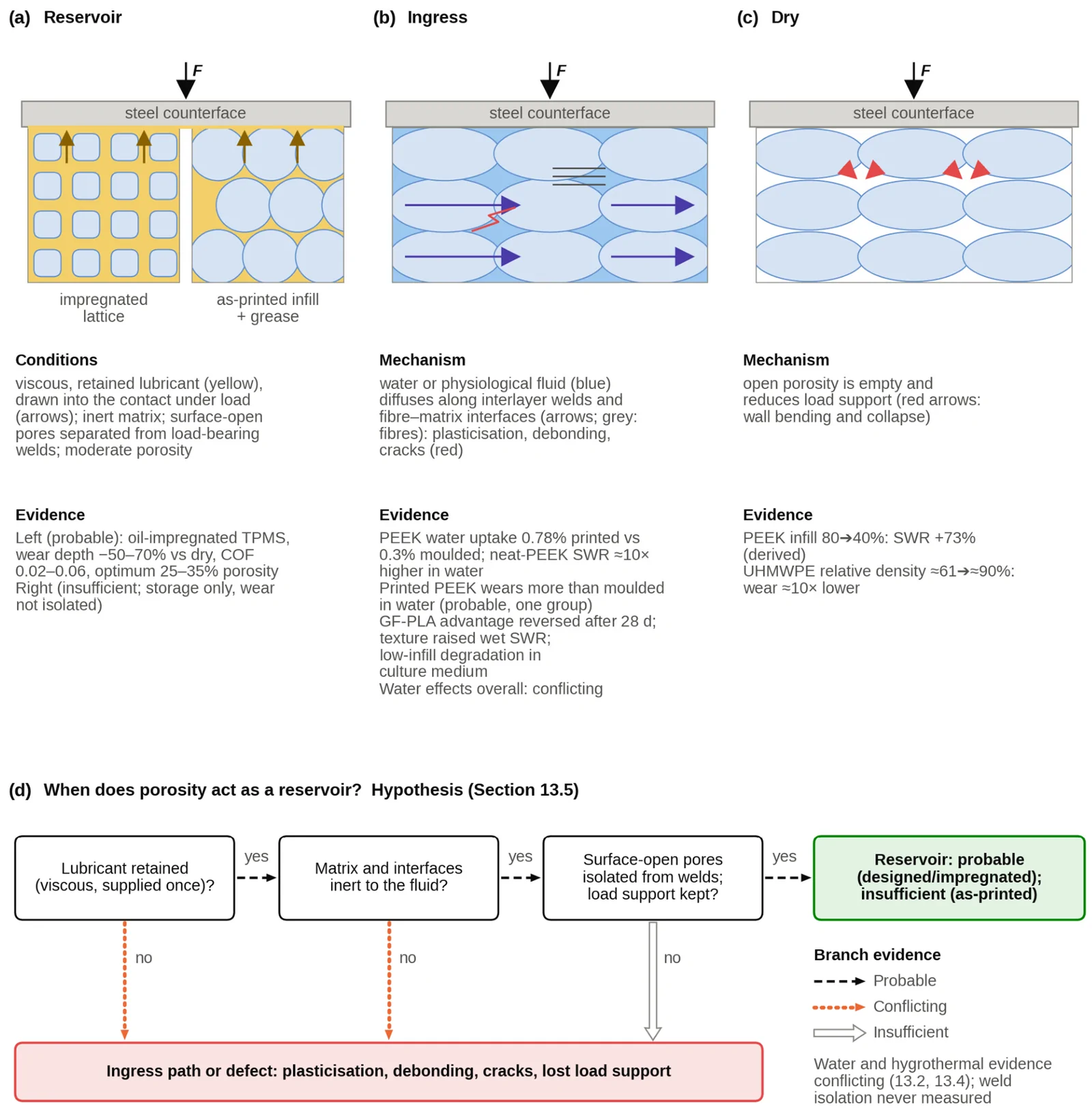
Porosity in printed polymers as a lubricant reservoir or an ingress path: conditions for each outcome. The reservoir outcome is probable for designed/impregnated porosity and insufficiently evidenced for as-printed porosity. Sources: (**a**) impregnated lattice [56] and as-printed infill with grease [7,8,52,54]; (**b**) [53,57,59,142,249]; (**c**) infill density [34] and UHMWPE relative density [60].

**Figure 10:**
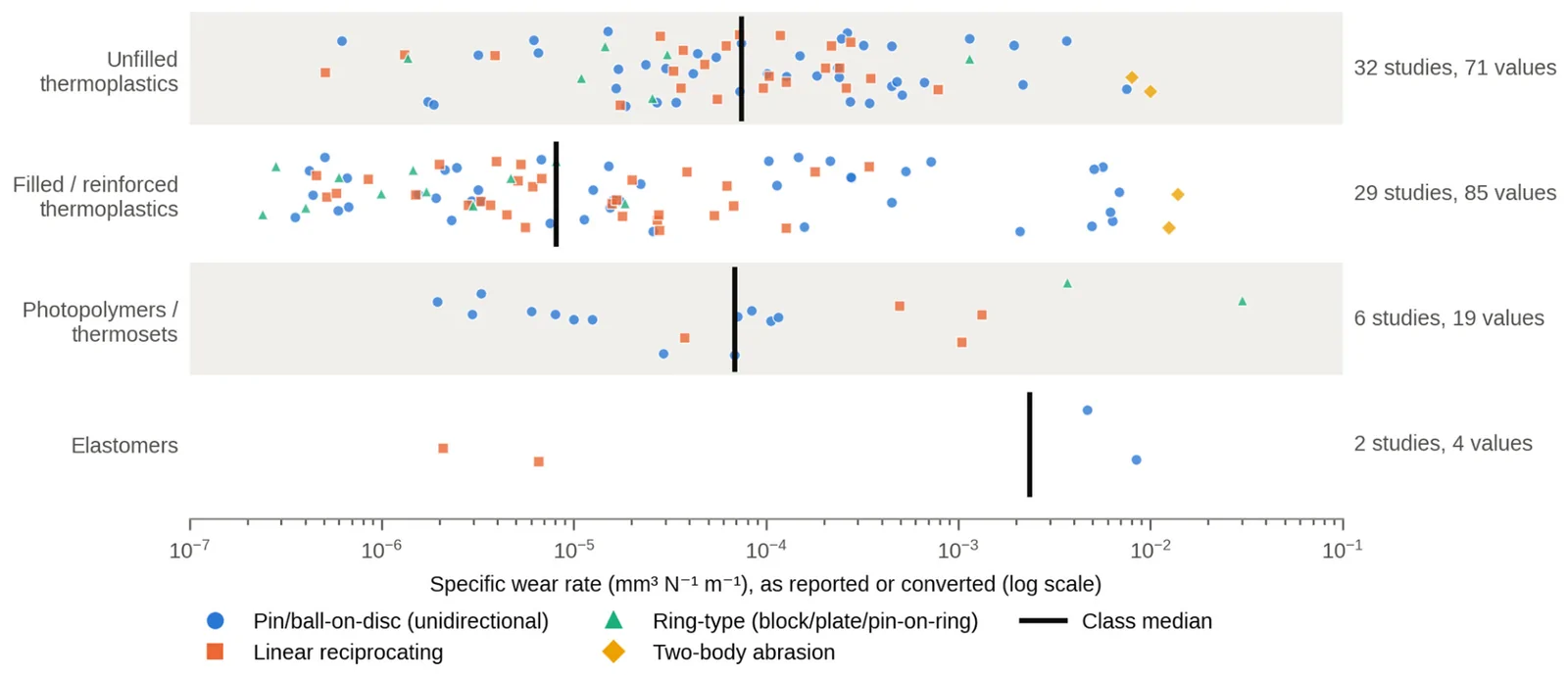
Specific wear rates of printed polymers by material class (179 values from 59 studies; unfilled thermoplastics 32 studies and 71 values, filled 29 and 85, photopolymers/thermosets 6 and 19, elastomers 2 and 4), with marker shape and colour giving the test configuration and vertical bars the class medians. The spread within each class exceeds the differences between classes.

**Figure 11:**
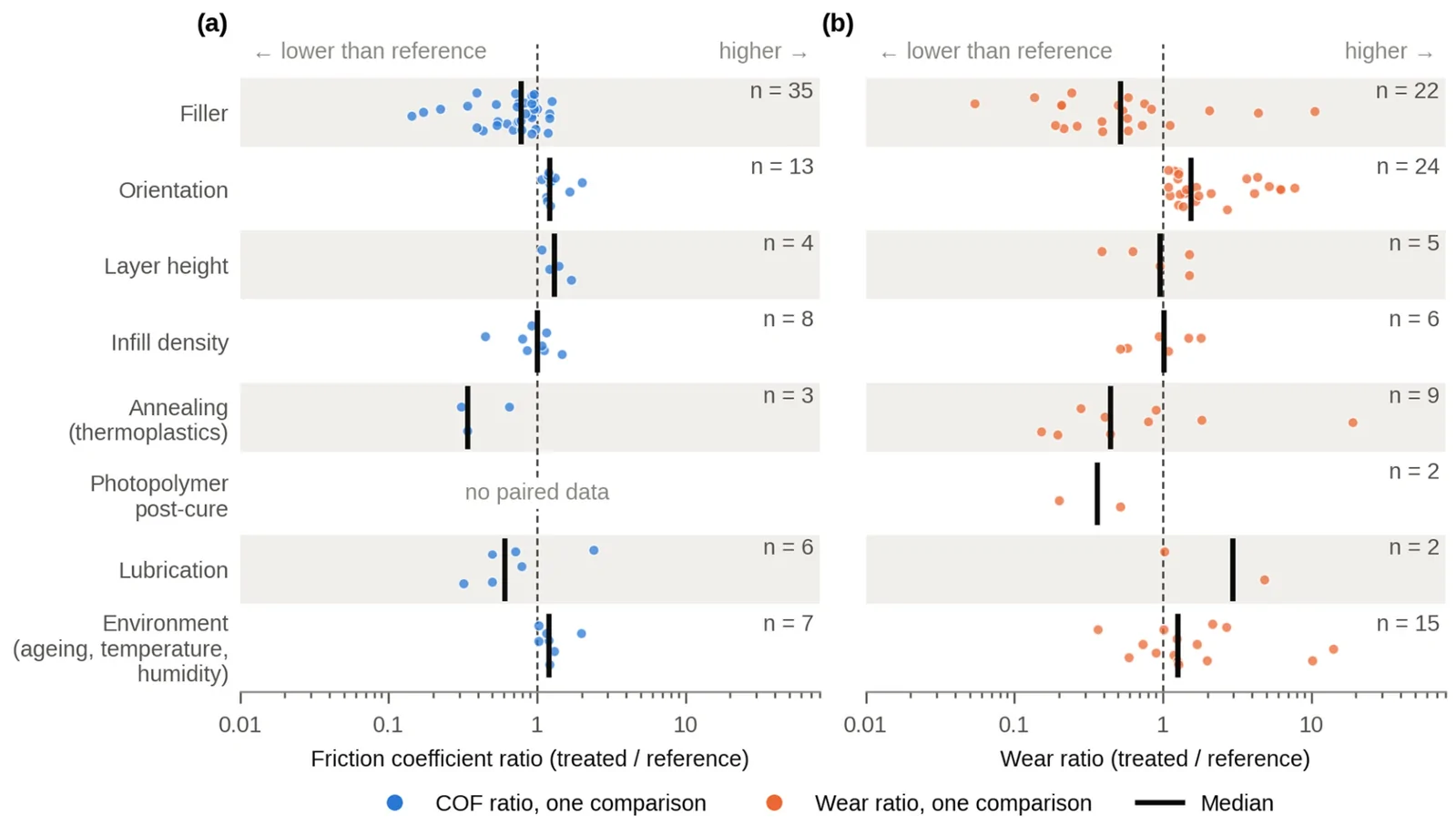
Within-study effect sizes of printing and test variables on friction and wear of printed polymers. Each point is one ratio of treated to reference (orientation: highest to lowest configuration) from one study, for the COF ((**a**); 76 ratios) and wear ((**b**); 85 ratios), 161 ratios from 78 studies in total; vertical bars mark factor medians and n gives the number of comparisons per factor.

**Figure 12:**
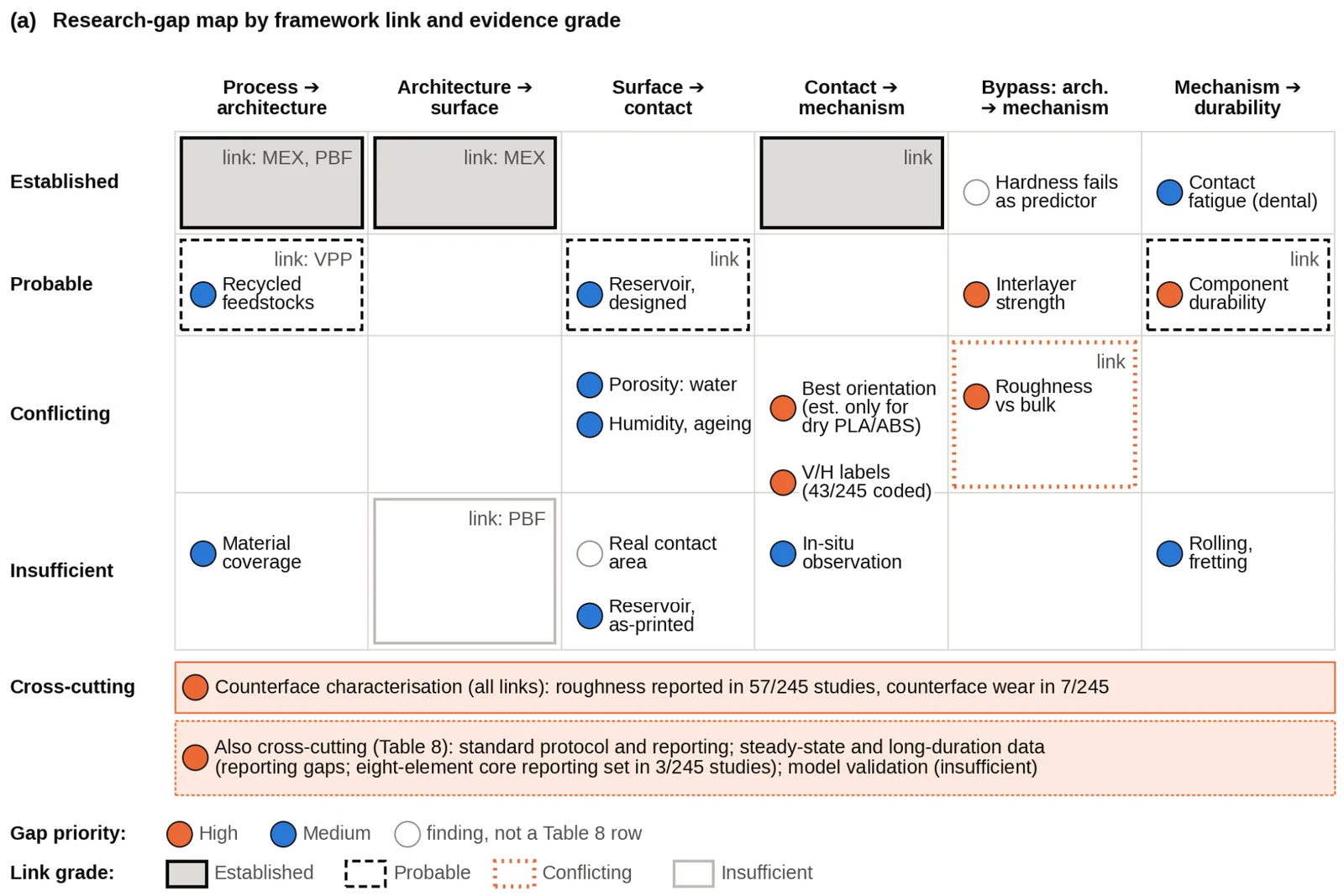

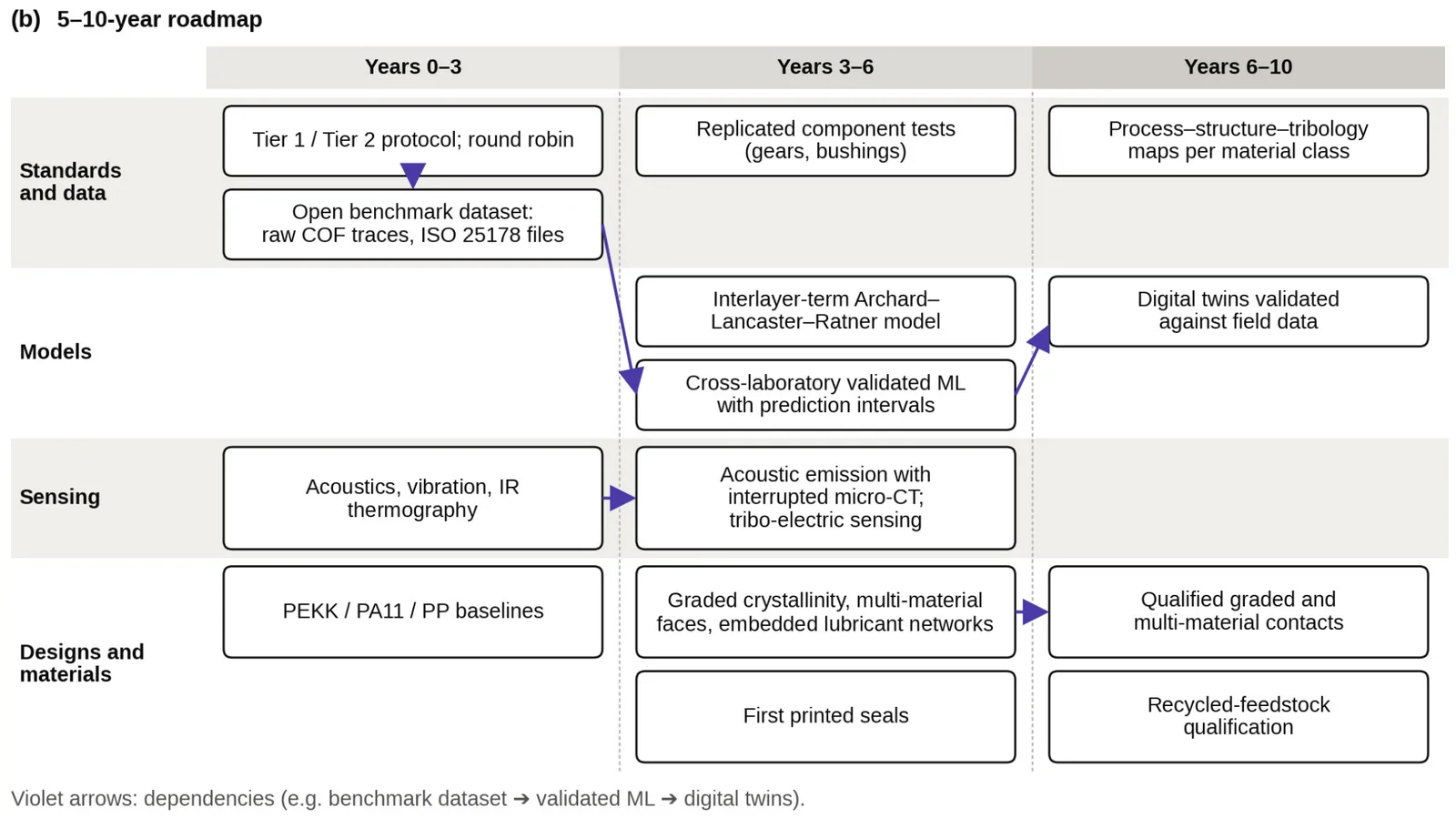
Research-gap map and 5–10-year roadmap for the tribology of printed polymers and composites. Sources for (**a**): orientation [32,140]; interlayer strength [11,50,76]; real contact area [14,106]; porosity [56,58,199]; fatigue [93,253]; mechanism → durability link [4,101]; component durability [4,19]. In (**b**): acoustics, vibration and infrared thermography [4,15,239]; graded and multi-material contacts [16,40,56].

**Table 5:** Minimum reporting set for tribological studies of printed polymers.

Item	What to Report	Reported in the 245 Full-Text Experimental Studies Analysed
1. Contact geometry and printed body	Configuration and standard; which body is printed; pin/ball size, track radius or stroke; polymer–polymer or polymer–metal	Configuration identifiable 99.6%; standard cited 33.1%
2. Orientation triplet	{contact face, layer normal, raster/fibre direction} relative to sliding (powder bed: scan or spreading direction; Section 8.1); as-printed or machined face	Fully stated 17.6%; not determinable 46.5%
3. Load, speed, pressure	Normal load; linear velocity in m/s; nominal or Hertzian pressure; pv	Load 86.9%; speed/frequency 89.0% (linear velocity 63.3%); pressure 24.9%
4. Duration and steady state	Sliding distance or cycles; running-in length and criterion for steady-state COF and wear	Distance/duration 82.4%; running-in or steady state discussed 18.4%
5. Counterface and specimen surfaces	Counterface material, hardness, Sa or Ra (direction); specimen Sa or Ra before testing	Material 91.4%; hardness 18.4%; roughness 23.3% (grit only 6.5%); specimen roughness 32.2%
6. Environment	Medium (dry, lubricant, fluid); temperature; relative humidity; conditioning of specimens	Environment named 90.6%; temperature 46.9%; humidity 22.4%
7. Wear metrics	Volume loss and method; specific wear rate in mm^3^/(N·m); steady-state COF; counterface wear	Volume 53.1%; mass only 33 studies; author-reported SWR 23.7%; COF value 55.1%; counterface wear 2.9%
8. Replication and statistics	n ≥ 3 specimens per condition; SD or CI; statistical test	Replicates 69.4%; dispersion 47.8%; both 42.0%; test 31.8%
9. Feedstock and bulk state	Supplier, grade and lot; drying; crystallinity or degree of conversion; density or porosity; elastic modulus, Poisson’s ratio and hardness (measured or cited)	Crystallinity or degree of conversion measured 18.0%

Percentages derived from keyword screens of the full-text extraction of the 245 experimental studies analysed (5 reviews excluded); values are approximate and describe the present evidence base only. SWR, specific wear rate; COF, coefficient of friction; CI, confidence interval.

**Table 6:** Printed versus conventionally processed polymers: material-matched comparisons.

Polymer	AM Route	Conventional Route	Test/Wear Mode	Surface State	Result (Values from Full Text)	Direction	Likely Cause	Ref.
PDMS/nano-silica ink	DIW, UV-assisted	Cast, same ink	Reciprocating ball-on-flat vs. steel, 1–3 N; dry	Filament texture vs. flat cast	COF ≈1.0–1.2 vs. ≈1.4 (1 N); cast track up to 1760 µm wide, printed no visible wear	Printed better	Texture reduces real contact area	[96]
PETG	FFF	Compression moulding, same feedstock	Pin-on-disc vs. 440C, 1 MPa, 0.20 m/s; dry	Both polished	SWR 3.4 × 10^−5^ vs. 4.7 × 10^−4^ mm^3^/(N·m) (−93%, derived); COF 0.36–0.54	Printed better	Print-induced oxidation, cohesive transfer film	[51]
SCF/epoxy	DIW, helical layup	Cast and machined, same ink	Ball-on-disc vs. GCr15, 10 N; dry	Both polished	COF 0.27 vs. 0.56; SWR 2.92 vs. 9.13 × 10^−5^ mm^3^/(N·m)	Printed better	Fibre architecture; voids and fibre settling in cast	[97]
ABS	FFF	Injection moulding, other grade	Reciprocating flat-on-flat vs. 316, 5–10 MPa; dry	As-printed (Sa 8.67–19.08 µm) vs. moulded	COF 0.18–0.23 vs. ≈0.3–0.4; SWR same order (10^−5^ mm^3^/(N·m))	Printed better (COF); wear comparable	Grade (butadiene content) differs	[64]
PEEK	FFF	Extruded rod	Journal bearing, PAO grease	n.r.	Abstract only	Printed better	Grease retained in voids (proposed)	[7]
Polyamide	SLS	Injection moulding	Pin-on-disc, dry and oil	n.r.	Abstract only	Printed better (dry)	Lower ductility; oil uptake by pores (proposed)	[52]
UHMWPE/HDPE-g-SMA/PP	Pellet FFF	Hot pressing, same compound	Block-on-ring (wear), ball-on-disc (COF); dry	Both milled	SWR 2.55 vs. 2.18 × 10^−5^ mm^3^/(N·m) (+17%, derived); COF 0.09 vs. 0.08	Comparable	Equal density (0.93 vs. 0.94 g/cm^3^)	[314]
PEEK	Printing (n.r.)	Extrusion	Reciprocating vs. enamel-coated CoCr; dry and saliva	n.r.	Abstract only	Comparable	—	[307]
PEEK	FFF, 40–90% infill	Extruded rod	Pin-on-disc vs. SUJ2, 20 N, 0.2 m/s; dry	As-printed (Sa 24.5–27.9 µm) vs. polished	SWR 7.4 (80%) and 12.8 (40%) vs. 8.43 × 10^−5^ mm^3^/(N·m); crystallinity 33.2–37.1 vs. 38.4%	Condition-dependent (infill)	Porosity and crystallinity; roughness confounded	[34]
PA12	SLS, 0/45/90°	pvT moulding, same powder	Pin-on-disc vs. 100Cr6 (Rz 0.5–3.0 µm), 0.5 m/s; dry	As-built vs. moulded	45° ≈ moulded, 90° lower wear; moulded slightly lower friction (figures only)	Condition-dependent (orientation, counterface)	Layer orientation; counterface roughness	[85]
UHMWPE	L-PBF + heat treatment/CIP/HIP	Machined extruded bar	Pin-on-disc vs. steel (Sa 1.14 µm), 1 MPa; dry	Post-processed vs. machined	Linear wear 1.86 × 10^−3^ (≈90% density) and 1.86 × 10^−2^ (≈61%) vs. 1.53 × 10^−3^ µm/m	Condition-dependent (density)	Porosity	[60]
ABS, PC, PMMA, PETG, PLA, HIPS, PP	FFF	Extruded sheet	Three-body abrasion, rubber wheel + Al_2_O_3_, 44 N	As-printed vs. extruded	Extruded volume loss 9.6–21.6% lower (5 polymers); 16.3% (HIPS) and 34.1% (PP) higher	Conventional better (5 of 7)	Path delamination	[50]
ABS	FFF	Compression moulding, other supplier	Ball-on-disc vs. EN31, 20–55 N; paraffin oil	Ra 1.88 vs. 0.28 µm	COF 0.040–0.055 vs. 0.009–0.025; wear in figures only	Conventional better (COF)	Roughness (6.6×, derived)	[295]
SCF/PEEK, SiO_2_-SCF/PEEK	FFF	Compression moulding, same powders	Block-on-plate vs. 316L, 20 MPa; dry and water	Sa 4.3–4.8 vs. 1.0–1.1 µm	COF not different (*p* = 0.58 dry, 0.16 water); moulded SWR ≈32–40% lower	Conventional better (wear)	Porosity 3–5.2 vs. <1%; HV 11–22% lower; roughness	[142]
PETG, PETG/PTFE/h-BN or EG	FFF/pellet AM	Injection moulding, same compounds	Pin abrasion on SiC P120, 10 N	Ra 1.2–4.2× moulded (derived)	Printed SWR consistently higher (values not extractable)	Conventional better	Voids, weak interlayer adhesion, roughness	[75]
PEEK	FFF	Injection moulding (machined)	Reciprocating flat-on-flat vs. 316L, 80 N; dry and water	Sq 5.6 vs. 2.0 µm	Dry COF ≈ 0.40 for both (*p* = 0.78); lower moulded SWR dry (*p* = 0.009) and in water	Conventional better (wear)	Porosity; water uptake 0.78 vs. 0.3%	[53]
ABS, PETG	FFF	Moulding	Abrasion after UV/moisture ageing	n.r.	Abstract only	Conventional better	Ageing degrades printed ABS most	[309]

Rows are ordered by direction. Of the 60 comparisons in the present evidence base, 17 are material-matched (listed), of which three are route-isolated (same feedstock or formulation and equal surface finish: PETG, SCF/epoxy and UHMWPE compound rows); 20 dental comparisons are product-matched (same polymer family and indication, different formulation) and 23 are not material-matched (Section 15). n.r., not reported; SCF, short carbon fibre; DIW, direct ink writing; pvT, pressure–volume–temperature moulding device. Values marked “derived” were calculated from full-text values.

**Table 7:** Within-study effect sizes by factor.

Factor	n Studies	n Comparisons	Median COF Ratio (Range)	Median Wear Ratio (Range)	Comments	Key Refs
Filler (filled/neat)	31	39	0.77 (0.14–1.25), n = 35	0.52 (0.05–10.5), n = 22	COF lower in 31/35, wear in 18/22; wear rises with talc, 10 wt% GF in PLA and PTFE in PA12; largest COF reductions with oil-loaded fillers, largest wear reduction with CF in PEEK	[6,58,80,134,142,154,157,199,300]
Orientation (highest/lowest)	23	35	1.22 (1.07–2.00), n = 13	1.54 (1.09–7.76), n = 24	Ratio ≥ 1 by construction; end-on fibres and layer-parallel sliding give the largest wear anisotropy; COF and wear rankings often inverted	[62,73,116,125,126,181,193]
Annealing, thermoplastics (treated/as printed)	6	10	0.34 (0.31–0.65), n = 3	0.44 (0.15–19.1), n = 9	Wear lower in 7/9; PLA reverses (1.8–19×; conflicting across studies; Section 10.1); authors’ optimum levels	[41,51,55,127,139,246]
Photopolymer post-cure (post-cured/not post-cured)	2	2	—	0.36 (0.20–0.52), n = 2	UV (LED) post-cure and 121 °C steam treatment; wear lower in both; cross-linking, not crystallisation	[167,172]
Lubrication (lubricated/dry)	3	6	0.61 (0.32–2.41), n = 6	2.94 (1.02–4.85), n = 2	Grease, oil and water lowered the COF; one ionic liquid raised the COF and wear; wear data from one study	[8,53,306]
Environment: ageing, temperature, humidity (exposed/reference)	8	18	1.20 (1.02–1.98), n = 7	1.26 (0.37–14.0), n = 15	COF up in 7/7, wear up in 11/15; high humidity lowered wear of dental resins in one study [104]	[58,103,104,246,308]
Layer height (thicker/thinner)	7	8	1.31 (1.08–1.70), n = 4	0.95 (0.39–1.51), n = 5	COF increase established; wear direction conflicting (Section 8.2)	[166,238,239,241,242,243]
Infill density (higher/lower)	8	13	1.00 (0.45–1.47), n = 8	1.02 (0.52–1.80), n = 6	No transferable direction beneath a skin; monotonic when infill is exposed (Table 3); elastomers and lattices non-monotonic	[34,107,147,149,238,244]

Ratios are treated/reference at one test condition within one study (orientation: highest/lowest configuration), with the same wear metric (SWR, volume, mass, depth or linear wear) for both members of a pair. All ratios, medians and ranges are derived from full-text values, normally one comparison per study, formulation and metric, at the treatment level the authors highlight. Unweighted; not a meta-analysis. Data: Supplementary Dataset S1.

**Table 8:** Major research gaps and recommended research strategies.

Gap	Current Evidence Status	Why It Matters for Design	Recommended Experiment or Strategy	Priority
Orientation triplet effects	Mechanism switching established; best orientation conflicting (established only for dry PLA/ABS on steel); “vertical/horizontal” labels conflicting; shear-versus-channelling explanation probable; triplet fully stated in 43/245 studies [32,33,62,140,143]	Decides which face of a part should carry the contact	Factorial of contact face × layer normal × raster/fibre direction on one material, with printed body and counterbody specified	High
Interlayer strength as a wear descriptor	Probable; interlayer bonding and wear co-vary in two composite studies [76,100]; interlayer fracture toughness not related to wear in any study identified	Sliding along layers activates interlayer shear (Section 12); design needs a measurable property	Mode II interlayer fracture toughness and the SWR on the same builds across nozzle temperature and annealing	High
Roughness versus bulk effects	Bulk bypass conflicting; bulk origin of annealing gains probable; no study identified varies treatment and refinishing factorially with measured Sa (three studies refinish all specimens to one common finish after annealing, without as-printed controls) [51,99,140]	Decides whether to specify finishing or process changes	2 × 2 factorial: treated/untreated × as-printed/refinished to one measured Sa	High
Counterface characterisation and wear	Reporting gap: counterface roughness in 57/245 and counterface wear in 7/245 studies; filler-induced counterface damage documented [13,207]	Life of the mating part; a pin-only SWR can misrank fillers	Steel/ceramic counterfaces at two Sa levels; wear volume of both bodies	High
Standard protocol and reporting	Reporting gap: no AM-specific standard identified; method-dependent rankings [173,182]; eight-element core subset in 3/245 studies	Cross-study comparison and pooled synthesis are severely limited	Inter-laboratory round robin on one feedstock lot; minimum reporting set (Section 11.4)	High
Steady-state and long-duration data	Reporting gap: many tests last minutes [238,239]; rankings shift with time [293]	Component life is set by steady-state wear and fatigue	Run to a steady-state criterion; report wear–distance curves	High
Model validation	Insufficient; no independently validated model identified; near-perfect accuracies reported [228,286]	Design prediction requires transferable models	Leave-one-printer- and leave-one-laboratory-out validation on shared data	High
Component and field durability	Mechanism → durability probable (two gear studies [4,101]); no measured architectural state linked to component life; clinical data only in dentistry [19]	Qualification of gears, bushings and implants	Replicated, lubricated component tests with in situ monitoring	High
Porosity as reservoir or ingress path	Oil/grease lower the COF established; reservoir benefit for wear probable for designed, oil-impregnated porosity (PAO10-filled interconnected pores [199]; oil-filled lattices [56]) and insufficient for as-printed porosity [7]; water and hygrothermal effects conflicting [58,209]	Bearings, marine and biomedical parts	Same polymer, dense vs. porous; measure lubricant uptake, Stribeck curves and λ	Medium
Fatigue, fretting and rolling	Rolling and fretting insufficient [253,326]; subsurface contact fatigue established in dental tests [93,185]	Gears, rollers, snap-fits, implants	Twin-disc tests with defined slip; fretting maps; subsurface crack tomography	Medium
In situ and multi-scale observation	Insufficient [15,40,239]	Onset of interlayer failure is not detected	Acoustic emission, vibration and thermography with interrupted micro-CT	Medium
Humidity and ageing	Water and hygrothermal effects conflicting; 55/245 studies state humidity; ageing ≤ 28 days [57,58]	Service environments are rarely dry	Controlled RH; long immersion with fluid uptake measured	Medium
Material and application coverage	Insufficient: no study identified for PEKK or seals; one for PA11 [137]; PP only in one abrasion comparison [50]; no neat HDPE dry-sliding baseline; LDPE in one preliminary FFF study [151]; UHMWPE in specialised L-PBF, textured and blend studies [60,150,314]	Common bearing and sealing polymers lack baselines	Baseline tests against moulded equivalents; printed seals with leakage and wear	Medium
Recycled and bio-based feedstocks	Probable (material-specific); single studies per system without comparable replication [17,79,246,327]	Sustainability claims need durability evidence	Wear as a function of reprocessing cycles at fixed test conditions	Medium

Counts refer to the 245 full-text experimental studies of the present evidence base (Section 11). Evidence grades follow Section 2.5 and Section 18.1; “reporting gap” marks rows that describe reporting practice rather than a graded claim. Priority reflects the effect of the gap on design prediction.

## Data Availability

The data extracted for this review, including the study-level appraisal of reporting and evidential completeness, are provided in the Supplementary Materials.

## Supplementary Materials

The following supporting information can be downloaded at: https://www.mdpi.com/article/10.3390/polym18212747/s1, Table S1: Representative tribological studies on printed unfilled thermoplastics (full-text studies with quantitative data); Table S2: Wear of printed photopolymers in engineering and dental tests; Table S3: Representative tribological studies on printed polymer composites (full-text data only); Table S4: Study-level appraisal of reporting and evidential completeness of the 245 full-text experimental studies analysed (Section 2.4); Table S5: Coverage and representativeness of the evidence base (Section 2.2); Table S6: Established findings: scope and sensitivity to the grading rule (Section 2.5 and Section 18.1); Table S7: Robustness of within-study ratio summaries (Section 16.3) to non-independence of ratios, study appraisal and selection; Table S8: Specific wear rates of printed neat PEEK (Dataset S2) by contact class, environment and specimen condition (Section 16.2); Table S9: Extended version of Table 3: reported effects of AM process parameters on friction and wear, with material classes, magnitudes and all supporting studies; Dataset S1: within-study effect sizes (161 ratios from 78 studies; CSV); Dataset S2: specific wear rates of printed specimens (179 values from 59 studies; CSV); Evidence workbook (Microsoft Excel), with sheets listing the 355 records of the evidence base (342 of them in the reference list) with bibliographic data, category, manuscript sections supported, study type and data depth; the evidence matrix (abstract-level extraction for all included studies: material, process, investigated variables, test conditions, COF, wear, principal findings and mechanism); the full-text extraction for 250 records (printed body, grade, print parameters, orientation triplet, contact conditions, environment, roughness of both surfaces, specific wear rate, replicates, dispersion, bulk state, flags and their resolution); the printed-versus-conventional comparisons with matching class and direction; and the screening log with record counts and the decision for every record assessed. The completed PRISMA 2020 checklist is also provided. References [332,333,334,335,336,337,338,339,340,341,342,343] are cited in the Supplementary Materials.

## Abbreviations

The following abbreviations are used in this manuscript:

Abbreviation	Meaning
ABS	Acrylonitrile–butadiene–styrene
AM	Additive manufacturing
ANOVA	Analysis of variance
ASA	Acrylonitrile–styrene–acrylate
ASTM	ASTM International (American Society for Testing and Materials)
ATSP	Aromatic thermosetting copolyester
C&B	Crown and bridge
CF	Carbon fibre
CI	Confidence interval
CIP	Cold isostatic pressing
CNT	Carbon nanotube
CoCr	Cobalt–chromium alloy
COF	Coefficient of friction
CT, µCT	Computed tomography; micro-computed tomography
DC	Degree of conversion
DIW	Direct ink writing
DLP	Digital light processing
DSC	Differential scanning calorimetry
EDS	Energy-dispersive X-ray spectroscopy
EG	Expanded graphite
FDM	Fused deposition modelling
FFF	Fused filament fabrication
FTIR	Fourier-transform infrared spectroscopy
GB	Glass beads
GF	Glass fibre
GNP	Graphene nanoplatelet
hBN, h-BN	Hexagonal boron nitride
HDPE	High-density polyethylene
HDPE-g-SMA	High-density polyethylene grafted with maleic anhydride (authors’ designation)
HIP	Hot isostatic pressing
HIPS	High-impact polystyrene
HIT	Indentation hardness
HT	High-temperature (as in HT-PLA)
HV, VH	Vickers hardness
IR	Infrared
ISO	International Organisation for Standardisation
LCD	Liquid-crystal display (masked) vat photopolymerisation
LDPE	Low-density polyethylene
LED	Light-emitting diode
LPBF, L-PBF	Laser powder bed fusion
LPLA	Graphite-filled (“lubricated”) PLA
MAPE	Mean absolute percentage error
MEX	Material extrusion
MJF	Multi Jet Fusion
MJT	Material jetting
ML	Machine learning
MSLA	Masked stereolithography
n.m.	Not measured
n.r.	Not reported
n.s.	Not significant
PA	Polyamide (PA6, PA6.6/PA66, PA11, PA12, PA1212)
PAO	Poly-α-olefin
PBF	Powder bed fusion
PC	Polycarbonate
PDMS	Polydimethylsiloxane
PE	Polyethylene
PEBA	Polyether block amide
PEEK	Poly(ether ether ketone)
PEI	Polyetherimide
PEKK	Poly(ether ketone ketone)
PET	Poly(ethylene terephthalate)
PETG	Glycol-modified poly(ethylene terephthalate)
PFPE	Perfluoropolyether
PLA	Poly(lactic acid)
PMMA	Poly(methyl methacrylate)
POSS	Polyhedral oligomeric silsesquioxane
PP	Polypropylene
PPS	Poly(phenylene sulfide)
PRISMA	Preferred Reporting Items for Systematic Reviews and Meta-Analyses
PTFE	Polytetrafluoroethylene
PU	Polyurethane
pv	Product of contact pressure and sliding velocity
pvT	Pressure–volume–temperature (moulding device)
Ra	Arithmetic mean roughness of a profile
RH	Relative humidity
RMS	Root mean square
RQ	Research question
Rz	Maximum height of a profile
Sa	Arithmetic mean height of a surface
SCF	Short carbon fibre
SD	Standard deviation
SEM	Scanning electron microscopy
SLA	Stereolithography
SLS	Selective laser sintering
Sq	Root-mean-square height of a surface
Ssk	Skewness of a surface
Sv	Maximum pit height of a surface
SWR	Specific wear rate
Sz	Maximum height of a surface
Tg	Glass transition temperature
TPMS	Triply periodic minimal surface
TPU	Thermoplastic polyurethane
UHMWPE	Ultra-high-molecular-weight polyethylene
UTS	Ultimate tensile strength
UV	Ultraviolet
VPP	Vat photopolymerisation
Xc	Degree of crystallinity
XPS	X-ray photoelectron spectroscopy
ZIF-8	Zeolitic imidazolate framework-8
