# Supplementary material for: A Review of the Tribology of 3D-Printed Polymers and Polymer Composites
Source: Polymers (Basel). 2026 Nov 4;18(21):2747. doi: 10.3390/polym18212747 (PMC13766042; doi:10.3390/polym18212747)
Supplement: Supplementary file 1 [file polymers-18-02747-s001.zip › 01_Supplementary_Tables_S1-S9_R1.pdf]

Supplementary Materials

Supplementary material for "A Review of the Tribology of 3D-Printed Polymers and Polymer Composites". Reference numbers refer to the reference list of the main article; references cited only here are included in that list.

Table S1. Representative tribological studies on printed unfilled thermoplastics (full-text studies with quantitative data).

Polymer	Process (printer)	Key print parameters / orientation	Test configuration	Counterface	Load; velocity	COF	Wear / SWR (as reported, unit)	Dominant mechanism	Ref.
PLA; ABS	FFF (Dremel 3D45)	0.2 mm, 100%; X, Y, Z prints (Y: layers ⊥ sliding; X: layers ∥ sliding)	Linear reciprocating ball-on-plate, ≥3500 m	SS hemispherical probe, 25.4 mm	50 N; 50.8 mm/s	PLA 0.231–0.282; ABS 0.204–0.235	Steady SWR PLA 3.60×10^{-5} (Y) – 2.2×10^{-4} (X); ABS 2.05×10^{-4} – 2.61×10^{-4} mm^3/(N·m)	Adhesion, layer separation	[125]
PLA; ABS	FFF (Wanhao Duplicator 4s plus)	1 mm layers (as stated), 20% infill; sliding along (L.D) or across (T.D) the deposited lines	Linear reciprocating pin-on-plate (ASTM G133)	CoCr pin Ø5 mm, 260 HB	50–100 N; 0.5–1 mm/s	PLA 0.228–0.294; ABS 0.297–0.44 (T.D > L.D in all conditions); conventional UHMWPE 0.109–0.119	Not measured	Roughness-controlled friction (proposed; T.D Ra 2.0–9.5× L.D, derived)	[130]
PETG; PLA; PLA+Al; ABS; TPU 93A/82A; SLA resins	FFF (Flashforge Creator Pro); SLA	0.12–0.2 mm (FFF), 0.05 mm (SLA), 100% infill; bed-contact face; two orthogonal directions	Linear sliding of a steel disc over the printed block, 5–6 s	C45 steel disc Ø66 mm	50 and 150 N; 0.1 and 1 mm/s	ABS 0.056–0.117 (lowest); TPU 82A 0.207–0.326 (highest); resins 0.105–0.131	Not measured	Not analysed; COF fell with speed for most thermoplastics, not for resins	[318]
PLA	FFF (Bambu Lab A1)	0.2 mm, 100%, 45° raster; annealed 90–130 °C, 60–180 min	Pin-on-disc (ASTM G99)	n.r.	n.r.; n.r.	0.47 (as-printed) → 0.16 (110 °C/60 min)	7.52×10^{-3} → 1.14×10^{-3} mm^3/(N·m)	Abrasion + adhesion → mild adhesion; embrittlement after long anneals	[127]
PLA; PEEK	FFF (Prusa CORE One; INTAMSYS FUNMAT HT)	0.2 mm, solid; annealed (PLA 95 °C/1 h; PEEK stepped to 220 °C)	Pin-on-disc, 600–900 min	SS disc ≈910 HV, ≈220 nm	PLA 5 N, PEEK 25 N; 120 rpm	Graphical only	Solid pins: PLA 1.5×10^{-4} → 2.74×10^{-4}; PEEK 6.2×10^{-6} → 1.74×10^{-6} mm^3/(N·m)	PLA: brittle powder debris, unstable film; PEEK: faster stable transfer film	[41]
PLA	FFF (Elegoo Centauri Carbon)	0.2 mm, ±45°, no top skin; infill 20–80%, three patterns	Pin-on-disc, two-body abrasion, 180 m	SiC paper (600 mesh)	10 N; 2 m/s	0.431–0.557	17.97 mm^3 (80% rectilinear) ≈ 1.0×10^{-2} mm^3/(N·m) (derived)	Abrasion, inter-road opening, pull-out	[246]
PLA; HT-PLA; PETG	FFF (Bq Witbox 2)	0.2 mm, solid; horizontal (X) vs vertical (Z) cylinders	Reciprocating cylinder-on-plate (line contact)	Ground steel, Ra 1.07 µm	150 N; ≈0.12 m/s (derived)	Dynamic 0.31–0.44	Wear volume 0.1365 mm^3 (HT-PLA, X) – 0.594 mm^3 (HT-PLA, Z)	Groove-edge contact, stick–slip	[126]
PLA; PETG; ABS	FFF (Bambu Lab P2S)	0.2 mm, 100%, printed flat	Pin-on-disc	C45 steel, 60 HRC, Ra 0.16 µm	14 N; ≈1 m/s	ABS ≈0.45; PETG rising to 0.55	Mass loss PLA 0.1277, PETG 0.0051, ABS 0.0056 g	PLA catastrophic (3.1 mm height loss)	[65]
ABS; PLA; HIPS	FFF (Anycubic Mega S)	0.1–0.3 mm, 100%, 45° raster	Ball-on-disc (ASTM G99), 150/300 m	Ball (material n.r.)	20 N; 1 m/s	n.r.	Wear volume ABS <0.15, PLA ≈1.5, HIPS ≈3.0 mm^3	ABS plastic deformation; PLA/HIPS material loss	[122]

Polymer	Process (printer)	Key print parameters / orientation	Test configuration	Counterface	Load; velocity	COF	Wear / SWR (as reported, unit)	Dominant mechanism	Ref.
ABS (PET/PC-modified)	FFF (UltiMaker 2+)	0.1 mm; layer lines 0°, ±45°, 90° to sliding	Flat pin-on-disc, 120 s	6082 Al alloy disc	10–20 N; 0.314–0.628 m/s (coupled)	Graphical; 90° > 0° > ±45°	Wear ranking 0° > −45° > 45° > 90°; measured depths 95–160 μm (0°, ±45°)	0°: interlayer shear, delamination; 90°: ploughing of ridges	[32]
ABS	FFF, heated chamber (D-Force D300-HC)	0.2 mm, 100%; 0/90° or ±45°; 20/50 mm/s	Reciprocating flat-on-flat (TE77), 432 m	SS316, Sa 0.25 μm	80/160 N; 0.02 m/s	0.18–0.23	≤6.2×10^{-5} mm^3/(N·m)	Void-initiated subsurface cracking, bead separation	[64]
ASA	FFF (Anet E12)	0.15–0.35 mm; 240–260 °C	Ball-on-disc (ASTM G99), 250 m	AISI 304 ball, Ø3 mm	10 N; 0.10 m/s	Graphical; ≈65% higher at 0.35 than at 0.15 mm	Track depth up to ≈120 μm	Abrasion, third-body debris	[131]
PLA; PETG; HIPS; PA; ABS; ASA	FFF (Prusa i3 MK3S)	0.3 mm, 100%, three orientations	Two-body abrasion on abrasive belt, 12 m	SiC belt, 180–212 μm grit	250 kPa; 0.2 m/s	n.r. (friction force 7.2–10.3 N)	Mass loss ABS >0.3, HIPS 0.19, ASA 0.18, PETG and PA ≈0.1, PLA ≤0.1 g	Two-body abrasion	[129]
PLA; PETG; ABS; PA12; PC; PMMA; HIPS; PP	FFF (Zortrax M300 Dual)	0.2 mm, 100%, 45° raster	Three-body rubber-wheel abrasion, 600 rev	Rubber wheel + Al_2O_3 (125–180 μm)	44 N; 60 rpm	n.r.	Extruded volume loss lower than printed by 9.6–21.6% (ABS, PC, PMMA, PETG, PLA); higher by 16.3% (HIPS) and 34.1% (PP)	Delamination of deposited paths	[50]
PETG	FFF (Flashforge Dreamer)	0.1 mm, 6 perimeters, vertical pins, polished; air vs N_2; annealed	Pin-on-disc, 720 m	440C (HRC 20), ground	1 MPa ≈ 31.7 N (derived); 0.20 m/s	0.36–0.54	3.4×10^{-5} (air), 5.5×10^{-5} (N_2), 1.5×10^{-5} (annealed) mm^3/(N·m)	Oxidation-assisted thick transfer film	[51]
PA	FFF (Ultimaker S5)	240–260 °C; 0.1–0.3 mm; polished and as-printed	Reciprocating ball-on-flat, 1 h	GCr15 ball, Ø6 mm	5–20 N; 1 Hz, 5 mm stroke	Polished 0.50–0.60; as-printed 0.05–0.06	Minimum 1.75×10^{-5} mm^3/(N·m) (260 °C, 5 N)	Peeling/delamination at low nozzle temperature; adhesion	[39]
PA (Nylon Bridge)	FFF (Ender 3, enclosed)	0.2 mm; 0°, 45°, 90°, 0/90°, 45/135° rasters	Dry sand–rubber wheel (ASTM G65), 10 min	Rubber wheel + SiO_2 (212–300 μm)	45–130 N; 200 rpm	n.r.	Mass loss 45/135° vs 90°: +52%, +31%, +16% at 45, 85, 130 N	Micro-ploughing → micro-cutting; layer-by-layer detachment	[133]
PA (Nylon 618, 645, Alloy 910)	FFF (Ultimaker 2)	Default settings, 60% infill	Back-to-back spur gears, self-mated	Printed gear	5–15 N·m; 1000 rpm	n.r.	Nylon 618: 2.4×10^6 cycles (5 N·m, run-out) to 1.2×10^4 (15 N·m); other grades <2×10^4 cycles at 5 N·m	Thermal softening; interlayer peeling in lower-crystallinity grades	[4]
PA12	SLS (EOS Formiga P100)	PA2200, virgin powder	Unidirectional ball-on-flat	SS ball, Ø6.35 mm	10/30 N; 0.35 m/s	0.305/0.261	4.78×10^{-4}/3.20×10^{-4} mm^3/(N·m)	Adhesive debris; no transfer film	[134]
PA11	SLS (EOS P110)	Energy density 0.030 and 0.050 J/mm^2	Ball-on-disc (ASTM G99), 140 m	Al_2O_3 ball	3 N; 0.07 m/s	n.r.	6.67×10^{-4} (0.030) and 5.10×10^{-4} (0.050 J/mm^2) mm^3/(N·m)	Abrasion + fatigue; void-initiated microcracks	[137]

Polymer	Process (printer)	Key print parameters / orientation	Test configuration	Counterface	Load; velocity	COF	Wear / SWR (as reported, unit)	Dominant mechanism	Ref.
PEEK	FFF (Yimai MAGIC-HT-PRO)	L9: 410–430 °C, 0.1–0.3 mm, raster angle, speed; one set annealed 250 °C/24 h	Reciprocating ball-on-block, 72 m	GCr15 ball Ø6 mm, Ra ≤0.02 μm	70/90 N; ≈0.02 m/s (derived)	0.27–0.33 (70 N); 0.37 → 0.24 annealed (90 N)	7.31–10.37$\times 10^{-5}$ (70 N); 11.83 → ≈4.80$\times 10^{-5}$ annealed (90 N) mm^3/(N·m)	Adhesion, abrasion, fatigue	[139]
PEEK	FFF (IEMAI MAGIC-HT-PRO)	430 °C, 0.15 mm, 0/90°; annealed 150–190 °C/0.5 h	Reciprocating ball-on-flat, 1800 s	GCr15 ball, Ø6 mm	30 N; ≈0.01 m/s (derived)	Graphical (rises with annealing temperature)	2.83$\times 10^{-5}$ mm^3/(N·m) after 190 °C anneal (−34%)	Abrasion + adhesion → adhesion; fatigue cracks (unannealed, 70 N)	[138]
PEEK	FFF (MAGIC-HT-L)	440 °C, 0.2 mm; filaments ‖, ⊥, 45° to sliding; annealed 160–300 °C	Block-on-ring, 120 min	GCr15 ring, Ra 0.2 μm	1 MPa ≈ 16 N (derived); ≈1.01 m/s (derived)	Graphical	Minimum 1.37$\times 10^{-6}$ mm^3/(N·m) (200 °C anneal, ‖)	Transfer film filling grooves	[140]
PEEK	FFF (Hyrel Hydra 16A)	0.2 mm, 0/90°; infill 40–90%	Pin-on-disc, 600 m	SUJ2 steel disc	20 N; 0.2 m/s	Graphical (40% lowest)	7.4$\times 10^{-5}$ (80%) – 12.8$\times 10^{-5}$ (40%) mm^3/(N·m)	Delamination, smearing; abrasion at 40%	[34]
PEEK	Polymer PBF (TPM3D S320HT)	25 W, 0.12 mm layer; three faces	Reciprocating ball-on-plate	440C ball, Ø5 mm	5 N; 5 mm/s	≈0.37	3.7$\times 10^{-5}$ mm^3/(N·m) (median)	Adhesion, plastic flow, fatigue delamination	[6]
PEEK (implant grade)	FFF (Apium P220)	0.1 mm, 0/90°, horizontal build	Pin-on-disc in serum at 37 °C, 1000 m	AISI 52100 ball-ended pin	14–70 N; ≈50 mm/s	0.133–0.213	1.66$\times 10^{-5}$ – 2.65$\times 10^{-4}$ mm^3/(N·m) (derived)	Mild → severe (plastic deformation, cracking) between 30 and 50 N	[49]
PEI (ULTEM 9085)	FFF (Fortus 400mc)	0.254 mm; X vs Z build; 0°, 45°, 90° and alternating paths	Dry sand–rubber wheel (ASTM G65), 200 m	Rubber wheel + garnet	30 N; ≈3.3 m/s (derived)	n.r.	Mass loss 0.0631–0.134 g; Z total +26.91% vs X	Three-body abrasion	[144]
TPU 82A	FFF (Creality K1C)	0.2 mm; gyroid 10/50/100%	Ball-on-disc, 300 m	100Cr6 ball, Ø6 mm	5 N; 0.15 m/s	0.504/1.002/0.465	No measurable mass loss	Viscoelastic deformation; adhesion at 50% infill	[107]
TPU 95A	FFF (Ultimaker S5)	0.1 mm; 230 °C	Reciprocating ball-on-flat, 10,000 s	SS ball, Ø6.2 mm	5–20 N; 0.5–1.5 Hz	Graphical	2.08$\times 10^{-6}$ – 6.56$\times 10^{-6}$ mm^3/(N·m)	Viscous and fatigue wear	[146]
TPU 60A; TPU 95A	FFF (Artillery Sidewinder X4 Plus)	Infill 70/100%	Pin-on-disc, 200 m	Cardboard	0.5 N; 0.1 m/s	60A 1.233–1.325; 95A 0.195–0.436	Mass loss 60A 0.5–0.9 mg; 95A 0.1 mg	Infill stiffness → contact pressure	[147]
TPU 92A; PA12	SLS (DTM Sinterstation 2500 plus)	Horizontal, vertical-x, vertical-y builds	Ball and blade abrasion, 8 h	n.r.	45 N; 56.55 mm/s	TPU 0.45 (25 °C) → 0.55 (35 °C); PA12 <0.3	Graphical	TPU: ≈250 μm interlayer delamination plates; PA12: abrasion	[86]
UHMWPE	Polymer L-PBF (ProMaker P2000 HT) + HT/CIP/HIP	Relative density ≈61–90%	Unidirectional pin-on-disc	SS disc, Sa 1.14 μm	1 MPa; ≈25 mm/s (derived)	n.r.	Linear wear 1.86$\times 10^{-2}$ – 1.86$\times 10^{-3}$ μm/m ≈ 1.86$\times 10^{-5}$ – 1.86$\times 10^{-6}$ mm^3/(N·m) (derived)	Pore-initiated subsurface cracking	[60]

Polymer	Process (printer)	Key print parameters / orientation	Test configuration	Counterface	Load; velocity	COF	Wear / SWR (as reported, unit)	Dominant mechanism	Ref.
UHMWPE	FFF (Hori Z1000), in-house filament	Wedge textures 100–250 μm deep	Reciprocating pin-on-plate in 25% bovine serum, 10,000 cycles	Polished steel pin, Ra 0.5 μm	40 N; 1 Hz, 6 mm stroke	0.075–0.108	Minimum 0.510×10^{-6} $mm^3/(N\cdot m)$ (200 μm texture)	Abrasion + fatigue	[150]
PC	FFF (Bambu Lab X1 Carbon)	0.2 mm, 100%; 0°, 45°, 90° (both bodies)	Reciprocating pin-on-plate, self-mated, dry and greased, 50 m	Printed PC plate	39.24/58.86 N; 15–20 mm/s	90°: 0.044–0.096	Not measured (90° pin Ra 0.80 → 0.24 μm)	90°: plastic deformation; 0°: adhesion, abrasion	[61]
PPS	FFF (printer n.r.)	0.2 mm, ±45°, 100%	Block-on-ring	Carbon steel ring, HRC 40–50, Ra 0.3–0.4 μm	100/196 N; ≈0.42 m/s (derived)	Graphical	3.048×10^{-5} (100 N); 1.14×10^{-3} (196 N) $mm^3/(N\cdot m)$	Adhesion; thermal softening at 196 N	[153]

Note: SWR, specific wear rate; COF, coefficient of friction; n.r., not reported; SS, stainless steel; FFF, fused filament fabrication; SLS, selective laser sintering; PBF, powder bed fusion; L-PBF, laser powder bed fusion; MJF, Multi Jet Fusion; HT/CIP/HIP, heat treatment, cold and hot isostatic pressing. "Graphical" means values given only in figures. "(derived)" marks loads, velocities or SWR computed by the authors of this review from reported geometry, pressure, volume or linear wear rate (e.g. SWR = linear wear rate/nominal pressure for [60]). Values are as reported in each study and are not comparable across rows because test conditions differ (Section 16).

Table S2. Wear of printed photopolymers in engineering and dental tests.

Resin (type)	Process	Build angle / layer / post-cure	Test (simulator or rig, cycles, load)	Antagonist / counterface	Wear metric and value (printed)	Comparator and value	Outcome	Ref.
Engineering resins								
Digital ABS Plus, VeroGray (rigid acrylates)	MJ (PolyJet)	Flat; 300×300×1600 dpi; glossy finish	Reciprocating ball-on-plate, 2 mm stroke, 8 mm/s, 60 min, 1/5/10 N, dry	AISI 52100 ball Ø6.5 mm, Ra 0.07 μm	Steady COF 0.76 (VeroGray, 5 N, ∥ raster) to 1.20 (ABS, 1 N, ⊥ raster); SWR graphical only	Sliding ∥ vs ⊥ raster	⊥ raster raised COF at 1 N only; at 5–10 N COF lower and orientation-independent; wear ranking of the two resins reversed with load	[35]
Polyurethane-acrylate (rigid)	DLP	0–75° / 50 μm / UV 5 min; as-printed vs 2000-grit polished	Reciprocating ball-on-flat, 2 mm stroke, 5000–10 000 cycles, 0.1–0.2 N, dry	Stainless-steel ball Ø1 mm	As-printed COF 0.53–0.65 (Ra 0.51–10.26 μm); polished 75°, 0.2 N: COF 0.433, SWR 3.78×10^{-5} $mm^3/(N{\cdot}m)$ (derived, unit conversion)	Polished 0°, 0.2 N: COF 0.572	Angle acted through stair-step topography; after polishing 75° gave lowest COF (−24%, derived) and wear	[88]
Polyurethane-acrylate + 0–2 wt% GNP	DLP	Horizontal/45°/vertical / 35, 50, 100 μm / 60 °C 30 min + UV 30 min	Reciprocating cylinder-on-plate (ASTM G133-type), 6 mm, 4.583 Hz, 60 min, 150 N, dry	Ground S355 steel, Ra 0.10–0.12 μm	Neat dynamic COF 0.6–0.7; depth and SWR graphical	Neat resin, same build	0.5 wt% GNP: COF ≈−50%, wear depth −27.2% (50 μm) and −31% (35 μm) in horizontal builds, but +98% (100 μm) and +64% (50 μm) in 45°/vertical builds	[89]
ABS-like, high-tough and C&B resins	MSLA	Vertical pins / 50 μm / wash E70, E95 or IPA; UV 3, 5, 10 min	Pin-on-disc (ASTM G99), 100 rpm, 300 s, 20 N, dry	Disc Ø46 mm, material n.r.	Mass loss 0.35–1.16%; COF 0.188–0.272	3^3 factorial, n = 3	Resin type explained 73.57% (mass loss) and 57.80% (COF) of variation; wash and cure ≤2.29%; hardest resin did not wear least	[120]
Vero rigid skin over Agilus elastomer (multi-material)	MJ (PolyJet)	Z-stacked; rigid 1.0–1.5 mm over soft 0.4–0.8 mm; lightly polished	Reciprocating ball-on-flat, 6 mm, 1 Hz, 600 s, 5–40 N (wear at 20 N), dry, 35–45% RH	GCr15 ball Ø6.3 mm	COF 0.52–0.70; SWR 4.9674×10^{-4}–1.0458×10^{-3} $mm^3/(N{\cdot}m)$	Monolithic rigid resin: COF ≈0.70, SWR 1.3292×10^{-3} $mm^3/(N{\cdot}m)$	Best layup (1.5/0.4 mm): COF −25.7%, SWR −62.6%	[94]
Photosensitive resin + 0–5 wt% polysiloxane core–shell particles	VPP	n.r. (supplementary)	Reciprocating, 2.5 mm/s, 2000 s, 80 N, dry; scratch 5–30 N	n.r. in main text	2.5 wt%: COF ≈0.15; wear volume 0.326 mm^3	Neat: COF ≈0.67; 1.345 mm^3	Optimum at 2.5 wt% (−75.8% volume); 5 wt% agglomerated (0.774 mm^3)	[155]
Acrylate + 5–30 wt% PFPE-loaded mesoporous silica	DLP	100 μm; IPA wipe; polished before test	Reciprocating ball-on-flat, 1.38 mm, 3 Hz, 1800 s, 5–20 N (main 10 N), dry	GCr15 ball Ø6 mm	20 wt%: COF ≈0.1; wear volume 0.003 mm^3	Neat: COF ≈0.7; oil-free silica 0.23, unstable after 1200 s	Wear rate −97.7%; 30 wt% no further gain, less stable COF	[154]

Resin (type)	Process	Build angle / layer / post-cure	Test (simulator or rig, cycles, load)	Antagonist / counterface	Wear metric and value (printed)	Comparator and value	Outcome	Ref.
Acrylate/cyanate ester + talc 0–10 wt% + PAO-40 0–7 wt%	DLP + thermal cure	50–200 μm; stepwise 140–220 °C, 8 h	Reciprocating ball-on-flat, 5 mm, 2 Hz, 60 min, 5 N, dry	GCr15 ball Ø6 mm	10 wt% talc + 7 wt% PAO: COF 0.069; 1.12×10^6 μm³	Pure CE: COF 0.404, 8.17×10^6 μm³; 10 wt% talc only: 35.68×10^6 μm³	Oil lowered COF 83% and wear volume 86% vs pure CE (derived); talc alone raised wear	[157]
ABS-like resin TPMS lattices (20–40% porosity), silicone-oil impregnated	VPP	50 μm; UV 3 min per face; lightly sanded	Reciprocating ball-on-flat, 8 mm, 3 Hz, 30 min, 5–10 N	440C ball Ø8 mm	Oil-impregnated: COF 0.02158–0.06; wear depth 0.034–0.110 mm	Same lattices dry: depth 0.068–0.253 mm	Depth −50 to −70% with oil; optimum porosity 25–35%	[56]
VisiJet M3 Crystal acrylate, printed textures	MJP	Concave/convex dimples, 0.2 mm wide and spaced, 0.15 mm deep	Reciprocating ball-on-plate, 10 000 cycles, 3 Hz, 1 and 10 N, dry	SUJ2 ball Ø5 mm	10 N height loss: square-concave 0.030, circle-concave 0.072, circle-convex 0.165 mm	Untextured 0.156 mm	Wear volume −78 to −97% (derived) at 10 N; no texture benefit at 1 N	[275]
Dental: crowns and interim restorations								
Micro-filled C&B methacrylate	DLP	0/45/90° / 50, 100 μm / none or 5–30 min LED vs bulb	CS-4.8, 60 000 cycles, 5 kg, water + TC	Zirconia hemisphere r 1.5 mm	Volume 0.706–1.208 mm³ (post-cured); 1.872 mm³ (no post-cure)	Within-study	45° −21% vs 0°/90° (derived); layer n.s.; no post-cure +93% (derived)	[167]
C&B methacrylate (interim)	DLP	0° / 100 μm / 30 min; polished	CS-4.8, 30 000 and 60 000 cycles, 5 kg, water + TC	LPBF CoCr hemisphere r 1.5 mm	0.08 (30k) and 0.10 mm³ (60k)	Milled PMMA 0.06/0.21; self-cured PMMA 0.11/0.44 mm³	Not lower at 30k; −52% vs milled and −77% vs self-cured at 60k (derived)	[179]
Interim resins: filled (50 wt%) vs two low-filler	DLP	∥ platform / n.r. / N_2 flash; ground	ACTA three-body, 200 000 cycles, 15 N, millet slurry	Metal wheel + slurry	Depth 62 μm (filled); 255–257 μm (low-filler)	Milled PMMA 236 μm; direct composite 50 μm	Filled −76% (derived), grouped with composite; low-filler grouped with milled PMMA	[168]
Definitive crown resins (≤50 wt% filler)	DLP	0° / manufacturer / N_2 flash; ground	ACTA three-body, 200 000 cycles, 15 N, millet slurry	Metal wheel + slurry	Depth 75.50–150.54 μm	Milled composite (86 wt%/73 vol% filler) 26.42 μm	2.9–5.7× deeper (derived); two-body ranking differed (units ambiguous)	[173]
Crown composites, 10–55 wt% filler	DLP	n.r. / n.r. / LED-UV; polished	CS-4, 600 000 cycles, 50 N	Steel sphere Ø3 mm	Volume 0.7–1.7 mm³; vertical 310–546 μm	Milled nanoceramic composite 0.7 mm³; 215.6 μm	10 wt% resin equal in volume; 55 wt% resin 2.4× volume (derived) and most cracking despite 96.5% DC	[93]
Definitive ceramic-filled resin (≈50%)	DLP	∥ plate / 100 μm / ProCure 2	Alabama device, 400 000 cycles, 20 N, 33% glycerin	Human enamel cusps	Material 0.07 mm³; enamel loss 0.95 mm³	Lithium disilicate 0.46 mm³; enamel 1.81 mm³	Material −85% and enamel −48% (derived); not material-matched	[185]
C&B interim resin, crowns	DLP	0/45/90° / 50 μm / 300 s; polished	In vivo crossover, 1 week per crown, n = 5	Natural antagonists	Volume 1.22 (0°), 1.32 (45°), 1.74 mm³ (90°)	Chairside self-cured resin 0.70 mm³	Only 90° differed (+149%, derived); 90° vs 0° +43% (derived)	[9]

Resin (type)	Process	Build angle / layer / post-cure	Test (simulator or rig, cycles, load)	Antagonist / counterface	Wear metric and value (printed)	Comparator and value	Outcome	Ref.
Ceramic-filled hybrid (30–50 wt%), definitive	VPP (Varseo XS)	35–45° / 50 µm / 2×1500 flashes; glazed	Clinical, 352 restorations, 12 and 24 months	Natural dentition	24-month maximum height loss 0.76 mm (molars 1.25 mm)	None (0.1 mm hypothesis)	35% exceeded 0.5 mm by 24 months; molar > premolar > anterior	[19]
Dental: fixed dental prostheses								
Definitive resin, three-unit FDPs	DLP, LCD, LFD	45° / 50 µm / 60 °C 2×20 min; unpolished	MOD simulator, 1.2×10^6 cycles, 50 N, water + TC	Steel sphere Ø6 mm	Volume 1.99 (DLP), 2.78 (LCD), 2.62 mm^3 (LFD)	Milled HIPC 0.80 mm^3 (different material)	Printer type changed volume by up to 40% (derived); comparison not material-matched	[183]
Dental: occlusal devices								
Splint acrylate	DLP	90° / 50, 75, 100 µm / N_2 flash; half polished	CS-4, 120 000 cycles, 50 N, water	Steel ball	Volume 0.046–0.123 mm^3	Within-study	Polishing −38% (derived; p = 0.003); 50→100 µm +50% (derived), n.s.	[166]
Splint resins (2 DLP, 1 SLA)	DLP, low-force SLA	0/45/90° / 50, 100 µm / per manufacturer; polished	CS-4.8, 80 000 cycles, 50 N, water + TC	Steatite ball Ø6 mm	Volume 18.54–28.71 mm^3 (DLP); 79.96–95.60 mm^3 (SLA, depth-capped)	Milled PMMA 12.35 mm^3	Printed 1.5–7.7× milled (derived); orientation significant overall, not within materials	[193]
Four splint methacrylates	DLP-type	0/45/90° / 100 µm / n.r.; polished	Block-on-ring (mod. ASTM G77), 12-month equivalent, 49 N, artificial saliva 37 °C	Human enamel blocks	Volume 25.36–204.59 mm^3; vertical 231–545 µm	Milled PMMA 6.44 mm^3/48.3 µm; heat-cured 17.22 mm^3/148 µm	3.9–32× milled volume (derived); angle effects material-specific	[181]
Rigid (2) and flexible (3) splint methacrylates	VPP	Face ∥ plate / 100 µm / per manufacturer	Modified Alabama tester, 400 000 cycles, 20 N, 33% glycerin	Zirconia ball Ø4.76 mm	Rigid 0.011–0.028 mm^3; flexible 0.250–0.289 mm^3	Milled 0.041, heat-polymerised 0.040, light-polymerised 0.013 mm^3	Rigid equivalent to conventional; flexible 6.1–7.0× milled (derived); wear–hardness r = −0.93 across all ten materials (two clusters; Section 14.1)	[11]
Splint resins (SLA, LFS, DLP)	SLA, LFS, DLP	Horizontal / n.r. / per manufacturer; polished	CS-4.8, 200 000 cycles at 20 N then 50 N, water + TC	Human premolar enamel cusps	50 N volume 2.23–3.59 mm^3	Injection-moulded PMMA 2.81; milled PMMA 2.38 mm^3	No significant differences in material or antagonist wear	[10]
Dental: denture teeth								
Filled C&B MFH (DLP) vs unfilled teeth resin (SLA)	DLP, SLA	0/45/90° / 50 µm / per manufacturer; polished; TC	CS-4.4, 120 000 cycles, 68.6 N, water	Zirconia hemisphere r 2 mm	Volume DLP 0.023–0.059 mm^3 (SD > mean); SLA 1.325–1.440 mm^3	Prefabricated IPN PMMA 0.798 mm^3	SLA +66 to +80% vs control (derived); resin dominated; orientation P = .049, no pairwise differences	[165]
Filled hybrid, DUDMA methacrylate, nano-ceramic hybrid	DLP	n.r. / 100 µm / glycerin post-cure	CS-4 (ISO/TS 14569-2), 250 000 cycles, 49 N, TC	Steatite ball Ø6 mm	Volume 0.582, 1.351, 1.841 mm^3	Prefabricated DCL PMMA 0.427 mm^3	Filled hybrid n.s. vs PMMA; others 3.2× and 4.3× (derived)	[169]

Resin (type)	Process	Build angle / layer / post-cure	Test (simulator or rig, cycles, load)	Antagonist / counterface	Wear metric and value (printed)	Comparator and value	Outcome	Ref.
UDMA denture-tooth resin (~40 wt% filler)	DLP	0° / 50 µm / glycerin light cure, then 121 °C, 2 bar steam 4–90 min; polished	Impact-sliding K655-05, 50 000 cycles, 49 N, water 37 °C + PMMA slurry	Steel hemisphere Ø4 mm	Median volume 0.23 mm^3 (90 min)	Untreated 1.15 mm^3	Volume −80%, depth −51% (derived)	[172]
Dental: toothbrush abrasion								
Permanent crown resins (2)	VPP	Vertical / 50 µm / UV; polished	Toothbrush simulator, 10 000 cycles, 2.0 N, RDA 70 slurry	Toothbrush + dentifrice	Mass loss ≈0.001 (derived; units n.r.); Ra 1.08→1.52 and 0.94→1.58	Milled composite ≈0.003 (derived; different product)	Milligram-level loss; printed resins roughest before and after brushing	[190]

Note: C&B, crown and bridge; CE, cyanate ester; COF, coefficient of friction; CS, chewing simulator (SD Mechatronik model number); DC, degree of conversion; DCL, double-cross-linked; DLP, digital light processing; DUDMA, diurethane dimethacrylate; FDP, fixed dental prosthesis; GNP, graphene nanoplatelets; HIPC, high-impact polymer composite; IPA, isopropyl alcohol; IPN, interpenetrating network; LCD, liquid-crystal display (masked) printing; LFD, low-force display; LFS, low-force stereolithography; LPBF, laser powder bed fusion; MJ, material jetting; MJP, MultiJet printing; MSLA, masked stereolithography; n.r., not reported; n.s., not significant; PAO, poly-α-olefin; PFPE, perfluoropolyether; PMMA, poly(methyl methacrylate); SLA, stereolithography; SWR, specific wear rate; TC, thermocycling (5–55 °C); TPMS, triply periodic minimal surface; UDMA, urethane dimethacrylate; VPP, vat photopolymerisation (technology not specified); ∥/⊥, parallel/perpendicular. "(derived)" marks values calculated for this review from the reported means; percentage changes relative to the comparator in the same row. Absolute values are not comparable between rows because loads, cycles, antagonists and metrics differ (Section 11). DLP standard deviations in [165] exceed the means as published.

Table S3. Representative tribological studies on printed polymer composites (full-text data only). Effects are within-study, relative to the unfilled printed matrix unless stated otherwise.

Matrix	Filler (type, loading)	Process	Orientation / architecture	Test (config, counterface, load, speed, environment)	Effect on COF vs unfilled (within-study)	Effect on wear vs unfilled (within-study)	Dominant mechanism	Ref.
PEEK	SCF 5–15 wt%	PBF	X-Z (fibre ends), Y-Z, X-Y faces	Recip. ball-on-plate, 440C, 5 N, 5 mm/s, dry	0.37 → 0.27 (15 wt%)	SWR 3.7×10^{-5} → 2.0×10^{-6} $mm^3/(N{\cdot}m)$ (≈18×, derived); X-Z −28% vs X-Y (5 wt%)	End-on fibres as micro-pillars; in-plane fibre debonding	[6]
PEEK	SCF 10 + graphite 10 wt%	FFF	P, AP, N fibre triplets	Plate-on-ring, 100Cr6 Ra 0.2 μm, 50–100 N, 1–4 m/s, dry	No unfilled control; N vs P 0.32 vs 0.16	N vs P 0.99 vs 0.24×10^{-6} $mm^3/(N{\cdot}m)$ (≈4.1×, derived)	Fibre thinning (P) vs fibre-end fracture, third bodies (N)	[62]
PEEK	SCF/graphite vs commercial multiscale grade	FFF	P, AP, N, 45°	Plate-on-ring, steel Ra ≈0.2 μm, 50–100 N, 1–4 m/s, dry	COF anisotropy 1.24 → 1.11	SWR anisotropy (max/min) 2.70 → 1.41	Nanoparticles patch fibre fracture sites	[232]
PEEK	SCF 10, 20 wt%	FFF	n.d.	Recip. flat-on-flat, 316L Sa 0.25 μm, 80 N, 0.02 m/s, dry/water	−37% (10 wt%, dry)	Neat SWR ~10× higher in water; CF grades insensitive	Debonding, pull-out; CF abrades transfer film	[53]
PEEK	SCF 5 wt%	FFF	Top/front/side faces	Recip. ball-on-block, 316L, 30 N, ≈0.1 m/s, water	−21 to −40%	SWR ~10× lower	Fibre breakage, fatigue; graphitised debris film	[99]
PEEK	SCF 5 wt% ± laser texture	FFF	Fibres ∥ vs ⊥ sliding (in-plane)	Recip. ball, 316L, 10–30 N, 0.04 m/s, water	Texture −8 to −17% (∥ only)	⊥ 35–40% lower than ∥ (derived); texture −55% (∥), +45–120% (⊥)	Debris fragmentation by ⊥ fibres; groove debris storage	[291]
PA12	CF (commercial)	SLS	Five build orientations	Pin-on-disc, SS Ra 0.36 μm, 50 N, 0.25 m/s, dry	≈−25%	+15–40% wear resistance; Z-built CF-PA12 worse than PA12	Fibre crushing; film peeling by protruding fibres	[202]
PA6	CF ≈11 wt%, desized vs MAH-grafted	Screw extrusion	Sliding ⊥ print direction	Recip. ball, GCr15, 50 N, 0.083 m/s, dry	−29% (grafted vs desized CF)	K −70.5% (SEBS-g-MAH vs SEBS matrix)	Interfacial debonding when weakly bonded	[76]
Nylon	SCF ≈9.6 vol%	FFF	Flat and textured pins	Pin-on-disc, SS Ra ≈0.22 μm, 20–40 N, 0.1 m/s, dry/water	Lower in all conditions	Lower SWR in all conditions	Fibre breakage, third-body debris	[59]
PA6.6	CF 25%, GF 25%	FFF	Top vs bottom surface	Pin-on-disc, ceramic ball, 25 N, ≈0.16 m/s	n.r.	Neat lowest; CF top >2× CF bottom	Fibre peeling, three-body abrasion	[204]
ASA (vs PLA)	Aramid (commercial)	FFF	Infill 30–90%	Ball-on-disc, 100Cr6, 5 N, 0.15 m/s, dry	n.r.	Depth 120–154 μm, ~3× PLA (different matrix)	Abrasion, fibre detachment, ball scratched	[206]
PLA	Milled GF 10, 15 wt%	FFF	±45° raster to sliding	Recip. ball, 100Cr6, 10/20 N, 2 Hz, dry after 0–400 hygrothermal cycles	10 N unaged 0.51 → 0.60–0.64; 1.37 (15GF, 200 cycles, 20 N)	10 N: 10GF ≈10× higher (derived); 20 N: 15GF −78% (derived)	Debonding, pull-out, third-body ploughing	[58]
PLA	Milled GF 10, 15 wt%	FFF	±45° raster	Recip. ball, 100Cr6, 40 N, 0.5/1 Hz, dry after pressurised-water ageing	Not resolved	Depth −23 to −32% unaged; +21–23% after 28 d (derived)	Interface-dominated damage after ageing	[57]
PA6	Continuous CF ≈35 vol%	FFF (continuous fibre)	P, AP, N	Pin-on-disc, steel 880 HV Ra 0.25 μm, 8–32 N, 0.5 m/s, dry	n.r. vs neat	N 2.7–7.8× P (derived); neat PA6 failed at 2 MPa	Fibre-tip cracking (N); fibre thinning (P)	[73]

Matrix	Filler (type, loading)	Process	Orientation / architecture	Test (config, counterface, load, speed, environment)	Effect on COF vs unfilled (within-study)	Effect on wear vs unfilled (within-study)	Dominant mechanism	Ref.
ABS	Continuous CF 6.6–11 vol%	FFF (in-line impregnation)	n.d.	Pin-on-disc, 40HM steel, 1.0 m/s, air vs water	~2× lower in water than air	~10× lower linear wear intensity in water; >9 vol% below neat (dry)	Fibre fusion into softened matrix (dry); cooling, flushing (water)	[208]
PA12	PTFE, graphite, MoS_2 (1:100)	SLS	Top surface, layers ∥ contact	Ball-on-flat, SS, 10/30 N, 0.35 m/s, dry	PTFE −37/−50%; graphite ≈−5%; MoS_2 ≈0	PTFE +12/+43%; MoS_2 −78% (10 N); graphite −64% (30 N)	PTFE film breakdown by heating; adhesive debris	[134]
PA12	MoS_2 1 wt%	SLS	n.d.	Recip. ball, 304 SS, 35 N, 0.33 m/s, dry	0.30 → 0.13	−41.7%	Tribochemical film (MoO_3, FeS by XPS)	[136]
PEEK	PTFE 2.5–10 wt%	FFF	Sliding ⊥ print texture	Recip. ball, GCr15, 30–100 N, 1 Hz, dry	−61% at 30 N (7.5 wt%); ≈0.1 at all loads	Minimum 2.72×10^{-5} $mm^3/(N\cdot m)$ at 7.5 wt%; 10 wt% worst	PTFE transfer film; micropores at 10 wt%	[199]
PEEK	hBN 5–15 wt%	LPBF	n.d.	Recip. ball, 304 SS, 50 N, 4 Hz, 20–200 °C	−65 to −73% (10 wt%)	−79 to −81% (10 wt%)	B/N-rich film; patchy at 15 wt%	[103]
PEEK	MoS_2 12 wt%, crystallinity varied	LPBF	Top surface; graded parts	Recip. ball, 304 SS, 50 N, 4 Hz	0.051 → 0.154 as Xc 35 → 45% (crystallinity effect)	6.28 → 0.53×10^{-5} $mm^3/(N\cdot m)$ (−92%, derived)	Rapid film at low Xc vs hardness at high Xc	[87]
PETG	hBN 1–5 wt%	FFF	Polished top face	Recip. ball, 100Cr6, 5–15 N, ≈0.1 m/s, dry	−33 to −46% (1 wt%, derived)	Track 4–5× shallower (1 wt%)	hBN tribofilm; agglomeration at 3–5 wt%	[210]
ABS	Graphite 2.5, 5 wt%	FFF	n.d.	Pin-on-disc, EN24, 5–20 N, 0.26–1.05 m/s, dry	0.65 → 0.22 (5 wt%)	Wear loss −27/−55% (10 N, 1 m/s)	Smeared graphite layer	[212]
ABS	Graphite 2.5–10 wt%	FFF	Raster angle and gap varied	Block-on-disc, grey cast iron, 35 N, 8.4 m/s, dry	Increased (max 0.43 at 2.5 wt%)	SWR increased with content	Flake–matrix cracking, flake pull-out	[67]
Urethane acrylate	PTFE, graphite, MoS_2 and blends	DLP	n.d.	Pin-on-disc, 100Cr6 Ra 0.3 μm, 25 N, 0.25 m/s, dry	−52% (PTFE/MoS_2)	−92% (PTFE/MoS_2)	n.r. (text truncated)	[91]
Acrylate	PFPE-loaded silica 5–30 wt%	DLP	Polished	Recip. ball, GCr15, 10 N, 3 Hz, dry	0.7 → 0.1 (20 wt%)	−97.7%	Oil + silica film (F, Si on ball)	[154]
PLA	Graphene 0.2–0.5 wt%	FFF	Sliding ⊥ raster	Recip., EN31 Ra 0.93 μm, 10 N, 2 Hz, dry	−76% (0.5 wt%)	Mass loss −58 to −64% (0.3 wt%)	Continuous carbon-rich transfer layer	[200]
PA12	GNP 2–10 wt%	SLS	XY face	Recip. ball, Al_2O_3, 2 N, ≤0.05 m/s, dry	0.52 → 0.38 (−27%, derived)	Volume −81% (10 wt%)	Self-lubrication; porosity rises above 6 wt%	[80]
PPS	CNT 0.1–0.9 wt%	FFF	n.d.	Block-on-ring, steel Ra 0.3–0.4 μm, 100/196 N, ≈0.42 m/s, dry	n.r. vs neat	−73% (100 N), −98% (196 N) (derived)	Crystallinity, conduction; severe-wear transition suppressed	[153]
PLA	GNP (commercial)	FFF	Plain and patterned skins	Ball-on-disc steel, 5 N, 16 mm/s; sled test	−68% (ball-on-disc); increase on some sled skins	n.r.	Test-dependent friction	[214]
PA12	Micro-SiC 10 wt%	SLS	n.d.	Ball-on-disc, 304 SS, 20 N, 360 rpm, dry	≈−10%	Mass −54%; SWR −20% (inconsistent)	Detached SiC third bodies, pits	[135]
PA12	μ-SiC, n-SiC, n-SiO_2	SLS	n.d.	Ball-on-disc, 304 SS, 20 N, ≈0.05 m/s (derived), seawater	μ-SiC lowest; n-SiO_2 ≈+30%	μ-SiC lowest; n-SiO_2 highest (graphical; conclusion inconsistent)	Abrasion, particle detachment	[79]
PA1212	Glass beads 40 + GF 0–20 wt%	SLS	n.d.	Ball-on-disc, GCr15, 10 N, ≈0.52 m/s, dry	−47% (GB + 10 GF, derived)	−93% (derived; absolute units uncertain)	Encapsulated fillers bear load; GF peeling at 20 wt%	[216]

Matrix	Filler (type, loading)	Process	Orientation / architecture	Test (config, counterface, load, speed, environment)	Effect on COF vs unfilled (within-study)	Effect on wear vs unfilled (within-study)	Dominant mechanism	Ref.
PEEK	hBN 3 wt%; β-Si_3N_4 10 wt%	FFF	n.d.	Flat pin-on-disc, CoCr Ra 0.2 μm, 96 N, 0.12 m/s, PBS	hBN ≈−20%; Si_3N_4 ≈+40%, fluctuating	Pin SWR ≈−50% (both); Si_3N_4 roughened CoCr (Sz 5–10 μm)	hBN film vs Si_3N_4 pull-out, third-body abrasion	[13]
ABS	Al_2O_3, hBN, Al_2O_3/hBN (sprayed per layer)	FFF + spray	Particle-rich interlayers	Recip. ball, SS, 10–100 N, 0.5 mm/s, dry	Al_2O_3 +249% (10 N), −10% (100 N)	n.r.	Load-dependent rolling vs ploughing; interlayer cohesion lost	[218]
PLA	Cu 20%, Al 20%	FFF	n.d.	Pin-on-disc, 316L ball, 15 N, 0.105 m/s, dry	No significant change	Cu −55%; Al not significant	Ploughing with lateral flow; pin scratched	[217]
PEEK	SCF 10 + nano-SiO_2 2 wt%	FFF	n.d.	Pin-on-plate, 316L Sa 0.25 μm, 80/160 N, 0.02 m/s, dry	Up to −50%	SWR up to −60%	SiO_2 suppresses debonding; denser film	[142]
PEEK	SCF 10 + Si_3N_4 2 + MoS_2 3 wt%	FFF	0° raster	Recip. ball, GCr15, 50–100 N, 0.3 m/s, dry	−20.5/−42.9/−50% (50/75/100 N)	Depth +21% (50, 75 N); −20.7% (100 N)	Load sharing; MoS_2 slip without continuous film	[198]
PEEK	SCF 5 + nano-SiO_2 2–6 wt% (alternating layers)	Dual-nozzle FFF	Alternating layers	Pin-on-disc, GCr15 Ra 0.05 μm, 2–25 MPa, 1 m/s, dry	0.07 → 0.05 (20 MPa, vs no SiO_2)	6.72 → 5.07×10^{-7} $mm^3/(N \cdot m)$ (−25%, derived; vs no SiO_2)	Third-body SiO_2 at 5 MPa; rolling particles and graphitised film at high pressure	[223]
PLA	SCF + GNP 0.5–5 wt% each	FFF	n.d.	Recip. ball, 100Cr6, 5 N, 8.5 Hz, dry	0.71 → 0.49 (−31%, derived; 0.5–1 wt%)	SWR −81% (5 wt% each, derived)	SCF load support; GNP film proposed	[231]
PLA	Biocarbon 5–30 vol%	FFF	n.d.	Recip. ball, Al_2O_3, 1 N, dry	Unchanged (0.4–0.6); less fluctuation	Volume decreased with content (graphical)	Fatigue + abrasion; no graphitic film	[224]
PLA	Corn cob 5–20 wt%	FFF	n.d.	Pin-on-disc, SS, 5–20 N, 0.4 m/s, dry	−9% (10 wt%)	Minimum SWR at 10 wt% (graphical)	Not characterised	[225]

Note: P/AP/N = fibres in the contact plane parallel/anti-parallel to sliding / fibres normal to the contact face; ∥/⊥ = parallel/perpendicular to sliding; n.d. = not determinable; n.r. = not reported; Xc = crystallinity; recip. = reciprocating; SS = stainless steel. "(derived)" marks ratios or percentages calculated here from values in the source. Wear is SWR unless stated (mass loss, depth or volume as reported). Velocities marked ≈ are converted from rpm or frequency in the source (derived). Implausible or irreconcilable values [16,228,229,296] are excluded.

Table S4. Study-level appraisal of reporting and evidential completeness of the 245 full-text experimental studies analysed (Section 2.4). Q1, complete test conditions (numeric load, speed or frequency, distance or duration, counterface material); Q2, surface characterised (numeric specimen or counterface roughness); Q3, replication with dispersion; Q4, outcome quantified in physical units (volume, specific wear rate or depth; numeric COF for friction-only studies); Q5, mechanism evidence beyond SEM (chemical or subsurface). Score = Q1 + … + Q5; class high (4–5), moderate (2–3), low (0–1); values flagged implausible in the extraction cap the class at low. Research groups link studies that share at least two authors.

Study ID	Ref.	Q1	Q2	Q3	Q4	Q5	Score	Implausible values	Class	Group
Akdogan2026	[186]	✓	–	✓	✓	–	3		moderate	G082
Akhan2025	[332]	–	–	–	✓	–	1		low	G049
Alabir2023	[231]	✓	–	✓	✓	–	3		moderate	G120
Albahkali2023	[286]	–	✓	✓	✓	–	3		moderate	G121
Alsaadi2023	[180]	–	–	✓	✓	–	2	yes	low	G067
Aly2026	[190]	–	✓	✓	–	–	2		moderate	G083
Amiruddin2019	[295]	✓	✓	✓	✓	–	4	yes	low	G011
Andabloreyes2020	[282]	–	✓	–	✓	–	2		moderate	G004
Ando2022	[123]	✓	✓	–	–	–	2		moderate	G022
Arreaza2026	[178]	✓	–	✓	✓	–	3		moderate	G090
Arslanacicbe2026	[183]	–	–	✓	✓	–	2		moderate	G072
Asker2024	[287]	✓	✓	–	✓	–	3		moderate	G121
Avalappa2025	[242]	✓	✓	–	–	–	2		moderate	G145
Avilalopez2022	[158]	–	–	–	✓	✓	2		moderate	G118
Azim2026	[75]	✓	✓	✓	✓	–	4		high	G170
Aziz2020	[272]	✓	–	–	✓	–	2		moderate	G017
Bacescu2026	[42]	✓	–	✓	–	–	2		moderate	G160
Badicioiu2026	[293]	✓	–	–	✓	✓	3		moderate	G026
Bai2019	[136]	✓	–	✓	✓	–	3		moderate	G107
Balaji2025	[230]	–	–	–	✓	–	1	yes	low	G148
Balaji2025a	[226]	✓	–	–	✓	–	2		moderate	G148
Bartolomeu2019	[12]	✓	✓	✓	–	✓	4		high	G010
Batakliev2026	[333]	–	–	–	–	–	0		low	G169
Batista2023	[205]	–	–	–	–	–	0		low	G023
Batista2024	[290]	–	✓	–	✓	–	2		moderate	G023
Batista2024a	[217]	✓	–	–	✓	–	2		moderate	G023

Study ID	Ref.	Q1	Q2	Q3	Q4	Q5	Score	Implausible values	Class	Group
Batista2025	[132]	✓	✓	–	✓	–	3		moderate	G023
Binabdollah2020	[128]	✓	–	–	✓	–	2		moderate	G011
Boparai2015	[220]	✓	–	–	–	–	1		low	G101
Boparai2016	[221]	✓	–	–	✓	–	2		moderate	G101
Bora2025	[185]	✓	–	✓	✓	–	3		moderate	G156
Braileanu2023	[318]	✓	–	–	✓	–	2		moderate	G044
Braileanu2025	[206]	✓	–	✓	✓	–	3		moderate	G044
Braileanu2025a	[107]	✓	✓	✓	–	–	3	yes	low	G044
Braileanu2026	[244]	✓	✓	✓	✓	–	4		high	G044
Briones2025	[133]	✓	–	✓	✓	–	3		moderate	G043
Cheng2026a	[76]	✓	–	–	✓	–	2		moderate	G174
Chhabra2023	[264]	✓	–	–	✓	–	2		moderate	G124
Chisiu2021	[130]	–	✓	–	✓	–	2		moderate	G175
Choi2026	[276]	✓	✓	–	–	✓	3		moderate	G006
Cicek2025	[175]	✓	–	✓	✓	–	3		moderate	G076
Cingoz2025	[137]	✓	–	–	✓	–	2		moderate	G157
Ciobanu2025	[252]	–	–	–	✓	–	1		low	G042
Cui2025	[99]	✓	✓	✓	✓	–	4		high	G150
Cui2026	[291]	✓	✓	✓	✓	✓	5		high	G150
Dangnan2020	[35]	✓	✓	–	✓	–	3		moderate	G062
Dawoud2015	[67]	✓	✓	–	✓	–	3		moderate	G102
Demirci2025	[327]	–	✓	–	–	–	1		low	G153
Deng2026	[41]	✓	✓	–	✓	✓	4		high	G051
Deng2026a	[15]	✓	✓	–	✓	✓	4		high	G051
Dhakal2023	[64]	✓	✓	✓	✓	✓	5		high	G024
Dhakal2024	[53]	✓	✓	✓	✓	✓	5		high	G024
Dhakal2026	[142]	✓	✓	✓	✓	✓	5		high	G024
Dhandapani2023	[303]	–	–	–	✓	–	1		low	G122
Dikenturksayar2024	[184]	–	–	✓	✓	–	2		moderate	G072

Study ID	Ref.	Q1	Q2	Q3	Q4	Q5	Score	Implausible values	Class	Group
Dobos2024	[253]	–	–	–	–	–	0		low	G033
Dontsov2020	[314]	✓	✓	✓	✓	–	4		high	G110
Doumit2025	[19]	–	–	–	✓	–	1		low	G071
Du2024	[154]	✓	✓	–	✓	✓	4		high	G138
Equbal2010	[263]	✓	✓	–	✓	–	3		moderate	G008
Ergene2025	[247]	–	✓	–	✓	–	2		moderate	G151
Ergene2026	[246]	✓	✓	✓	✓	–	4		high	G056
Ertane2018	[224]	–	–	–	✓	–	1		low	G105
Farimani2020	[267]	–	–	–	✓	–	1		low	G018
Fidan2025	[122]	–	–	✓	✓	–	2		moderate	G016
Fidan2026	[57]	✓	–	✓	✓	–	3		moderate	G016
Fidan2026a	[58]	✓	–	✓	✓	–	3		moderate	G016
Fink2026	[102]	–	✓	✓	✓	–	3		moderate	G162
Firlej2021	[308]	–	✓	✓	✓	–	3		moderate	G077
Firlej2025	[170]	✓	–	–	✓	–	2		moderate	G077
Fouly2022	[225]	✓	✓	✓	✓	–	4		high	G117
Gad2023	[176]	–	–	✓	✓	–	2		moderate	G069
Gadelmoula2023	[33]	✓	✓	–	✓	–	3		moderate	G119
Gadelmoula2023a	[235]	✓	✓	–	✓	–	3		moderate	G119
Gadelmoula2023b	[202]	✓	✓	–	✓	–	3		moderate	G119
Garg2015	[219]	✓	–	–	–	–	1		low	G103
Gentle2026	[169]	✓	–	✓	✓	–	3		moderate	G088
Gkougkousi2024	[279]	–	✓	✓	✓	–	3		moderate	G135
Goia2026	[306]	✓	✓	✓	✓	✓	5		high	G164
Goncalves2007	[161]	–	✓	–	–	–	1		low	G059
Grymak2022	[181]	✓	–	✓	✓	–	3		moderate	G066
Grymak2023	[196]	✓	–	✓	–	–	2	yes	low	G066
Grymak2025	[182]	✓	✓	✓	✓	–	4		high	G066
Gundogan2026	[194]	–	–	✓	✓	–	2		moderate	G089

Study ID	Ref.	Q1	Q2	Q3	Q4	Q5	Score	Implausible values	Class	Group
Guo2026	[199]	✓	✓	–	✓	✓	4		high	G031
Guven2023	[177]	–	–	✓	✓	–	2		moderate	G125
Hanon2020	[236]	–	✓	–	✓	–	2		moderate	G019
Hanon2020a	[126]	–	✓	–	✓	–	2		moderate	G019
Hanon2021	[66]	✓	✓	–	✓	–	3		moderate	G019
Hanon2022	[89]	✓	✓	✓	✓	–	4		high	G019
Harishbabu2026	[228]	–	–	–	✓	–	1	yes	low	G163
He2023a	[140]	✓	✓	–	✓	–	3		moderate	G030
He2023b	[54]	✓	–	–	✓	–	2		moderate	G027
Hong2017	[275]	✓	✓	–	✓	–	3		moderate	G009
Hossain2023	[146]	✓	–	–	✓	–	2		moderate	G031
Houshmand2025	[164]	✓	–	–	✓	–	2		moderate	G155
Hu2019	[274]	–	–	–	✓	–	1		low	G003
Hu2024a	[159]	✓	–	–	–	–	1		low	G137
Islam2026	[17]	✓	✓	✓	✓	–	4		high	G168
Jiang2020	[162]	–	–	–	✓	–	1		low	G061
Kanafi2017	[106]	–	–	–	–	–	0		low	G001
Karunanithi2024	[258]	✓	–	✓	✓	–	3		moderate	G035
Kashyap2025	[160]	✓	–	✓	✓	–	3		moderate	G078
Katsivardi2025	[214]	✓	–	✓	✓	✓	4		high	G135
Keshavamurthy2021	[212]	–	✓	–	✓	–	2		moderate	G113
Kessler2019	[168]	–	–	✓	✓	–	2		moderate	G108
Kessler2026	[173]	✓	–	✓	✓	–	3		moderate	G087
Khan2024	[278]	–	–	–	–	✓	1		low	G037
Kokol2026	[191]	–	✓	✓	–	–	2		moderate	G091
Kozior2024	[82]	✓	✓	–	✓	–	3		moderate	G097
Krupa2026	[65]	✓	✓	✓	✓	–	4		high	G054
Kujawa2025	[50]	✓	–	–	✓	–	2	yes	low	G045
Kwon2025	[88]	✓	✓	–	✓	–	3		moderate	G081
Lasch2026	[16]	✓	✓	✓	✓	–	4	yes	low	G022

Study ID	Ref.	Q1	Q2	Q3	Q4	Q5	Score	Implausible values	Class	Group
Laurino2025	[93]	✓	–	✓	✓	✓	4		high	G158
Lawson2025	[11]	✓	–	✓	✓	–	3		moderate	G079
Lee2022	[9]	–	–	✓	✓	–	2		moderate	G065
Lei2024	[187]	✓	–	–	✓	–	2		moderate	G070
Lei2026	[239]	✓	–	–	–	–	1		low	G027
Li2024a	[155]	–	✓	–	✓	✓	3		moderate	G142
Li2024b	[203]	✓	✓	✓	✓	–	4		high	G143
Li2025a	[101]	–	✓	–	✓	–	2		moderate	G046
Li2026	[103]	✓	–	–	✓	✓	3		moderate	G031
Li2026a	[40]	✓	–	–	✓	✓	3		moderate	G031
Li2026b	[149]	–	–	✓	–	–	1	yes	low	G058
Li2026c	[150]	✓	✓	–	✓	–	3		moderate	G007
Li2027	[87]	✓	✓	–	✓	✓	4		high	G031
Lin2025	[138]	✓	–	✓	✓	✓	4		high	G031
Liu2025	[97]	✓	–	–	✓	✓	3		moderate	G154
Luo2022	[59]	✓	✓	–	✓	✓	4		high	G051
Luo2026	[6]	✓	✓	–	✓	–	3		moderate	G166
Lv2024	[223]	–	✓	✓	✓	✓	4		high	G030
Ma2022	[79]	✓	✓	–	✓	✓	4		high	G115
Ma2022a	[96]	✓	–	✓	✓	–	3		moderate	G005
Mahad2024	[256]	–	–	–	–	–	0		low	G037
Mahesh2024	[328]	✓	–	–	✓	–	2		moderate	G136
Mahmood2023	[125]	✓	–	–	✓	–	2		moderate	G025
Mahmood2025	[116]	✓	✓	✓	✓	✓	5		high	G025
Mahran2025	[195]	–	–	✓	✓	–	2	yes	low	G146
Man2021	[73]	✓	✓	✓	✓	–	4		high	G051
Man2022	[233]	–	✓	–	✓	–	2		moderate	G051
Markovic2026	[61]	✓	✓	✓	–	–	3		moderate	G050
Massocchi2021	[316]	✓	–	✓	✓	–	3		moderate	G034
Massocchi2024	[8]	✓	✓	–	✓	–	3		moderate	G034

Study ID	Ref.	Q1	Q2	Q3	Q4	Q5	Score	Implausible values	Class	Group
Maydanshahi2024	[49]	✓	✓	–	✓	–	3		moderate	G036
Minetola2014	[324]	✓	–	✓	–	–	2		moderate	G100
Mingione2025	[80]	✓	✓	✓	✓	✓	5		high	G159
Mital2022	[144]	✓	–	–	–	–	1		low	G021
Mital2023	[145]	✓	–	–	–	–	1		low	G021
Mosco2025	[60]	–	✓	✓	✓	–	3		moderate	G098
Mourya2023	[124]	–	–	–	✓	–	1		low	G029
Muminovic2024	[152]	–	–	–	–	–	0		low	G038
Murwamadala2026	[257]	✓	✓	✓	✓	–	4		high	G165
Myagmar2021	[179]	–	✓	✓	✓	–	3		moderate	G064
Mzali2024	[115]	✓	✓	–	✓	–	3		moderate	G039
Naik2022	[74]	✓	–	–	✓	–	2		moderate	G116
Nar2023	[134]	✓	✓	✓	✓	–	4		high	G126
Nedelcu2020	[334]	✓	✓	✓	✓	–	4	yes	low	G013
Norani2021	[241]	✓	✓	–	✓	–	3		moderate	G011
Olesik2019	[151]	✓	–	–	✓	✓	3		moderate	G106
Ozbaykisasoz2023	[259]	✓	✓	–	✓	–	3		moderate	G127
Paduraru2026	[68]	✓	✓	–	✓	–	3		moderate	G052
Palma2019	[36]	✓	✓	–	–	–	2		moderate	G096
Pan2021	[153]	✓	✓	–	✓	–	3		moderate	G114
Pant2020	[243]	✓	✓	–	✓	–	3		moderate	G015
Park2018	[174]	✓	–	✓	✓	–	3		moderate	G060
Pisula2021	[5]	–	–	–	–	–	0	yes	low	G020
Poporogu2024	[251]	✓	–	–	✓	–	2		moderate	G176
Porojan2026	[171]	✓	✓	✓	✓	–	4	yes	low	G084
Portoaca2023	[245]	✓	–	–	✓	–	2		moderate	G026
Portoaca2024	[261]	–	–	–	✓	–	1		low	G026
Prabakaran2026	[127]	–	✓	✓	✓	✓	4		high	G055
Prajapati2024	[141]	✓	✓	–	✓	✓	4		high	G040
Prajapati2024a	[34]	✓	✓	–	✓	✓	4		high	G040

Study ID	Ref.	Q1	Q2	Q3	Q4	Q5	Score	Implausible values	Class	Group
Prusinowski2020	[208]	–	–	–	✓	–	1		low	G111
Przekop2020	[211]	✓	✓	✓	✓	–	4		high	G109
Rachaiah2024	[335]	✓	–	–	✓	–	2		moderate	G130
Rajendran2024	[84]	✓	✓	–	✓	–	3		moderate	G131
Ralls2024	[218]	✓	–	✓	–	✓	3		moderate	G132
Ravi2026	[298]	✓	✓	✓	✓	–	4	yes	low	G167
Ripeanu2024	[260]	✓	–	–	✓	–	2		moderate	G026
Santo2024	[200]	✓	✓	✓	–	✓	4		high	G139
Shrestha2026	[13]	✓	✓	✓	✓	✓	5		high	G024
Siddikali2025	[336]	–	–	–	✓	–	1		low	G149
Simeon2024	[193]	✓	–	✓	✓	–	3		moderate	G071
Simonovic2026	[81]	✓	✓	✓	✓	–	4		high	G161
Singh2016	[299]	✓	–	–	–	–	1	yes	low	G104
Sinmazcelik2020	[277]	–	✓	–	✓	–	2		moderate	G016
Sirin2024	[254]	✓	–	–	✓	–	2	yes	low	G144
Sivakumar2024	[227]	✓	✓	✓	✓	–	4		high	G141
Slapnik2020	[91]	✓	✓	–	✓	–	3		moderate	G112
SnarskiAdamski2025	[104]	–	✓	✓	✓	✓	4		high	G074
Soni2026a	[229]	✓	–	–	✓	–	2	yes	low	G172
Soni2026b	[315]	–	✓	–	✓	–	2		moderate	G172
Sood2012	[262]	–	✓	–	✓	–	2	yes	low	G008
Stoica2025	[147]	✓	–	✓	✓	–	3		moderate	G048
Stoica2026	[148]	–	–	–	–	–	0		low	G048
Struz2024	[129]	–	✓	–	–	–	1		low	G032
Tanisan2026	[325]	–	–	✓	✓	✓	3		moderate	G057
Tanthasri2026	[165]	✓	✓	✓	✓	–	4	yes	low	G080
Tian2025	[32]	✓	–	–	✓	✓	3		moderate	G027
Tuerksayar2024	[166]	✓	–	✓	✓	–	3		moderate	G073
Tyagi2024	[296]	✓	✓	–	✓	–	3	yes	low	G140
Tyagi2025	[297]	–	✓	–	✓	–	2	yes	low	G140
Vasiliu2026	[189]	✓	✓	✓	✓	–	4	yes	low	G084

Study ID	Ref.	Q1	Q2	Q3	Q4	Q5	Score	Implausible values	Class	Group
Vaught2023	[51]	–	–	✓	✓	✓	3		moderate	G028
Vazquezmartinez2022	[131]	✓	–	–	✓	–	2		moderate	G023
Veerapeindee2025	[188]	✓	–	✓	✓	–	3		moderate	G080
Vesco2025	[108]	✓	✓	–	✓	–	3		moderate	G047
Vilhena2019	[281]	–	✓	✓	✓	–	3		moderate	G012
Wada2023	[92]	✓	–	✓	✓	–	3		moderate	G068
Wan2024	[167]	✓	–	✓	✓	–	3		moderate	G064
Wang2024	[39]	✓	–	–	✓	–	2		moderate	G031
Wang2024b	[248]	–	✓	–	✓	✓	3		moderate	G133
Wang2026	[139]	✓	✓	✓	✓	✓	5		high	G031
Wesemann2021	[10]	✓	–	✓	✓	–	3		moderate	G063
Woerz2018	[85]	–	✓	–	✓	✓	3		moderate	G095
Wu2023	[216]	✓	–	–	✓	–	2		moderate	G123
Wu2023a	[337]	✓	–	✓	✓	–	3		moderate	G128
Wu2023b	[157]	✓	–	–	✓	–	2		moderate	G031
Wu2023c	[338]	–	–	–	✓	–	1		low	G129
Wu2026	[172]	✓	–	–	✓	–	2		moderate	G086
Xu2025	[62]	✓	✓	✓	✓	–	4		high	G152
Xu2025a	[163]	–	✓	–	–	–	1		low	G075
Xu2025b	[301]	✓	✓	✓	✓	✓	5		high	G152
Xu2026	[232]	✓	✓	✓	✓	✓	5		high	G152
Yan2026	[300]	–	–	–	✓	✓	2		moderate	G030
Yang2026	[198]	✓	–	–	✓	✓	3		moderate	G173
Yao2024	[289]	–	✓	–	✓	✓	3		moderate	G031
Yasan2026	[210]	✓	–	✓	✓	✓	4		high	G171
Yilmaz2024a	[121]	✓	–	✓	✓	✓	4		high	G041
Yilmaz2024b	[204]	✓	–	–	✓	–	2		moderate	G134
Yosef2026	[120]	–	–	–	–	–	0		low	G085
Yu2022	[135]	–	✓	–	✓	✓	3	yes	low	G115

Study ID	Ref.	Q1	Q2	Q3	Q4	Q5	Score	Implausible values	Class	Group
Yuan2019	[95]	–	–	–	✓	–	1		low	G002
Yujie2026	[56]	✓	–	–	✓	–	2		moderate	G092
Zdravkovic2026	[249]	✓	–	✓	✓	–	3	yes	low	G053
Zhang2020	[4]	✓	–	–	✓	–	2		moderate	G014
Zhang2020a	[339]	✓	✓	–	✓	–	3	yes	low	G009
Zhang2023	[98]	–	–	–	✓	–	1		low	G030
Zhang2025a	[55]	✓	–	–	✓	–	2		moderate	G147
Zhang2025b	[238]	✓	–	–	✓	–	2		moderate	G027
Zhang2026b	[94]	✓	–	–	✓	✓	3		moderate	G093
Ziegler2018	[86]	✓	–	–	✓	–	2		moderate	G094
Zielinski2025	[271]	–	✓	✓	✓	–	3		moderate	G099

Note: ✓, criterion met; –, not met. Criteria were applied to the full-text extraction with conservative text rules (analysis scripts available from the authors on request).

Table S5. Coverage and representativeness of the evidence base (Section 2.2). (a) Web of Science eligible polymer records by route: records included in the synthesis versus eligible records not selected; route assigned from title, abstract and keywords by keyword rules (unspecified: no route named). (b) Publication period. (c) Citations per year. (d) All 289 included experimental studies by route (from the process field and title of each record).

**Item**	**Included (WoS)**	**Eligible, not included (WoS)**	**Test**
(a) Material extrusion (FFF/FDM)	135 (51%)	96 (59%)	χ^2 = 10.1, permutation p = 0.05 (20 000 permutations)
(a) Powder bed fusion (SLS/MJF)	26 (10%)	6 (4%)	
(a) Vat photopolymerisation (SLA/DLP)	22 (8%)	8 (5%)	
(a) Material jetting (PolyJet)	1 (<1%)	0 (0%)	
(a) Direct ink writing	2 (1%)	0 (0%)	
(a) Unspecified 3D printing	78 (30%)	53 (33%)	
(a) Total	264	163	
(b) ≤2018	14 (5%)	9 (6%)	$\chi^2(4)$ = 2.0, p = 0.73
(b) 2019–2021	42 (16%)	26 (16%)	
(b) 2022–2023	52 (20%)	26 (16%)	
(b) 2024–2025	100 (38%)	59 (36%)	
(b) 2026	56 (21%)	43 (26%)	
(c) Citations per year, median	3.4	1.0	Mann–Whitney p < 0.001
(d) All 289 experimental studies: material extrusion 174 (60%), vat photopolymerisation 56 (19%), powder bed fusion 31 (11%), material jetting 5 (2%), direct ink writing 3 (1%), route not stated 20 (7%)			

Note: Keyword rules and record-level assignments are reproducible from the screening log of the evidence workbook. Of the 1605 eligible Web of Science records, 427 were classed as polymer records; metal, metal-matrix and ceramic records were assigned to the companion review. The 264 included Web of Science records comprise the 72 core and 95 open-access-extension studies, the 89 additions from the eligible pool and 8 eligible-pool records included through the OpenAlex re-check (Section 2.2). The route test uses a permutation of inclusion labels because two route categories contain fewer than five records.

Table S6. Established findings: scope and sensitivity to the grading rule (Sections 2.5 and 18.1). Baseline rule: consistent direction in ≥3 studies from ≥2 research groups, ≥2 with moderate or high appraisal, no opposing moderate/high-appraisal study. Variants: (A) ≥3 moderate/high studies from ≥3 groups; (B) at least one high-appraisal study; (C) appraisal on the first four criteria only; (D) research groups merged at one shared author (121 groups); (E) all variants combined. ✓ retained as established; – would become probable.

Finding	Scope	M/H studies / groups	A	B	C	D	E	Moderate/high-appraisal supporting studies
Orientation is associated with a switch between interlayer-shear- and ridge-crossing-dominated wear	Broad (MEX, PBF; several polymers)	6 / 5	✓	✓	✓	✓	✓	[32,33,36,62,116,125]
End-on fibres give the highest wear in dry FFF composites under moderate–severe contact	Specific (dry FFF composites)	4 / 3	✓	✓	✓	✓	–	[62,73,74,232]
Topographic control of friction is lost as load rises	Broad (MEX, MJT)	5 / 5	✓	✓	✓	✓	✓	[35,64,66,115,116]
Friction on as-printed FFF surfaces is directional	Specific (FFF)	3 / 3	✓	✓	✓	✓	✓	[115,116,130]
Running-in smooths printed texture	Specific (FFF, several polymers)	4 / 4	✓	✓	✓	✓	✓	[61,64–66]
Hardness alone is not a transferable wear predictor	Broad (3 classes; sliding and chewing)	7 / 7	✓	✓	✓	✓	✓	[10,11,65,104,128,204,308]
Oil and grease lower the friction of printed polymers	Specific (extruded thermoplastics)	5 / 5	✓	–	✓	✓	–	[8,54,55,61,272]
Short carbon fibre lowers COF in engineering matrices (wear effect conflicting)	Specific (engineering matrices)	6 / 6	✓	✓	✓	✓	✓	[6,53,59,99,202,203]
PTFE and lamellar solids lower COF through transfer films	Broad (PBF, MEX, VPP)	6 / 5	✓	✓	✓	✓	✓	[91,103,134,136,199,210]
Thicker layers raise COF when sliding crosses the rasters (MEX)	Specific (MEX)	4 / 4	✓	–	✓	✓	–	[39,108,131,238]
Higher infill lowers wear when the infill is exposed or immersed	Specific (MEX)	4 / 4	✓	✓	✓	✓	✓	[34,246–248]
Post-cure lowers the wear of photopolymers	Specific (dental VPP)	3 / 3	✓	–	✓	✓	–	[92,167,172]
In dental resins, formulation dominates process settings	Specific (dental VPP)	6 / 6	✓	✓	✓	✓	✓	[93,166–169,193]
Dental printed-versus-conventional rankings depend on resin class, wear mode and duration	Specific (dental VPP)	5 / 5	✓	✓	✓	✓	✓	[10,11,173,179,182]
Unfilled resins sliding dry on steel reach steady COF 0.43–0.76	Specific (VPP/MJT resins on steel)	6 / 6	✓	✓	✓	✓	–	[35,88,89,94,154,155]

Finding	Scope	M/H studies / groups	A	B	C	D	E	Moderate/high-appraisal supporting studies
Annealing lowers the dry wear of FFF PEEK (conditional on load and filament direction)	Specific (FFF PEEK, dry)	4 / 3	✓	✓	✓	–	–	[41,138–140]
Transfer films control mild wear of printed thermoplastics	Broad (several polymers and routes)	7 / 6	✓	✓	✓	✓	✓	[40,41,51,134,136,138,140]
Oxidative (tribochemical) wear occurs in printed polymers	Broad (PEEK, PA12)	5 / 3	✓	✓	✓	–	–	[53,136,142,223,300]
Interlayer weld failure is the wear mechanism most specific to material extrusion	Specific (MEX, by definition)	7 / 7	✓	✓	✓	✓	✓	[4,32,99,116,122,127,128]
Groove-trapped debris acts as a third body (MEX)	Specific (MEX)	4 / 4	✓	✓	✓	✓	✓	[131,139,141,257]
Under cyclic contact, damage moves below the surface (subsurface fatigue)	Broad (dental resins, gears)	6 / 6	✓	✓	✓	✓	✓	[4,93,101,168,173,185]
Process → architecture link (MEX and PBF)	Route-specific (MEX, PBF)	10 / 7	✓	✓	✓	✓	✓	[4,34,39,40,60,87,127,137,138,216]
Architecture → surface link (MEX)	Route-specific (MEX)	6 / 6	✓	✓	✓	✓	✓	[39,64,116,127,131,141]
Dense, machined printed polymer is not intrinsically inferior to its conventional counterpart	Specific (three systems)	3 / 3	✓	✓	✓	✓	✓	[51,97,314]

Note: Of the 81 audited claims, 80 carry a single (first-listed) grade: 24 established, 27 probable, 17 conflicting and 12 insufficient; the remaining entry covers the cells of the mechanism map (Figure 8). The baseline rule, applied mechanically to the supporting and opposing studies of each claim, reproduced all 24 established grades. Relaxing the rule to two moderate/high studies from two groups would upgrade 14 of the 27 probable claims. Broad: supported across at least two routes or material classes; specific: confined to one route, polymer family or condition. For split claims only the established part and its supporting studies are listed.

Table S7. Robustness of within-study ratio summaries (Section 16.3) to non-independence of ratios, study appraisal and selection. Factors and metrics with at least five ratios. Medians of treated/reference ratios (orientation: highest/lowest); one-per-study and one-per-group summaries use the median ratio within each study and the median of study values within each group; 95% interval of the median from 4000 bootstrap resamples of research groups; leave-one-group-out range of the median (all derived).

Factor	Metric	Ratios / studies / groups	Median (all ratios; Table 7)	One per study	One per group	High/moderate only	Bootstrap 95% interval	Leave-one-group-out	Studies with median ratio <1
Filler	COF	35 / 27 / 22	0.77	0.73	0.73	0.74 (n=30)	0.63–0.92	0.75–0.79	25/27
Filler	wear	22 / 16 / 13	0.52	0.52	0.39	0.54 (n=19)	0.27–0.72	0.39–0.54	15/16
Orientation	wear	24 / 19 / 16	1.54	1.66	1.54	1.66 (n=21)	1.29–3.19	1.42–1.68	n/a (≥1 by construction)
Orientation	COF	13 / 11 / 9	1.22	1.22	1.20	1.21 (n=12)	1.18–1.28	1.20–1.22	n/a (≥1 by construction)
Annealing (thermoplastics)	wear	9 / 5 / 5	0.44	0.44	0.44	0.44 (n=9)	0.24–0.80	0.41–0.62	5/5
Lubrication	COF	6 / 3 / 3	0.61	0.71	0.71	0.61 (n=6)	0.50–1.36	0.50–0.71	3/3
Environment (ageing, temperature, humidity)	COF	7 / 5 / 4	1.20	1.22	1.26	1.20 (n=7)	1.15–1.98	1.17–1.30	0/5
Environment (ageing, temperature, humidity)	wear	15 / 5 / 5	1.26	1.21	1.21	1.26 (n=15)	1.18–1.70	1.21–1.27	0/5
Layer height	wear	5 / 5 / 5	0.95	0.95	0.95	0.95 (n=5)	0.39–1.51	0.79–1.23	3/5
Infill density	wear	6 / 4 / 4	1.02	1.02	1.02	1.09 (n=3)	0.58–1.80	0.94–1.09	2/4
Infill density	COF	8 / 5 / 5	1.00	0.92	0.92	0.96 (n=6)	0.85–1.27	0.89–1.10	3/5

Note: Effect magnitude (median $|\log_{10}$ ratio$|$ per study, orientation excluded) was unrelated to citations per year (Spearman $\varrho = 0.05$, $p = 0.74$; 54 studies); median $|\log_{10}$ ratio$|$ was 0.29 (ratio level) and 0.31 (study level) for wear and 0.11 and 0.15 for COF.

Table S8. Specific wear rates of printed neat PEEK (Dataset S2) by contact class, environment and specimen condition (Section 16.2).

SWR, $mm^3/(N \cdot m)$	Contact class (configuration, severity)	Environment	Specimen condition / note	Test configuration class	Ref.
6.20×10^{-7}	Conformal (flat pin on disc)	Dry	re-entrant, annealed	Unidirectional pin/ball-on-disc	[41]
1.32×10^{-6}	Conformal (flat-on-flat, 5–10 MPa)	Dry	10 MPa	Linear reciprocating	[142]
1.37×10^{-6}	Conformal (block-on-ring, 1 MPa)	Dry	minimum (200 C anneal)	Ring-type (block/plate/pin-on-ring)	[140]
1.74×10^{-6}	Conformal (flat pin on disc)	Dry	solid, annealed	Unidirectional pin/ball-on-disc	[41]
3.18×10^{-6}	Conformal (flat pin on disc)	Dry	re-entrant	Unidirectional pin/ball-on-disc	[41]
3.90×10^{-6}	Conformal (flat-on-flat, 5–10 MPa)	Dry	5 MPa	Linear reciprocating	[142]
6.20×10^{-6}	Conformal (flat pin on disc)	Dry	solid	Unidirectional pin/ball-on-disc	[41]
1.09×10^{-5}	Conformal (block-on-ring)	Dry	unmodified	Ring-type (block/plate/pin-on-ring)	[248]
1.45×10^{-5}	Conformal (pin-on-ring, 4 MPa)	Dry	4 MPa, 1 m/s	Ring-type (block/plate/pin-on-ring)	[300]
1.66×10^{-5}	Concentrated (steel ball, 87–148 MPa mean Hertzian)	Serum	serum; derived (unit-corrected), 14 N	Unidirectional pin/ball-on-disc	[49]
2.83×10^{-5}	Concentrated (steel ball, 30 N)	Dry	190 C anneal, 30 N	Linear reciprocating	[138]
3.70×10^{-5}	Concentrated (steel ball, 5 N)	Dry	median	Linear reciprocating	[6]
4.40×10^{-5}	Conformal (flat pin-on-disc, ≈6 MPa)	PBS	PBS-lubricated	Unidirectional pin/ball-on-disc	[13]
4.80×10^{-5}	Concentrated (steel ball, 217–264 MPa Hertzian)	Dry	90 N, annealed (approx.)	Linear reciprocating	[139]
7.31×10^{-5}	Concentrated (steel ball, 217–264 MPa Hertzian)	Dry	70 N, lowest of array	Linear reciprocating	[139]
7.40×10^{-5}	Conformal (pin-on-disc, 20 N)	Dry	80% infill	Unidirectional pin/ball-on-disc	[34]
1.01×10^{-4}	Concentrated (steel ball, 87–148 MPa mean Hertzian)	Serum	serum; derived, 30 N	Unidirectional pin/ball-on-disc	[49]
1.04×10^{-4}	Concentrated (steel ball, 217–264 MPa Hertzian)	Dry	70 N, highest of array	Linear reciprocating	[139]
1.18×10^{-4}	Concentrated (steel ball, 217–264 MPa Hertzian)	Dry	90 N	Linear reciprocating	[139]
1.28×10^{-4}	Conformal (pin-on-disc, 20 N)	Dry	40% infill	Unidirectional pin/ball-on-disc	[34]
2.45×10^{-4}	Concentrated (steel ball, 87–148 MPa mean Hertzian)	Serum	serum; derived, 70 N	Unidirectional pin/ball-on-disc	[49]
2.65×10^{-4}	Concentrated (steel ball, 87–148 MPa mean Hertzian)	Serum	serum; derived, 50 N	Unidirectional pin/ball-on-disc	[49]

Note: Dry, solid (non-lattice) specimens: conformal contacts, median 3.9×10^{-6} (7 values, 5 studies; range 1.4 decades); concentrated steel-ball contacts, median 6.1×10^{-5} (6 values, 3 studies), 1.2 decades higher. Re-entrant lattice specimens [41] and exposed-infill specimens [34] are listed but excluded from these medians. Within-study ranges: annealing 0.55–0.71 decades; architecture 0.29; infill 40% vs 80% 0.24; parameter array 0.15; load 14→50 N in serum 1.2; pressure 5→10 MPa −0.47 decades (all derived).

Table S9. Extended version of Table 3: reported effects of AM process parameters on friction and wear, with material classes, magnitudes and all supporting studies (within-study comparisons; arrows give the direction for an increase in the parameter or the stated change; consistency column uses the evidence grades of Section 2.5).

Parameter	Material classes studied	Typical direction of effect on COF	Typical direction of effect on wear	Magnitude range (within-study)	Consistency	Main pathway	Key refs
Contact face/layer direction vs sliding (MEX, neat)	PLA, ABS, PETG, HT-PLA, PEI, PA, PEEK	Edge-on across: highest; edge-on parallel: lowest; steady state often insensitive	PLA/ABS dry, printed body wearing: edge-on parallel worst, across best; PEEK: filaments parallel best; reverses when the printed body conditions the counterface	Wear 1.15–6.2× (load-dependent) [116,125]; 4.4× (cylinder) [126]; abrasion 1.27× Z/X, 2.1× between rasters [144]; ABS 1.27× [125]	Mechanism switch established; sign established for PLA/ABS dry, conflicting across polymers [35,140]; "vertical vs horizontal" conflicting until coded; explanation probable	Interlayer weld shear vs interlocking; groove debris channelling; topography (line contact)	[32,35,66,116,125,126,133,140,143–145,236,237]
Build orientation (PBF)	PA12, GB/CF/graphite-PA12, PA-GF	Mostly insensitive at steady state; up to 60% lower when sliding parallel to layers	Filler-dependent: face-on worst (GB-, CF-PA12) or best (graphite/CF-PA12)	1.48× [33]; 0.75–2.42% of COF variance [84]	Conflicting (sign and existence)	Interlayer/particle-boundary cohesion; filler exposure; counterface roughness	[33,36,84–86,202,235,327]
Fibre direction vs sliding	Short/continuous CF-PEEK, CF/PA6, CF-PA	End-on (N) raises COF in FFF (2×)	N worst in FFF and continuous fibre; N best in PBF; in-plane transverse best in water	4.1× [62]; 2.7–7.8× [73]; 1.75× height loss, abrasion [74]; 0.72× [6]; 0.60–0.65× [291]	Established for FFF (dry, moderate–severe contact); reversal at low severity (PBF) probable; fibre-over-layer ranking probable (one study)	Fibre load support vs fibre-end fracture and third bodies	[6,62,73,74,99,232,233,291,340,341]
Layer height (MEX)	PLA, ABS, ASA, PA, PEEK, PETG, PLA/graphene	↑ when sliding crosses rasters; ↓ when paths align with the track	↑ in short/abrasive tests; ↓ in some deep-wear tests	COF +40–70% [131,238,239]; wear +51% to >2× [122,241]; −38% [242]	COF established; wear conflicting	Topography (Sz 5.4× [141]); interlayer bonding	[38,39,122,131,132,141,238–243,257]
Layer thickness (VPP)	Dental methacrylates	n.r.	≈ (n.s.)	+50% (P = .105) [166]; P = .358 [167]	Probable (null)	—	[166,167]
Infill density beneath a solid skin	TPU, aramid/ASA, PLA, ABS, PA12, PEEK	Non-monotonic; ↓ 15–20% (running-in) [238]; TPU 2.15× at 50% vs 100% [107]	Non-monotonic or ↑	SWR 1.38× (n.s.) [244]	Conflicting	Structural compliance → contact pressure and area; heat conduction	[54,107,147,206,238,244,245,268,339]
Infill density, exposed or immersed	PLA, PLA-wood, PLA/CF, PEEK, PEBA	↑ (abrasion)	↓ (monotonic)	−56 to −68% volume loss, 20→80% [246]; P40/P80 1.73× [34]; −48% [149]	Established	Porosity; fluid uptake	[34,149,246–249]
Infill pattern, internal architecture, shell	PLA, ABS, LPLA, PA12, PEEK	Study-specific; auxetic lattices ↓	Study-specific; thicker shell ↓	Shell −41% [252]; erosion pattern share 64.8% [256]	Pattern conflicting (no transferable ranking); shell insufficient (low-appraisal studies only)	Subsurface stiffness; vibration damping; exposed texture	[15,123,128,241,252–256]

Parameter	Material classes studied	Typical direction of effect on COF	Typical direction of effect on wear	Magnitude range (within-study)	Consistency	Main pathway	Key refs
Nozzle temperature	PA, PLA, ABS, PETG, TPU, CF-ABS, carbon-PLA	Window; often n.s.	↓ up to the window, then ↑ or erratic	−75.5% SWR, 240→260 °C [39]; n.s. within datasheet ranges	Window probable; magnitude conflicting	Interbead healing; crystallinity; degradation	[26,27,38,39,132,148,207,241,257,260]
Bed/chamber temperature; print atmosphere	CF/PEEK, PETG	n.r.	Heated environment ↓; N_2 ↑	N_2 +58% SWR [51]	Bed/chamber insufficient; atmosphere probable (one study, mechanism measured)	Crystallinity; print-induced oxidation → transfer film	[51,63,64]
Print speed	ABS, PA12, PLA, PEEK, carbon-PLA	Steady state ≈ or ↑; running-in ↑ 6–19%	↑ (running-in, SWR)	Steady COF n.s. (20→50 mm/s) [64]; sole significant COF factor [257]	Conflicting (high-appraisal designs disagree)	Topography; interface thermal history	[64,139,238,239,257,258,342]
Energy density, laser power (PBF)	PA11, PA12/Cu, PA1212/GB/GF, UHMWPE, PA2200	↓ to an optimum	↓ while under-dense; ↑ above (filled powders)	−24% [137]; ≈10× with density [60]	Conflicting (material-specific window)	Porosity; filler dispersion; over-melting	[60,82,137,216,259,338]
Scan strategy (repeated scans), scan-vector length	h-BN/PEEK, MoS_2/PEEK, PA-GF	↑ with crystallinity	↓ with crystallinity	Wear 9.6–11.8× lower; COF 2.8–3.0× higher [40,87]	Probable (one group)	Crystallinity vs transfer-film formation	[40,87,327]
Powder refresh (recycled feedstock)	PA12/SiC	Slight ↑	Mixed (mass ↓, track ↑)	n.r.	Probable (material-specific)	Molecular weight, crystallinity (hypothesis)	[79]
VPP post-cure (presence, atmosphere, regime)	Dental methacrylates	n.r.	↓ with more complete cure	None vs 5 min: 1.93× [167]	Established (conversion pathway insufficient)	Cure state (conversion not measured with wear)	[92,167,171–173]
VPP build angle	Dental and engineering resins	Polished 75° ↓ 24% [88]	Direction inconsistent	1.10–1.43× (derived) [9,167,193]; up to 5.2× [181]; [165] excluded (SDs exceed means)	Conflicting (topographic explanation probable)	Stair-step topography; interlayer crack paths	[9,88,89,156,165,167,181,193]
Feedstock (brand, lot, colour)	PLA, PETG	Brand: 1.50× [42]	Colour and grade effects	COF 0.246–0.368 [42]	Conflicting (no effect in [51])	Additives, crystallinity	[42,51,66,132]

Note: MEX, material extrusion; PBF, powder bed fusion; VPP, vat photopolymerisation; n.r., not reported; n.s., not significant. Edge-on parallel/across: layer edges in the contact face, sliding parallel or perpendicular to the layer lines; face-on: layer plane as contact face; N: fibres normal to the sliding plane. Ratios and percentages not quoted verbatim are derived within-study comparisons and must not be compared across rows as absolute effects. Abstract-only studies [63,143,156,207,237,240,255,268,340–342] support direction only.
