# Supplementary material for: A Review of the Tribology of 3D-Printed Polymers and Polymer Composites
Source: Polymers (Basel). 2026 Nov 4;18(21):2747. doi: 10.3390/polym18212747 (PMC13766042; doi:10.3390/polym18212747)
Supplement: Supplementary file 1 [file polymers-18-02747-s001.zip › 05-PRISMA-Checklist.pdf]

PRISMA 2020 Checklist

Section and Topic	Item #	Checklist item	Location where item is reported
**TITLE**			
Title	1	Identify the report as a systematic review.	Title (identified as a review); Section 2.1 states that the work is a systematised critical review, not a full systematic review.
**ABSTRACT**			
Abstract	2	See the PRISMA 2020 for Abstracts checklist.	Abstract (objective, sources, number of studies, appraisal and grading, main findings, limitations and implications; MDPI single-paragraph format).
**INTRODUCTION**			
Rationale	3	Describe the rationale for the review in the context of existing knowledge.	Sections 1.1–1.3.
Objectives	4	Provide an explicit statement of the objective(s) or question(s) the review addresses.	Section 1.4 (framework and research questions RQ1–RQ8).
**METHODS**			
Eligibility criteria	5	Specify the inclusion and exclusion criteria for the review and how studies were grouped for the syntheses.	Section 2.2 (inclusion and exclusion criteria); grouping for the syntheses by material class and factor, Sections 2.5 and 16.
Information sources	6	Specify all databases, registers, websites, organisations, reference lists and other sources searched or consulted to identify studies. Specify the date when each source was last searched or consulted.	Section 2.1 (Web of Science and Europe PMC, last searched 29 September 2026; OpenAlex, last searched 30 September 2026); Section 2.2 (one study identified through expert knowledge); Figure 2.
Search strategy	7	Present the full search strategies for all databases, registers and websites, including any filters and limits used.	Section 2.1 (search concepts and fields); search notes in the PRISMA sheet of the evidence workbook (Supplementary Materials).
Selection process	8	Specify the methods used to decide whether a study met the inclusion criteria of the review, including how many reviewers screened each record and each report retrieved, whether they worked independently, and if applicable, details of automation tools used in the process.	Sections 2.2 and 2.6 (AI-assisted screening with all eligibility decisions checked by the authors); decision and reason for every record in the Screening log of the evidence workbook.
Data collection process	9	Specify the methods used to collect data from reports, including how many reviewers collected data from each report, whether they worked independently, any processes for obtaining or confirming data from study investigators, and if applicable, details of automation tools used in the process.	Sections 2.3 and 2.6; Full-text extraction sheet of the evidence workbook.
Data items	10a	List and define all outcomes for which data were sought. Specify whether all results that were compatible with each outcome domain in each study were sought (e.g. for all measures, time points, analyses), and if not, the methods used to decide which results to collect.	Section 2.3 (coefficient of friction, wear as reported and specific wear rate in $mm^3/(N·m)$); Section 11.3.
	10b	List and define all other variables for which data were sought (e.g. participant and intervention characteristics, funding sources). Describe any assumptions made about any missing or unclear information.	Section 2.3 (print parameters, orientation triplet, contact conditions, environment, roughness of both surfaces, replication and bulk state).
Study risk of bias assessment	11	Specify the methods used to assess risk of bias in the included studies, including details of the tool(s) used, how many reviewers assessed each study and whether they worked independently, and if applicable, details of automation tools used in the process.	Section 2.4 and Table S4 (five-criterion appraisal of reporting and evidential completeness; not a validated risk-of-bias instrument).
Effect measures	12	Specify for each outcome the effect measure(s) (e.g. risk ratio, mean difference) used in the synthesis or	Sections 2.5 and 16.3 (within-study ratios of treated

Section and Topic	Item #	Checklist item	Location where item is reported
		presentation of results.	to reference values); Table 7.
Synthesis methods	13a	Describe the processes used to decide which studies were eligible for each synthesis (e.g. tabulating the study intervention characteristics and comparing against the planned groups for each synthesis (item #5)).	Sections 2.5 and 16.1.
	13b	Describe any methods required to prepare the data for presentation or synthesis, such as handling of missing summary statistics, or data conversions.	Section 2.3 (values quoted only from full texts; implausible, predicted or unit-ambiguous values used for direction only; derived values labelled).
	13c	Describe any methods used to tabulate or visually display results of individual studies and syntheses.	Section 16; Tables 2–4, 6 and 7; Figures 10 and 11; Tables S1–S3 and S7–S9.
	13d	Describe any methods used to synthesize results and provide a rationale for the choice(s). If meta-analysis was performed, describe the model(s), method(s) to identify the presence and extent of statistical heterogeneity, and software package(s) used.	Sections 2.1, 2.5 and 16 (descriptive unweighted medians and ranges; no pooled effect estimated); rationale in Section 16.4.
	13e	Describe any methods used to explore possible causes of heterogeneity among study results (e.g. subgroup analysis, meta-regression).	Sections 16.2–16.4 (by factor, material class and contact configuration); Table S8.
	13f	Describe any sensitivity analyses conducted to assess robustness of the synthesized results.	Section 2.4 (four-criterion appraisal); Section 2.5 and Table S6 (sensitivity of the evidence grades); Section 16.3 and Table S7 (robustness of the ratio summaries).
Reporting bias assessment	14	Describe any methods used to assess risk of bias due to missing results in a synthesis (arising from reporting biases).	No formal assessment of reporting bias; representativeness of the selection, Section 2.2 and Table S5; association between effect size and citation rate, Section 16.3.
Certainty assessment	15	Describe any methods used to assess certainty (or confidence) in the body of evidence for an outcome.	Section 2.5 (evidence grades: established, probable, conflicting, insufficient) and Table S6.
**RESULTS**			
Study selection	16a	Describe the results of the search and selection process, from the number of records identified in the search to the number of studies included in the review, ideally using a flow diagram.	Section 2.2; Figure 2; PRISMA sheet of the evidence workbook.
	16b	Cite studies that might appear to meet the inclusion criteria, but which were excluded, and explain why they were excluded.	Section 2.2 (exclusion criteria) and Section 15.1; decision and reason for every excluded record in the Screening log of the evidence workbook.
Study characteristics	17	Cite each included study and present its characteristics.	Sections 5–15; Tables 1–6; Tables S1–S3; Included studies and Evidence matrix sheets of the evidence workbook.
Risk of bias in studies	18	Present assessments of risk of bias for each included study.	Table S4; Study appraisal sheet of the evidence workbook.
Results of individual studies	19	For all outcomes, present, for each study: (a) summary statistics for each group (where appropriate) and (b) an effect estimate and its precision (e.g. confidence/credible interval), ideally using structured tables or plots.	Tables S1–S3 and S8; Datasets S1 and S2; Full-text extraction sheet of the evidence workbook.
Results of syntheses	20a	For each synthesis, briefly summarise the characteristics and risk of bias among contributing studies.	Sections 16.1–16.2.
	20b	Present results of all statistical syntheses conducted. If meta-analysis was done, present for each the summary estimate and its precision (e.g. confidence/credible interval) and measures of statistical	Sections 16.2–16.3; Table 7; Figures 10 and 11

Section and Topic	Item #	Checklist item	Location where item is reported
		heterogeneity. If comparing groups, describe the direction of the effect.	(descriptive summaries; no meta-analysis).
	20c	Present results of all investigations of possible causes of heterogeneity among study results.	Sections 16.2–16.4; Table S8.
	20d	Present results of all sensitivity analyses conducted to assess the robustness of the synthesized results.	Sections 2.4, 2.5 and 16.3; Tables S6 and S7.
Reporting biases	21	Present assessments of risk of bias due to missing results (arising from reporting biases) for each synthesis assessed.	Not formally assessed (see item 14); Section 2.2, Table S5 and Section 16.3.
Certainty of evidence	22	Present assessments of certainty (or confidence) in the body of evidence for each outcome assessed.	Section 18.1 (evidence grade of each link in the framework); grades stated with the findings in Sections 5–17; Table 8.
**DISCUSSION**			
Discussion	23a	Provide a general interpretation of the results in the context of other evidence.	Sections 15–17 and 20.
	23b	Discuss any limitations of the evidence included in the review.	Section 18.2; Section 11.2 (reporting audit).
	23c	Discuss any limitations of the review processes used.	Section 2.2 (prioritised, partly access-driven selection); Sections 2.4–2.5 (author-defined appraisal and grading criteria applied by one reviewer).
	23d	Discuss implications of the results for practice, policy, and future research.	Sections 11.4, 19 and 20.
**OTHER INFORMATION**			
Registration and protocol	24a	Provide registration information for the review, including register name and registration number, or state that the review was not registered.	The review was not registered (Section 2.1).
	24b	Indicate where the review protocol can be accessed, or state that a protocol was not prepared.	No review protocol was prepared (Section 2.1).
	24c	Describe and explain any amendments to information provided at registration or in the protocol.	Not applicable.
Support	25	Describe sources of financial or non-financial support for the review, and the role of the funders or sponsors in the review.	Funding statement (no external funding; APC funded by the National University of Science and Technology POLITEHNICA Bucharest, PubArt programme).
Competing interests	26	Declare any competing interests of review authors.	Conflicts of Interest statement (none declared).
Availability of data, code and other materials	27	Report which of the following are publicly available and where they can be found: template data collection forms; data extracted from included studies; data used for all analyses; analytic code; any other materials used in the review.	Data Availability Statement and Supplementary Materials (Tables S1–S9, Datasets S1 and S2, and the evidence workbook with the screening log, extraction and appraisal data); analysis scripts are not published.

From: Page MJ, McKenzie JE, Bossuyt PM, Boutron I, Hoffmann TC, Mulrow CD, et al. The PRISMA 2020 statement: an updated guideline for reporting systematic reviews. BMJ 2021;372:n71. doi: 10.1136/bmj.n71. This work is licensed under CC BY 4.0. To view a copy of this license, visit https://creativecommons.org/licenses/by/4.0/
